# Addition of SHR-1701 to first-line capecitabine and oxaliplatin (XELOX) plus bevacizumab for unresectable metastatic colorectal cancer

**DOI:** 10.1038/s41392-024-02063-0

**Published:** 2024-12-16

**Authors:** Miao-Zhen Qiu, Yuxian Bai, Jufeng Wang, Kangsheng Gu, Mudan Yang, Yifu He, Cheng Yi, Yongdong Jin, Bo Liu, Feng Wang, Yu-kun Chen, Wei Dai, Yingyi Jiang, Chuanpei Huang, Rui-Hua Xu, Hui-Yan Luo

**Affiliations:** 1 Department of Medical Oncology, Sun Yat-sen University Cancer Center, State Key Laboratory of Oncology in South China, Guangdong Provincial Clinical Research Center for Cancer, Sun Yat-sen University, Guangzhou, PR China; 2 Research Unit of Precision Diagnosis and Treatment for Gastrointestinal Cancer, Chinese Academy of Medical Sciences, Guangzhou, PR China; 3 Department of Gastroenterology 1, Harbin Medical University Cancer Hospital, Harbin, PR China; 4 Department of Medical Oncology, The Affiliated Cancer Hospital of Zhengzhou University & Henan Cancer Hospital, Zhengzhou, PR China; 5 Oncology Ward 1, The First Affiliated Hospital of Anhui Medical University, Hefei, PR China; 6 Gastroenterology Ward (2), Shanxi Provincial Cancer Hospital, Taiyuan, PR China; 7 Medical Oncology Ward 1, Anhui Provincial Cancer Hospital, Hefei, PR China; 8 Abdominal Oncology, West China Hospital of Sichuan University, Chengdu, PR China; 9 Department of Medical Oncology, Sichuan Cancer Hospital & Institute, Sichuan Cancer Center, School of Medicine, University of Electronic Science & Technology of China, Chengdu, PR China; 10 Gastroenterology Ward 3, Cancer Hospital Affiliated to Shandong First Medical University, Jinan, PR China; 11 Oncology Department 1, The First Affiliated Hospital of Zhengzhou University, Zhengzhou, PR China; 12 Clinical Research & Development, Jiangsu Hengrui Pharmaceuticals Co., Ltd, Shanghai, PR China

**Keywords:** Gastrointestinal cancer, Tumour immunology

## Abstract

This phase 2/3 trial (NCT04856787) assessed the efficacy and safety of SHR-1701, a bifunctional protein targeting PD-L1 and TGF-β, in combination with BP102 (a bevacizumab biosimilar) and XELOX (capecitabine plus oxaliplatin) as a first-line treatment for unresectable metastatic colorectal cancer (mCRC). In this phase 2 study, a total of 62 patients with untreated, histologically confirmed colorectal adenocarcinoma and no prior systemic therapy for metastatic disease were enrolled. Patients received SHR-1701 (30 mg/kg), bevacizumab (7.5 mg/kg), and oxaliplatin (130 mg/m^2^) intravenously on day 1, along with oral capecitabine (1 g/m^2^ twice daily) on days 1–14 of 21-day cycles. Up to eight induction cycles were administered, followed by maintenance therapy for responders or those with stable disease. The primary endpoints were safety and objective response rate (ORR) per RECIST v1.1. The combination achieved an ORR of 59.7% and a disease control rate (DCR) of 83.9%. Median progression-free survival (PFS) was 10.3 months (95% CI: 8.3–13.7), with 6- and 12-month PFS rates of 77.2% and 41.3%, respectively. The estimated 12-month overall survival (OS) rate was 67.7%. Grade ≥3 treatment-related adverse events (TRAEs) were reported in 59.7% of patients, with anemia and neutropenia (8.1% each) being the most common. Retrospective DNA sequencing revealed that high tumor mutational burden, neo-antigens, and SBS15 enrichment correlated with better responses. Elevated baseline lactate dehydrogenase was linked to shorter PFS. SHR-1701 combined with XELOX and bevacizumab demonstrated a manageable safety profile and potent antitumor activity in unresectable mCRC.

## Introduction

Metastatic colorectal cancer (mCRC) continues to pose a significant clinical challenge due to its high mortality rate and limited treatment options. The National Comprehensive Cancer Network (NCCN) guidelines recommend first-line chemotherapy regimens such as FOLFOX (fluorouracil, leucovorin, and oxaliplatin), FOLFIRI (fluorouracil, leucovorin, and irinotecan), and XELOX (capecitabine and oxaliplatin), administered either alone or in combination with targeted therapies like bevacizumab (an anti-VEGF antibody) or cetuximab (an anti-EGFR antibody), which enhance efficacy by targeting specific molecular pathways.^1^ Despite these therapeutic strategies, the prognosis for patients with mCRC remains poor, with a median overall survival (OS) of approximately 30 months even when receiving first-line chemotherapy.^2^ This underscores the urgent need for novel treatment approaches to improve patient outcomes.

The advent of immunotherapy has revolutionized cancer treatment, offering new avenues for various malignancies, including colorectal cancer. Immune checkpoint inhibitors (ICIs), such as pembrolizumab and nivolumab, have shown remarkable efficacy in tumors exhibiting high microsatellite instability (MSI-H) or deficient mismatch repair (dMMR) due to their high mutational burden that enhances tumor immunogenicity.^3,4^ By inhibiting checkpoints such as PD-1/PD-L1 or CTLA-4, ICIs bolster T-cell-mediated immune responses against cancer cells. Consequently, several ICIs have received approval from the U.S. Food and Drug Administration (FDA) for use in both first-line and subsequent treatment for mCRC patients with MSI-H/dMMR status.^4^ However, MSI-H/dMMR tumors constitute only about 5% of advanced mCRC cases, leaving the majority of patients with proficient mismatch repair (pMMR) or microsatellite-stable (MSS) tumors, who derive limited benefit from ICIs as monotherapy.

To improve outcomes for patients with pMMR/MSS mCRC, recent studies have focused on combination therapies that might potentiate the efficacy of ICIs. Combining ICIs with chemotherapy, targeted therapies, or other immunomodulators is hypothesized to enhance antitumor immune responses by increasing neoantigen release and modulating the tumor microenvironment.^5,6^ Early clinical trials have shown encouraging results. For example, a phase 2 study reported that the combination of pembrolizumab with FOLFOX chemotherapy achieved an objective response rate (ORR) of 53% and a disease control rate (DCR) of 100% in untreated mCRC patients, irrespective of MMR status.^5^ Similarly, in a cohort of patients with *RAS*-mutated, MSS mCRC, the combination of durvalumab (an anti-PD-L1 antibody), tremelimumab (an anti-CTLA-4 antibody), and FOLFOX chemotherapy yielded an ORR of 62.5% and a DCR of 87.5%.^6^ These findings suggest that innovative first-line immunotherapy regimens may offer clinical benefits for patients with pMMR/MSS tumors.

SHR-1701 is a novel bifunctional fusion protein that represents a promising therapeutic strategy for patients with MSS/pMMR mCRC. It combines a monoclonal antibody against PD-L1 with the extracellular domain of transforming growth factor-beta receptor II (TGF-βRII), thereby concurrently inhibiting PD-L1-mediated immune checkpoint signaling and sequestering TGF-β ligands in the tumor microenvironment.^7,8^ Preclinical studies have demonstrated that SHR-1701 has high binding affinity for PD-L1, TGF-β1, and TGF-β3, effectively blocking both pathways.^7,8^ The TGF-β pathway is known to contribute to tumor progression and immune evasion by promoting tumor cell proliferation, invasion, and metastasis, as well as by creating an immunosuppressive microenvironment through the induction of regulatory T cells, suppression of effector T-cell function, and inhibition of natural killer (NK) cell activity.^9–13^ By simultaneously targeting PD-L1 and TGF-β, SHR-1701 aims to enhance antitumor immune responses and overcome mechanisms of immune resistance in mCRC.

The rationale for dual blockade of PD-L1 and TGF-β pathways stems from their synergistic roles in immune suppression within the tumor microenvironment. While PD-L1 expression on tumor cells inhibits T-cell activation and proliferation, TGF-β signaling further suppresses immune responses by promoting regulatory T-cell differentiation, inhibiting effector T-cell function, and fostering an immunosuppressive milieu. Dual inhibition is hypothesized to reinvigorate antitumor immunity more effectively than targeting either pathway alone, potentially overcoming resistance mechanisms in pMMR/MSS tumors.

Building upon this concept, we conducted the phase 2 portion of a phase 2/3 clinical trial to evaluate the efficacy and safety of SHR-1701 in combination with bevacizumab and XELOX chemotherapy as a first-line treatment for patients with unresectable mCRC. To our knowledge, this is the first study to assess a bifunctional anti-PD-L1/TGF-βRII agent in combination with standard chemotherapy and anti-angiogenic therapy in this patient population. This study aims to determine whether adding SHR-1701 to standard XELOX chemotherapy and bevacizumab can improve clinical outcomes in patients with unresectable MSS/pMMR mCRC, who currently have limited effective treatment options. Positive findings from this trial could introduce a novel therapeutic strategy for this challenging disease and potentially set the stage for future research into dual-pathway inhibition in colorectal cancer.

## Results

### Patients

**Fig. 1:**
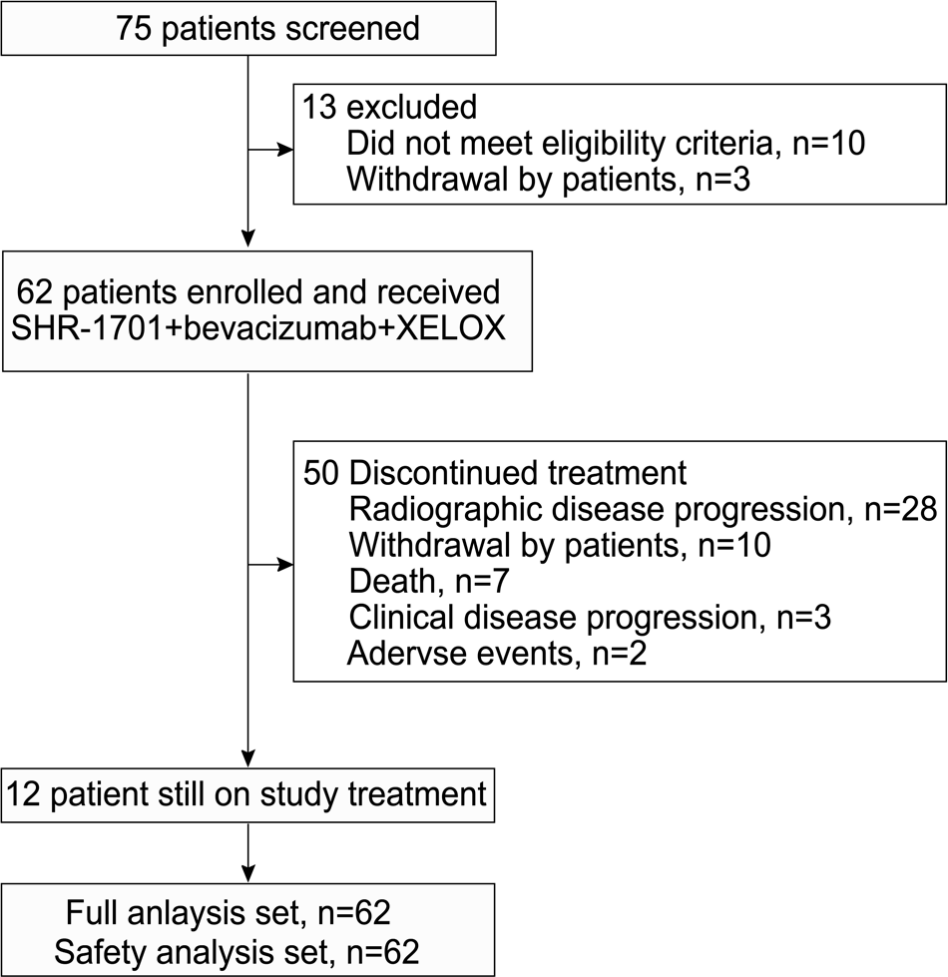
Study flow diagram. XELOX, capecitabine plus oxaliplatin

Between June 22, 2021, and April 12, 2022, 62 patients were enrolled in the study and initiated treatment with SHR-1701, bevacizumab, and XELOX (Fig. 1). As of June 21, 2023, the median follow-up period was 16.0 months. At that time, 12 patients (19.4%) were still undergoing study treatment, while 50 patients (80.6%) had discontinued. The primary reasons for discontinuation included radiological disease progression and patient withdrawal.

By the end of the treatment period, 24 patients (38.7%) had transitioned to one or more subsequent systemic anticancer therapies (Supplementary Table 1). The median age of patients was 56 years, with 36 (58.1%) being male. Right-sided mCRC was present in 22 patients (35.5%), and liver metastases were observed in 43 patients (69.4%). All patients had tumors classified as MSS or pMMR (Table 1).

**Table 1:** Demographics and baseline characteristics

Characteristic	All patients (*n* = 62)
Age, years, median (range)	56 (23–73)
Sex, *n* (%)	
Male	36 (58.1)
Female	26 (41.9)
BMI, kg/m^2^, mean ± SD	22.8 ± 3.2
Primary tumor location, *n* (%)	
Colon	40 (64.5)
Rectum	22 (35.5)
Tumor site, *n* (%)	
Left or rectum	40 (64.5)
Right	22 (35.5)
MSS/pMMR, *n* (%)	62 (100.0)
Surgery on primary tumor, *n* (%)	
Radical surgery	26 (41.9)
Palliative surgery	13 (21.0)
Diagnostic procedure	2 (3.2)
Previous (neo) adjuvant therapy, *n* (%)	12 (19.4)
ECOG performance status, *n* (%)	
0	17 (27.4)
1	45 (72.6)
Metastasis sites, *n* (%)	
Liver	43 (69.4)
Peritoneum	12 (19.4)
*RAS* gene status, *n* (%)^a^	
Wild-type	24 (38.7)
Mutant-type	36 (58.1)
*BRAF* gene status, *n* (%)^a^	
Wild-type	47 (75.8)
Mutant-type	4 (6.5)
*RAS*/*BRAF*, *n* (%)^a^	
*RAS* and *BRAF* wild-type	11 (17.7)
*RAS* or *BRAF* mutant-type	40 (64.5)
PD-L1 expression, *n* (%)^a,b^	
Positive (TPS ≥ 1% or CPS ≥ 1)	33 (53.2)
Negative	26 (41.9)
TGF-β1, *n* (%)^a^	
Positive	10 (16.1)
Negative	9 (14.5)
pSMAD 2/3 expression, *n* (%)^a^	
≥80%	10 (16.1)
<80%	9 (14.5)

^a^The status was not detected in remaining patients

^b^ PD-L1 positive was defined as TPS ≥ 1% or CPS ≥ 1; PD-L1 negative was defined as any values other than TPS ≥ 1% or CPS ≥ 1 in cases with CPS or TPS detection

*BMI* body mass index, *SD* standard deviation, *ECOG* Eastern Cooperative Oncology Group, *MSS* microsatellite stable, *pMMR* mismatch repair-proficient, *PD-L1* programmed death-ligand 1, *TPS* tumor proportion score, *CPS* combined positive score

### Efficacy

**Fig. 2:**
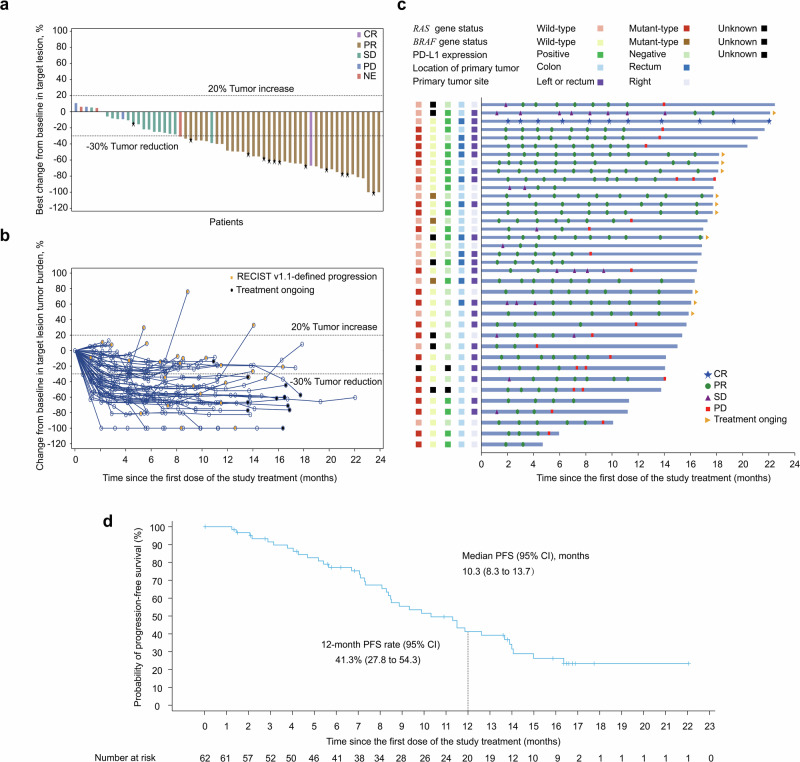
Antitumor activity of SHR-1701 in combination with bevacizumab and XELOX. Responses were assessed by investigator per RECIST V1.1. **a** Best change of target lesions from baseline in each patient. **b** Percentage change from baseline in target lesion tumor burden over time. **c** Survival time and duration of tumor response in 37 responders. The thick blue lines indicate the duration of survival follow-up. **d** Kaplan-Meier plot of PFS in all 62 patients. Crosses denote censored patients. PD, progressive disease; CR, complete response; PR, partial response; RECIST, Response Evaluation Criteria in Solid Tumors; SD, stable disease; NE, not evaluated. PFS, progression-free survival; CI, confidence interval

The ORR for SHR-1701 in combination with bevacizumab and XELOX was 59.7% (37/62; 95% CI, 47.3–71.0) (Table 2). Of the 62 patients, thirty-seven (59.7%; 95% CI, 47.3–71.0) achieved an objective response, consisting of one (1.6%) CR and 36 (58.1%) PR. The DCR was 83.9% (52/62; 95% CI, 72.8–91.0). In total, 53 patients (85.5%) exhibited a reduction in the size of their target lesions compared to baseline (Fig. 2a). Tumor burden reductions were maintained across multiple time points, and responses were ongoing in 16 of the 37 responders (43.2%) at the time of analysis. The median DoR was 10.7 months (Fig. 2b, c).

**Table 2:** Tumor response and survival outcomes

Parameters	All patients (*n* = 62)
Best overall response, *n* (%)	
Complete response	1 (1.6%)
Partial response	36 (58.1%)
Stable disease	15 (24.2%)
Progressive disease	3 (4.8%)
Not evaluated	7 (11.3%)
Objective response rate ^a^, *n* (%) [95% CI]	37 (59.7%) [47.3–71.0]
Disease control rate, *n* (%) [95% CI]	52 (83.9%) [72.8–91.0]
Duration of response ^a^	
Ongoing response, n/N (%)	37/62 (59.7%)
Median (95% CI), months	10.7 (8.4–13.0)
6-month rate, % (95% CI)	79.1 (61.1–89.5)
12-month rate, % (95% CI)	40.8 (23.8–57.2)
Progression-free survival	
Events, n/N (%)	38/62 (61.3%)
Median (95% CI), months	10.3 (8.3–13.7)
6-month rate, % (95% CI)	77.2 (64.0–86.1)
12-month rate, % (95% CI)	41.3 (27.8–54.3)

^a^ Complete or partial responses were confirmed

*CI* confidence interval

At the data cutoff, 38 patients (61.3%) had experienced disease progression or died. The median PFS was 10.3 months (95% CI, 8.3–13.7), with PFS rates of 77.2% (95% CI, 64.0–86.1) at 6 months and 41.3% (95% CI, 27.8–54.3) at 12 months (Fig. 2e). A total of 25 patients (40.3%) had died. The OS data were still immature, with an estimated 12-month OS rate of 67.7% (95% CI, 54.6–77.8).

### Subgroup and blood biomarker analysis

**Fig. 3:**
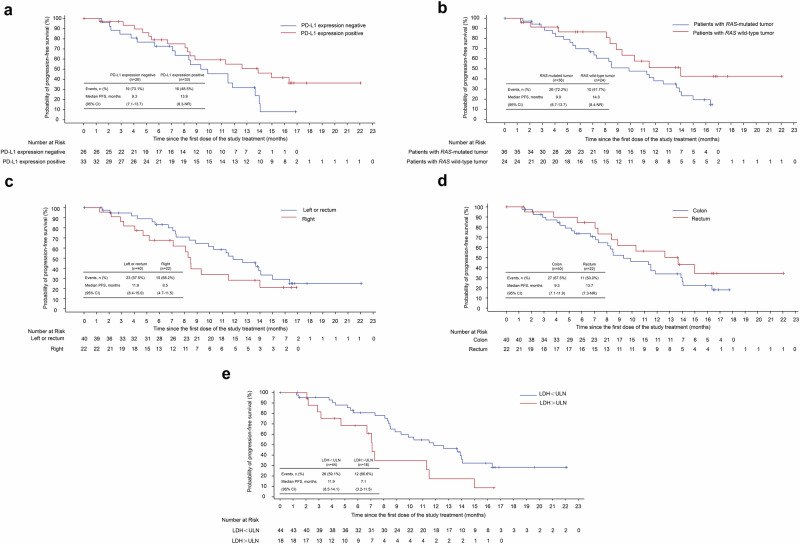
Kaplan-Meier curves for PFS in the subgroups. **a** PFS in patients with tumor PD-L1-positive (CPS ≥ 1 or TPS ≥ 1%) and those with tumor PD-L1-negative. **b** PFS in patients with *RAS* gene status of the mutant-type and those with *RAS* gene status of the wild-type. **c** PFS in patients with a primary tumor site on the left or rectum and those with a primary tumor site on the right. **d** PFS in patients with the location of the primary lesion in the colon and those with the location of the primary lesion in the rectum. **e** PFS in patients with the LDH < ULN compared to those with LDH > ULN. PD-L1 positive was defined as TPS ≥ 1% or CPS ≥ 1; PD-L1 negative was defined as any values other than TPS ≥ 1% or CPS ≥ 1 in cases with CPS or TPS detection. PFS, progression-free survival; CI, confidence interval; NR, not reached; PD-L1, programmed death-ligand 1, TPS, tumor proportion score. ULN, upper limit of normal

To identify patients most likely to benefit from SHR-1701 combined with bevacizumab and XELOX, we evaluated responses across various subgroups and examined baseline biomarkers from complete blood counts and biochemistry panels. The analysis assessed ORR and PFS based on factors including PD-L1 expression, *RAS/BRAF* mutation status, primary tumor characteristics, TGF-β1 levels, pSMAD2/3 expression, and lactate dehydrogenase (LDH) levels. The findings are detailed in Fig. 3a–e and Supplementary Table 2.

Among 33 patients with PD-L1-positive tumors, the ORR was 57.6%, similar to the 61.5% observed in 26 PD-L1-negative patients. However, PD-L1-positive patients demonstrated a longer median PFS (13.9 vs. 9.3 months). For *RAS*-mutated tumors (*n* = 36), the ORR was 58.3% (95% CI: 42.2–72.9) and the median PFS was 9.9 months (95% CI: 6.7–13.7). In comparison, *RAS* wild-type tumors (*n* = 24) showed an ORR of 62.5% (95% CI: 42.7–78.8) and a median PFS of 14.0 months (95% CI: 8.4–NR). Patients with high pSMAD2/3 expression (≥80%, *n* = 10) had an ORR of 50.0% (95% CI: 23.7–76.3) and a median PFS of 8.5 months (95% CI: 2.1–13.9). In contrast, those with pSMAD2/3 levels below 80% (*n* = 9) had an ORR of 44.4% (95% CI: 18.9–73.3) and a median PFS of 7.3 months (95% CI: 4.7–8.5). These post hoc analyses used 80% as the median cutoff for the 19 patients.

In the biomarker analysis, elevated LDH levels above the upper limit of normal (ULN) were associated with significantly shorter PFS (P < 0.05, log-rank test). Patients with LDH > ULN (*n* = 18) achieved an ORR of 38.9% (95% CI: 20.3–61.4) and a median PFS of 7.1 months (95% CI: 3.2–11.5). In contrast, those with LDH ≤ ULN (*n* = 44) had an ORR of 68.2% (95% CI: 53.4–80.0) and a median PFS of 11.9 months (95% CI: 8.5–14.1).

### Somatic alterations and SHR-1701 response in mCRC

To explore the connection between genomic features and clinical outcomes with SHR-1701, we conducted a retrospective analysis using formalin-fixed paraffin-embedded (FFPE) samples from 14 patients treated at a single center. The clinical characteristics of the biomarker-evaluable population (BEP) are detailed in Supplementary Table 3. Among these 14 patients, 7 (50.0%) achieved a PR, 5 (35.7%) demonstrated SD, and 2 (14.3%) were classified as not evaluable (NE). The median PFS for this cohort was 8.1 months (95% CI: 6.0–10.2).

**Fig. 4:**
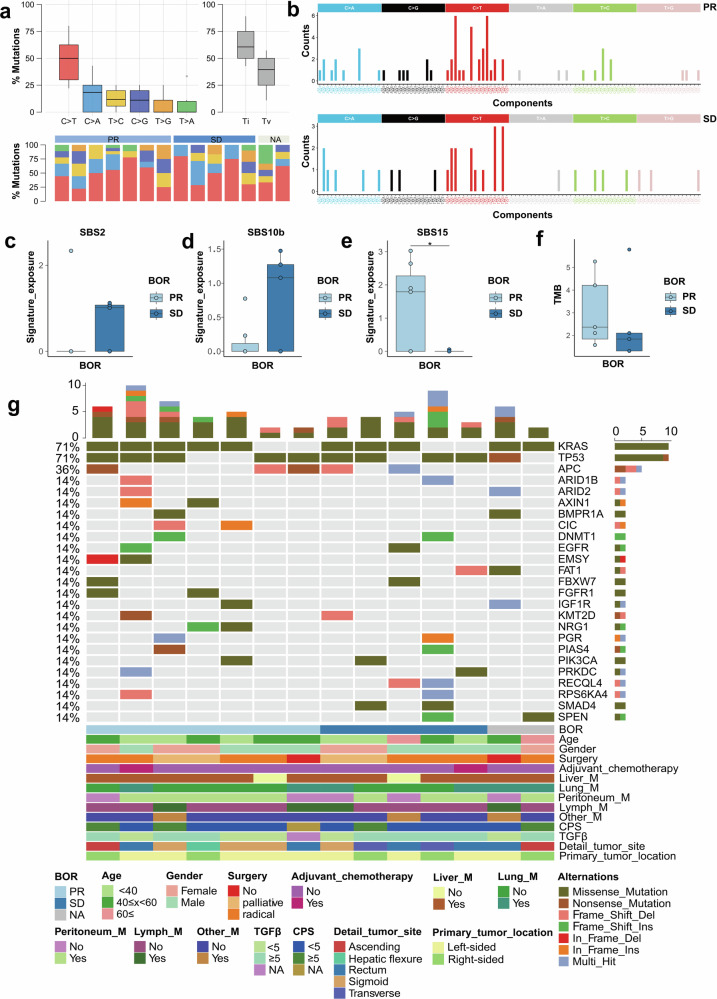
Genomic alterations and clinical response to SHR-1701 in combination with bevacizumab and XELOX. **a** Summary of the distribution of SNVs among the SHR-1701 cohorts. **b** Mutational spectrum of the SHR-1701 genome in the BEP cohort. Substitutions are plotted in different colors, with their context arranged as indicated. **c**–**e** Boxplot comparisons of Single Base Substitutions (SBS2, SBS10b, SBS15) between patients achieving a PR and those with SD. **f** Boxplot comparison of TMB between the PR and SD groups. **g** Somatic mutational landscape of the SHR-1701 cohort. Each gene (row) and its corresponding alterations in each tumor sample (column) are represented as a heatmap based on the color legend provided below. Samples are arranged according to the number of clinical annotations, which are displayed in the bottom panel. Clinical parameters for each patient are shown beneath the heatmap, while alteration frequencies are illustrated in the right panel. The total number of alterations in each sample is summarized in the upper panel. SNV, single nucleotide variant; BEP, biomarker-evaluable population; SBS, Single Base Substitution; PR, partial response; SD, stable disease; TMB, tumor mutation burden

A notable predominance of C > T substitutions was identified, accounting for 51.4% of all single nucleotide variations (SNVs) within the cohort (Fig. 4a, b). Additionally, we analyzed the mutational profiles of the SHR-1701 cohort against the COSMIC SBS signatures and calculated the corresponding signature exposure scores (Supplementary Fig. 1a, b). The findings revealed an upregulation of SBS2 and SBS10b in patients with SD (Fig. 4c, d). SBS2, which reflects the activity of the APOBEC family of cytidine deaminases, is known to drive mutation accumulation and elevate cancer risk.^14^ Moreover, SBS15 was significantly enriched in patients who achieved a PR (*p* = 0.038, Fig. 4e). While the PR group exhibited higher tumor mutational burden (TMB), the difference did not reach statistical significance, likely due to the limited sample size (Fig. 4f).

To investigate potential mutations and genes linked to a selective response to SHR-1701, we mapped the genomic landscape of the cohort. The most frequently mutated genes were *KRAS*, *TP53*, and *APC*, aligning with patterns observed in previously reported CRC cohorts (Fig. 4g). Additionally, we examined the genomic integrity of the TGF-β signaling pathway by analyzing SNV and CNV events in key regulatory genes such as *SMAD2*, *SMAD4*, *ACVR2A*, *TGFBR1*, and *TGFBR2*. Notably, *SMAD4*, a critical mediator activated by transmembrane serine-threonine receptor kinases in the TGF-β signaling pathway, was mutated in two patients from the SD group. These patients also exhibited widespread deletions in other core genes of the TGF-β pathway. These findings suggest that disruptions in the TGF-β pathway could play a significant role in modulating the response to SHR-1701 (Supplementary Fig. 1c, d).

To detect somatic copy number alterations (SCNAs) and evaluate the cohort’s CNV burden, we utilized GISTIC2.0. This analysis corroborated previously reported SCNA patterns in CRC cohorts, including chromosomal gains on 1, 6p, 7q, 10q, and 20p, as well as losses on 1p, 6p, and 22q (Supplementary Fig. 1e).^15^ Additionally, we performed neo-antigen prediction for each sample to assess their potential role in immunogenicity and their correlation with the response to SHR-1701. Recurrent immunogenic peptides, derived from mutations in genes such as *APC*, *IGF1R*, and *KRAS*, were identified (data not shown). Although the neo-antigen burden was higher in the PR group compared to the SD group, the difference was not statistically significant (Supplementary Fig. 1f).

### Safety

The median treatment durations were 27.0 weeks (interquartile range [IQR]: 15.0–45.0) for SHR-1701, 30.0 weeks (IQR: 15.0–46.5) for bevacizumab, 30.5 weeks (IQR: 16.1–48.0) for capecitabine, and 21.0 weeks (IQR: 15.0–24.0) for oxaliplatin. Nearly all patients (61/62, 98.4%) experienced at least one TRAE (Table 3). The most commonly reported TRAEs were anemia (*n* = 38, 61.3%), nausea (*n* = 34, 54.8%), vomiting (*n* = 32, 51.6%), and increased aspartate aminotransferase (*n* = 31, 50.0%). Grade 3 or higher TRAEs occurred in 37 patients (59.7%). The most frequent grade ≥3 events included anemia (*n* = 5, 8.1%) and decreased neutrophil count (*n* = 5, 8.1%), followed by anaphylactic reaction (*n* = 3, 4.8%).

**Table 3:** Treatment-related adverse events

TRAEs, n (%)	All patients (*n* = 62)			
	Grade 1–2	Grade 3	Grade 4	Grade 5
Patients with at least one TRAE	24 (38.7)	27 (43.5)	5 (8.1)	5 (8.1)
TRAEs (all grades) in ≥15% of patients:				
Anemia	33 (53.2)	5 (8.1)	0	0
Nausea	33 (53.2)	1 (1.6)	0	0
Vomiting	32 (51.6)	0	0	0
Aspartate aminotransferase increased	31 (50.0)	0	0	0
Decreased appetite	29 (46.8)	0	0	0
Hypoesthesia	29 (46.8)	0	0	0
Platelet count decreased	24 (38.7)	1 (1.6)	0	0
Alanine aminotransferase increased	22 (35.5)	1 (1.6)	0	0
Epistaxis	22 (35.5)	0	0	0
Diarrhea	21 (33.9)	2 (3.2)	0	0
Proteinuria	21 (33.9)	0	0	0
Neutrophil count decreased	19 (30.6)	5 (8.1)	0	0
Hypoalbuminemia	19 (30.6)	1 (1.6)	0	0
White blood cell count decreased	19 (30.6)	0	0	0
Gingival bleeding	17 (27.4)	0	0	0
Blood bilirubin increased	15 (24.2)	1 (1.6)	0	0
Pruritus	13 (21.0)	0	0	0
Malaise	12 (19.4)	2 (3.2)	0	0
Occult blood positive	12 (19.4)	0	0	0
Mouth hemorrhage	12 (19.4)	0	0	0
Dizziness	12 (19.4)	0	0	0
Fatigue	11 (17.7)	2 (3.2)	0	0
Constipation	11 (17.7)	0	0	0
Hypothyroidism	11 (17.7)	0	0	0
Palmar-plantar erythrodysesthesia syndrome	10 (16.1)	2 (3.2)	0	0
Rash	10 (16.1)	0	0	0

TRAEs, treatment-related adverse events

Study treatment was discontinued due to TRAEs in 13 (21.0%) patients, with each AE occurring in a single patient (Supplementary Table 4). Investigator assessments revealed that 21 patients (33.9%) experienced irAEs of any severity (Supplementary Table 5). Nine patients (14.5%) experienced grade 3 or higher irAEs, including 2 cases of elevated gamma-glutamyltransferase; all other such irAEs occurred in only one patient each. Grade 3 or higher treatment-related SAEs occurred in 17 patients (27.4%), with pneumonia reported in 2 cases; all other grade 3 or higher SAEs were seen in only one patient each (Supplementary Table 6). Among the five treatment-related deaths (pneumonia, *n* = 2; multiple organ dysfunction syndrome, *n* = 1; small intestinal obstruction, *n* = 1; unknown cause, *n* = 1), the pneumonia and unknown cause deaths may be potentially associated with SHR-1701 exposure.

## Discussion

This study represents the first to evaluate a bifunctional anti-PD-L1/TGF-βRII agent, SHR-1701, in combination with chemotherapy (XELOX) and bevacizumab as a first-line treatment for unresectable mCRC. The regimen met its primary endpoint, achieving an ORR of 59.7% in a challenging patient population. Notably, 85.5% of patients who underwent post-baseline scans showed reductions in target lesion size. Responses were durable, with a 12-month DoR probability of 40.8% and a median PFS of 10.3 months. Although OS data are still maturing, the estimated 12-month OS rate was 67.7%.

Results from other first-line ICI-based trials provide useful context. In the Checkmate 9X 8 trial, which compared FOLFOX plus bevacizumab with or without nivolumab, the ORR was 60% in the nivolumab arm versus 46% in the standard-of-care (SOC) arm.^16^ The AtezoTRIBE trial, which added atezolizumab to FOLFOXIRI and bevacizumab, reported an ORR of 59% for the triplet regimen compared to 64% for FOLFOXIRI plus bevacizumab.^17^ Both trials predominantly enrolled patients with pMMR tumors. In our study, which also focused on patients with MSS or pMMR tumors, the ORR of 59.7% aligns well with these findings, demonstrating that SHR-1701 offers comparable efficacy in this population.

In terms of PFS, Checkmate 9X 8 reported no significant improvement in either the intention-to-treat population or the pMMR subgroup when nivolumab was added to FOLFOX and bevacizumab.^16^ Conversely, AtezoTRIBE showed a numerical, though not statistically significant, PFS improvement with atezolizumab (12.9 vs. 11.4 months) in the pMMR group.^17^ The CAPability 01 trial, which examined sintilimab with chidamide and bevacizumab in MSS/pMMR mCRC, reported an 18-week PFS rate of 64% in the triplet arm versus 21.7% in the doublet arm.^18^ These findings are consistent with our phase 2 results, which reported a median PFS of 10.3 months and an estimated 12-month OS rate of 67.7%. Differences in patient demographics, genetic backgrounds, and trial designs, however, warrant caution when making direct comparisons.

Our study also explored potential biomarkers for predicting treatment efficacy. In line with previous study,^8^ patients with positive PD-L1 expression achieved similar ORR (57.6% vs. 61.5%) but experienced longer PFS (13.9 vs. 9.3 months) than those with negative PD-L1 expression. This suggests that PD-L1 expression may serve as a partial predictor of response. However, the Checkmate 9X8 trial found the opposite, with shorter PFS in patients with higher PD-L1 expression.^16^ These discrepancies may stem from differences in patient populations and PD-L1 detection methods.

*RAS* gene status also emerged as a potential predictor. Patients with wild-type *RAS* tumors demonstrated a higher ORR (62.5% vs. 58.3%) and longer PFS (14.0 vs. 9.9 months) than those with mutated *RAS*. The AVETRIC trial reported similar results with a modified FOLFOXIRI regimen combined with cetuximab and avelumab, achieving an ORR of 82% and a PFS of 14.1 months in RAS wild-type mCRC.^19^ Similarly, the BBCAPX trial explored the combination of sintilimab, bevacizumab, oxaliplatin, and capecitabine as first-line therapy in *RAS*-mutant, MSS, unresectable mCRC and reported a median PFS of 9.9 months.^20^ These findings highlight the potential of tailoring therapy based on RAS status.

Previous studies have shown that CRC patients with oncogenic TGF-β pathway alterations have shorter OS compared to those without such mutations.^15^ SMAD2 and SMAD3, key components of this pathway, form nuclear complexes with SMAD4 to regulate gene expression, influencing tumor progression and immune responses.^21,22^ Our analysis identified a positive correlation between higher baseline pSMAD2/3 levels and better clinical outcomes, with improved ORR (50.0% vs. 44.4%) and longer PFS (8.5 vs. 7.3 months) for patients with an H-score ≥80% compared to <80%. These findings suggest that SHR-1701 may partly exert its therapeutic effect by inhibiting the SMAD2-dependent TGF-β pathway, which contributes to tumor progression and immune suppression. While promising, further studies are needed to determine whether this correlation predicts response to SHR-1701 or reflects general prognostic trends.

Elevated baseline LDH levels were associated with poorer outcomes, consistent with its known role as a prognostic marker in various cancers.^23^ In our study, patients with LDH levels above the ULN had shorter PFS compared to those with LDH levels below ULN. Similar trends have been observed in non-small cell lung cancer and small-cell lung cancer.^24,25^

In our study, integrative DNA sequencing (DNA-seq) was performed on 14 patients from a single center to investigate the relationship between genomic alterations and clinical response to SHR-1701. TMB, a known marker of tumor immunogenicity and predictor of ICI response across cancers,^26^ was compared between the PR and SD groups. Although TMB and SBS15 levels were higher in the PR group, the small sample size precluded statistical significance. The APOBEC signature, previously associated with poor outcomes in lung cancer due to APOBEC-related mutation accumulation,^27^ was also examined. Consistent with prior studies, the SBS2 signature, linked to APOBEC enzyme activity, was upregulated in the SD group. Recognizing that cancer immunogenicity stems largely from variant peptides,^15^ we predicted neo-antigens for each sample, which similarly showed a trend supporting the clinical benefit of SHR-1701. These findings suggest that established ICI response predictors, such as TMB and APOBEC signatures, could be relevant for forecasting SHR-1701 efficacy.

The incidence and severity of TRAEs associated with SHR-1701, bevacizumab, and XELOX were consistent with the established toxicity profiles of chemotherapy and immunotherapy, with no new safety concerns observed.^7,8,28,29^ Grade 3 or higher TRAEs were most commonly anemia, decreased neutrophil count, and anaphylactic reactions. Interestingly, treatment-induced anemia occurred more frequently in mCRC patients receiving SHR-1701 (53.2%) compared to patients with other tumor types (8.6–12.3%).^8,30,31^ This difference may stem from tumor-specific factors, the concurrent use of bevacizumab, or other related complications. Previous studies have linked TGF-β inhibitors, such as bintrafusp alfa, to specific skin-related adverse events, including squamous cell carcinoma (SCC) and keratoacanthoma, which affected 4% and 8% of patients, respectively, with some experiencing severe Grade 3 or higher reactions.^32–35^ In contrast, no cases of skin SCC or keratoacanthoma were observed in our study involving SHR-1701 in combination therapy. This could be partly due to the small sample size or the low incidence of these events. Furthermore, two phase 1 trials of SHR-1701 monotherapy in other advanced solid tumors also reported no such adverse events.^7,8^ Several factors, including differences in skin pigmentation, ultraviolet exposure, patient demographics, and varying drug mechanisms, may account for the discrepancies in skin toxicity between bintrafusp alfa and SHR-1701.^35–38^ Continued monitoring will be essential to identify any potential cutaneous adverse events in future studies.

This study has several limitations. The absence of a control arm limits direct comparisons to standard-of-care therapies. Additionally, the OS data remain immature, necessitating longer follow-up. Subgroup and biomarker analyses were constrained by the small sample size, limiting the generalizability of findings. Future studies with larger cohorts and longer follow-up are needed to confirm these results.

In summary, SHR-1701, in combination with XELOX and bevacizumab, demonstrated promising efficacy and manageable safety as a first-line treatment for mCRC patients with MSS or pMMR tumors.

## Methods

### Study design and patients

This phase 2/3 clinical trial (NCT04856787) was conducted across 10 study sites in China (Supplementary Table 7). The phase 2 portion was a single-arm study aimed at assessing the efficacy and safety of SHR-1701 in combination with bevacizumab and XELOX as a first-line treatment for patients with unresectable mCRC. The phase 3 portion, designed as a randomized, double-blind, placebo-controlled trial, evaluated the combination of SHR-1701 or placebo alongside bevacizumab and XELOX in a comparable patient group. This report focuses exclusively on findings from the phase 2 portion of the study.

Eligible patients were aged 18 to 75 years, with histologically confirmed, unresectable colorectal adenocarcinoma. They had not previously received systemic therapy for recurrent or metastatic disease and demonstrated an ECOG performance status of 0 or 1. Inclusion required at least one measurable lesion as defined by RECIST v1.1, a minimum life expectancy of three months, and adequate organ and bone marrow function. Patients also needed to provide archived or fresh tumor tissue samples. Patients who had undergone prior neoadjuvant or adjuvant therapy were eligible, provided they had remained recurrence-free for at least 12 months. Key exclusion criteria included the presence of recurrent or metastatic lesions suitable for radical surgery, central nervous system or meningeal metastases, tumors with dMMR/MSI-H status, and active or historical autoimmune diseases. Additional exclusions encompassed prior treatment with PD-1, PD-L1, CTLA-4, anti-EGFR, or anti-angiogenic therapies, use of immunosuppressive medications or systemic corticosteroids at immunosuppressive doses within 14 days, and a diagnosis of interstitial pneumonia or lung disease. Comprehensive eligibility details are outlined in the study protocol.

The study received ethical approval from the ethics committees of all participating sites and adhered to the Declaration of Helsinki and Good Clinical Practice standards. Written informed consent was obtained from all patients.

### Procedure

Patients were administered intravenous SHR-1701 (30 mg/kg), bevacizumab (7.5 mg/kg), and oxaliplatin (130 mg/m^2^) on day 1 of each 21-day cycle. Additionally, oral capecitabine (1 g/m^2^, twice daily) was given on days 1–14 of the cycle. Up to eight cycles of induction therapy were planned. Patients who demonstrated an objective response or maintained stable disease (SD) transitioned to maintenance therapy, which included SHR-1701 (30 mg/kg on day 1), bevacizumab (7.5 mg/kg on day 1), and capecitabine (1 g/m^2^, twice daily on days 1–14) within the same 21-day cycle. This maintenance phase continued until disease progression, intolerable toxicity, the initiation of a new anticancer therapy, patient withdrawal, or a decision by the investigator, whichever occurred first. For patients showing radiological PD, continuation of maintenance therapy was permitted at the investigator’s discretion if clinical benefit and tolerability were evident. The maximum cumulative treatment duration for SHR-1701, bevacizumab, or capecitabine capped at two years.

### Assessments

Radiographic evaluations were performed at baseline, then every six weeks for the first 48 weeks, and subsequently every 12 weeks. Clinical responses were assessed by investigators in accordance with RECIST v1.1 criteria. CR and PR required confirmation via a follow-up scan conducted at least four weeks later. Patients who discontinued treatment without documented radiological progression continued tumor response evaluations based on the predetermined schedule until disease progression, initiation of a new anticancer therapy, withdrawal of consent, loss to follow-up, or death, whichever occurred first. For patients who met RECIST v1.1 criteria for PD and discontinued study treatment, no further radiographic assessments were planned. However, if the investigator suspected pseudoprogression and the study treatment was continued, disease progression had to be confirmed through a follow-up scan conducted at least four weeks later. Tumor PD-L1 expression was evaluated locally using the combined positive score (CPS) or tumor proportion score (TPS). Immunohistochemistry (IHC) was used to assess TGF-β1 expression and SMAD2 phosphorylation, employing a monoclonal anti-TGF-β1 antibody (ab190503, Abcam) and a monoclonal anti-phospho-SMAD2 antibody (138D4, Cell Signaling Technology).

### Outcomes

The primary endpoints were the investigator-assessed ORR, defined as the proportion of patients whose best overall response (BOR) was confirmed as either CR or PR, and the safety profile of the treatment. Secondary endpoints included the DCR, which measured the proportion of patients achieving CR, PR, or SD as their BOR; duration of response (DoR), defined as the time from the first recorded objective response to either disease progression or death from any cause, whichever occurred first; PFS, calculated from the initiation of study treatment to the first documented progression or death from any cause; and OS, measured from the start of study treatment to death from any cause.

### Gene panel sequencing

The capture-based sequencing panel utilized in this study was developed and supplied by Precision Scientific (Beijing) Co., Ltd. This panel includes 418 genes commonly mutated in solid tumors and relevant to precision oncology. It covers 156 cancer driver genes, 39 cancer predisposition genes, 39 genes involved in critical DNA damage repair pathways, 95 genes with actionable mutations for targeted therapies, and 62 genes linked to immuno-oncology (Supplementary Table S8). The panel’s probes target exonic regions of all genes as well as specific hotspots within intronic and promoter regions, enabling a comprehensive mutational analysis for solid tumors.

Raw sequencing data were processed for quality control using TrimGalore version 0.6.10, which incorporates a default decompression path feature (Krueger, 2023). Reads were then mapped to the hg38 reference genome using BWA-MEM (v0.7.17-r1188).^39^ Subsequent steps, including sorting, duplicate removal, and base quality score recalibration, were performed using SAMtools (v1.9) and the GATK toolkit (v4.2.6.1).^40,41^

The GATK somatic short variant discovery workflow was employed to detect somatic mutations, including SNVs and small insertions or deletions (indels). Exonic regions of the target genes were extracted and merged based on the GTF file from Gencode (v45). Gene synonyms were manually updated to their latest nomenclature using Ensembl, and the corresponding exon regions were merged accordingly (Supplementary Table 8). Somatic mutations were annotated using Vcf2maf version 1.6.19 in conjunction with Ensembl Variant Effect Predictor (VEP) version 105, and the annotated VCF files were subsequently converted to MAF format (mskcc/vcf2maf: v1.6, 2020).

TMB was calculated as the number of somatic mutations per megabase (Mb) of the coding region, encompassing mutation types such as Frame_Shift_Del, Frame_Shift_Ins, In_Frame_Del, In_Frame_Ins, Missense_Mutation, Nonsense_Mutation, Nonstop_Mutation, and Splice_Site mutations.

Copy number alterations (CNAs) were identified using CNVkit (v0.9.10), which performed binning, coverage calculation, copy ratio normalization, and segmentation.^42^ The same target regions used for somatic mutation detection were applied in the CNA analysis. To pinpoint significantly amplified or deleted genomic regions, GISTIC2 (v2.0.23) was utilized on the segmented data.

GISTIC2 (v2.0.23) was applied to segmentation data to identify genomic regions with significant amplifications or deletions.^43,44^ Mutational profiles in MAF format were matched against the COSMIC SBS signature database to calculate signature exposure scores and proportions.^45^ SBS signatures contributing less than 5% on average across samples were grouped as “Others” in the bar plots. The genomic integrity of the TGFβ pathway was assessed by examining key regulatory genes, including SMAD2, SMAD4, ACVR2A, TGFBR1, and TGFBR2.

For neo-antigen detection, HLA-HD was used to determine the 6-digit class I HLA genotypes from quality-controlled FASTQ files.^46^ After verifying the absence of somatic mutations within class I HLA regions, NeoPredPipe was employed to predict binding affinities of potential 8-, 9-, and 10-mer neo-peptides, derived from nonsynonymous somatic mutations, using default settings.^47^

### Statistical analysis

The Simon two-stage design with the Optimal method was used to determine the sample size. The target ORR was set at 60%, with the null hypothesis representing an ORR of 40%.

Using a one-sided α-error of 5% and a power of 80%, Stage 1 required 16 evaluable patients. Progression to Stage 2 depended on observing at least 8 responses in Stage 1, leading to the enrollment of an additional 45 evaluable patients. The treatment was deemed active if at least 32 responders were observed across all 61 patients. If fewer than 32 responses were achieved, further development decisions were to be made collaboratively by the investigators and the sponsor.

Efficacy and safety analyses included all patients who received at least one dose of the study treatment. ORR and DCR were estimated with 95% confidence intervals (CIs) using the Wilson Score method. Kaplan-Meier estimates were used to calculate DoR, PFS, and OS, with 95% CIs determined using the Brookmeyer-Crowley method. All statistical analyses were performed using SAS® software (version 9.4, SAS Institute Inc., Cary, USA).

## Supplementary information

Supplementary material

Study protocol

## Data Availability

Deidentified individual participant data underlying the results reported in this article will be made available upon request, starting 24 months after the study’s completion. Researchers interested in accessing the data must submit a proposal to the corresponding author, detailing the purpose and justification for their request. The leading clinical site and study sponsor will review the proposal to ensure compliance with any applicable intellectual property or confidentiality obligations. Access to the data will require a signed data access agreement with the sponsor. The study protocol is included in the supplementary materials.

## References

[1] Aparicio, J. et al. Metastatic Colorectal Cancer. First Line Therapy for Unresectable Disease. *J Clin Med*. **9** (2020). 10.3390/jcm9123889 PMC7761096 33265959

[2] Atreya, C. E., Yaeger, R. & Chu, E. Systemic Therapy for Metastatic Colorectal Cancer: From Current Standards to Future Molecular Targeted Approaches. *Am Soc Clin Oncol Educ Book*. 246–256 (2017). 10.1200/EDBK_175679 28561718

[3] Wang, F. et al. Expert opinions on immunotherapy for patients with colorectal cancer. *Cancer Commun (Lond)* **40**, 467–472 (2020). 32945625 10.1002/cac2.12095 PMC7571394

[4] Overman, M. J. et al. Durable Clinical Benefit With Nivolumab Plus Ipilimumab in DNA Mismatch Repair-Deficient/Microsatellite Instability-High Metastatic Colorectal Cancer. *J Clin Oncol* **36**, 773–779 (2018). 29355075 10.1200/JCO.2017.76.9901

[5] Shahda, S. et al. A phase II study of pembrolizumab in combination with mFOLFOX6 for patients with advanced colorectal cancer. *J Clin Oncol* **35**, 3541–3541 (2017).

[6] Ghiringhelli, F. et al. Durvalumab and tremelimumab in combination with FOLFOX in patients with RAS-mutated, microsatellite-stable, previously untreated metastatic colorectal cancer (MCRC): Results of the first intermediate analysis of the phase Ib/II MEDETREME trial. *J Clin Oncol* **38**, 3006 (2020).

[7] Feng, J. et al. SHR-1701, a Bifunctional Fusion Protein Targeting PD-L1 and TGFβ, for Recurrent or Metastatic Cervical Cancer: A Clinical Expansion Cohort of a Phase I Study. *Clin Cancer Res* **28**, 5297–5305 (2022). 35653122 10.1158/1078-0432.CCR-22-0346

[8] Liu, D. et al. Bifunctional anti-PD-L1/TGF-βRII agent SHR-1701 in advanced solid tumors: a dose-escalation, dose-expansion, and clinical-expansion phase 1 trial. *BMC Med* **20**, 408 (2022). 36280870 10.1186/s12916-022-02605-9 PMC9594927

[9] Batlle, E. & Massagué, J. Transforming Growth Factor-β Signaling in Immunity and Cancer. *Immunity.* **50**, 924–940 (2019). 30995507 10.1016/j.immuni.2019.03.024 PMC7507121

[10] Mariathasan, S. et al. TGFβ attenuates tumour response to PD-L1 blockade by contributing to exclusion of T cells. *Nature.* **554**, 544–548 (2018). 29443960 10.1038/nature25501 PMC6028240

[11] Kim, B. G. et al. Novel therapies emerging in oncology to target the TGF-β pathway. *J Hematol Oncol* **14**, 55 (2021). 33823905 10.1186/s13045-021-01053-x PMC8022551

[12] Huang, C. Y. et al. Recent progress in TGF-β inhibitors for cancer therapy. *Biomed Pharmacother* **134**, 111046 (2021). 33341049 10.1016/j.biopha.2020.111046

[13] Wang, J. et al. Single-cell and bulk transcriptomics identifies a tumor-specific CD36+ cancer-associated fibroblast subpopulation in colorectal cancer. *Cancer Commun (Lond)* (2023). 10.1002/cac2.12506 PMC11024678 37990474

[14] Petljak, M. et al. Mechanisms of APOBEC3 mutagenesis in human cancer cells. *Nature.* **607**, 799–807 (2022). 35859169 10.1038/s41586-022-04972-y PMC9329121

[15] Zhao, Q. et al. Comprehensive profiling of 1015 patients' exomes reveals genomic-clinical associations in colorectal cancer. *Nat Commun* **13**, 2342 (2022). 35487942 10.1038/s41467-022-30062-8 PMC9055073

[16] Lenz, H. J. et al. Modified FOLFOX6 plus bevacizumab with and without nivolumab for first-line treatment of metastatic colorectal cancer: phase 2 results from the CheckMate 9X8 randomized clinical trial. *J Immunother Cancer*. **12** (2024). 10.1136/jitc-2023-008409 PMC10941175 38485190

[17] Antoniotti, C. et al. Upfront FOLFOXIRI plus bevacizumab with or without atezolizumab in the treatment of patients with metastatic colorectal cancer (AtezoTRIBE): a multicentre, open-label, randomised, controlled, phase 2 trial. *Lancet Oncol* **23**, 876–887 (2022). 35636444 10.1016/S1470-2045(22)00274-1

[18] Wang, F. et al. Combined anti-PD-1, HDAC inhibitor and anti-VEGF for MSS/pMMR colorectal cancer: a randomized phase 2 trial. *Nat Med* (2024). 10.1038/s41591-024-02813-1 38438735

[19] Conca, V. et al. Modified FOLFOXIRI plus cetuximab and avelumab as initial therapy in RAS wild-type unresectable metastatic colorectal cancer: Results of the phase II AVETRIC trial by GONO. *J Clin Oncol* **41**, 3575 (2023).

[20] Fang, X. et al. Sintilimab plus bevacizumab, oxaliplatin and capecitabine as first-line therapy in RAS-mutant, microsatellite stable, unresectable metastatic colorectal cancer: an open-label, single-arm, phase II trial. *EClinicalMedicine* **62**, 102123 (2023). 37554125 10.1016/j.eclinm.2023.102123 PMC10404864

[21] David, C. J. et al. TGF-β Tumor Suppression through a Lethal EMT. *Cell.* **164**, 1015–1030 (2016). 26898331 10.1016/j.cell.2016.01.009 PMC4801341

[22] David, C. J. & Massagué, J. Contextual determinants of TGFβ action in development, immunity and cancer. *Nat Rev Mol Cell Biol* **19**, 419–435 (2018). 29643418 10.1038/s41580-018-0007-0 PMC7457231

[23] Claps, G. et al. The multiple roles of LDH in cancer. *Nat Rev Clin Oncol* **19**, 749–762 (2022). 36207413 10.1038/s41571-022-00686-2

[24] Mezquita, L. et al. Association of the Lung Immune Prognostic Index With Immune Checkpoint Inhibitor Outcomes in Patients With Advanced Non-Small Cell Lung Cancer. *JAMA Oncol* **4**, 351–357 (2018). 29327044 10.1001/jamaoncol.2017.4771 PMC5885829

[25] Zhang, X. et al. Prognostic significance of serum LDH in small cell lung cancer: A systematic review with meta-analysis. *Cancer Biomark* **16**, 415–423 (2016). 27062698 10.3233/CBM-160580 PMC13016485

[26] Gibney, G. T., Weiner, L. M. & Atkins, M. B. Predictive biomarkers for checkpoint inhibitor-based immunotherapy. *Lancet Oncol* **17**, e542–e551 (2016). 27924752 10.1016/S1470-2045(16)30406-5 PMC5702534

[27] Wang, S., Jia, M., He, Z. & Liu, X. S. APOBEC3B and APOBEC mutational signature as potential predictive markers for immunotherapy response in non-small cell lung cancer. *Oncogene.* **37**, 3924–3936 (2018). 29695832 10.1038/s41388-018-0245-9 PMC6053356

[28] Saltz, L. B. et al. Bevacizumab in combination with oxaliplatin-based chemotherapy as first-line therapy in metastatic colorectal cancer: a randomized phase III study. *J Clin Oncol* **26**, 2013–2019 (2008). 18421054 10.1200/JCO.2007.14.9930

[29] Pan, Q. Z. et al. XELOX (capecitabine plus oxaliplatin) plus bevacizumab (anti-VEGF-A antibody) with or without adoptive cell immunotherapy in the treatment of patients with previously untreated metastatic colorectal cancer: a multicenter, open-label, randomized, controlled, phase 3 trial. *Signal Transduct Target Ther* **9**, 79 (2024). 38565886 10.1038/s41392-024-01788-2 PMC10987514

[30] Feng, J. et al. 1278P SHR-1701, a bifunctional fusion protein targeting PD-L1 and TGF-β, as first-line therapy for PD-L1+ advanced/metastatic NSCLC: Data from a clinical expansion cohort of a phase I study. *Ann Oncol* **32**, S995 (2021).

[31] Shi, M. et al. SHR-1701, a bifunctional fusion protein targeting PD-L1 and TGF-β, for advanced NSCLC with EGFR mutations: Data from a multicenter phase 1 study. *J Clin Oncol* **39**, 9055 (2021).

[32] Cammareri, P. et al. Inactivation of TGFβ receptors in stem cells drives cutaneous squamous cell carcinoma. *Nat Commun* **7**, 12493 (2016). 27558455 10.1038/ncomms12493 PMC5007296

[33] Lacouture, M. E. et al. Cutaneous keratoacanthomas/squamous cell carcinomas associated with neutralization of transforming growth factor β by the monoclonal antibody fresolimumab (GC1008). *Cancer Immunol Immunother* **64**, 437–446 (2015). 25579378 10.1007/s00262-015-1653-0 PMC6730642

[34] Rose, A. M., Sansom, O. J. & Inman, G. J. Loss of TGF-β signaling drives cSCC from skin stem cells - More evidence. *Cell Cycle* **16**, 386–387 (2017). 27860538 10.1080/15384101.2016.1259892 PMC5351926

[35] Gulley, J. et al. 1689P Adverse event management during treatment with bintrafusp alfa, a bifunctional fusion protein targeting TGF-β and PD-L1: Treatment guidelines based on experience in clinical trials. *Ann Oncol* **32**, S1181–S1182 (2021).

[36] Claeson, M. et al. Assessment of Incidence Rate and Risk Factors for Keratoacanthoma Among Residents of Queensland, Australia. *JAMA Dermatol* **156**, 1324–1332 (2020). 33026421 10.1001/jamadermatol.2020.4097 PMC7542522

[37] Green, A. C. & Olsen, C. M. Cutaneous squamous cell carcinoma: an epidemiological review. *Br J Dermatol* **177**, 373–381 (2017). 28211039 10.1111/bjd.15324

[38] Strauss, J. et al. Phase I Trial of M7824 (MSB0011359C), a Bifunctional Fusion Protein Targeting PD-L1 and TGFβ, in Advanced Solid Tumors. *Clin Cancer Res* **24**, 1287–1295 (2018). 29298798 10.1158/1078-0432.CCR-17-2653 PMC7985967

[39] Li, H. & Durbin, R. Fast and accurate short read alignment with Burrows-Wheeler transform. *Bioinformatics.* **25**, 1754–1760 (2009). 19451168 10.1093/bioinformatics/btp324 PMC2705234

[40] Danecek, P. et al. Twelve years of SAMtools and BCFtools. *Gigascience*. **10** (2021). 10.1093/gigascience/giab008 PMC7931819 33590861

[41] McKenna, A. et al. The Genome Analysis Toolkit: a MapReduce framework for analyzing next-generation DNA sequencing data. *Genome Res* **20**, 1297–1303 (2010). 20644199 10.1101/gr.107524.110 PMC2928508

[42] Talevich, E., Shain, A. H., Botton, T. & Bastian, B. C. CNVkit: Genome-Wide Copy Number Detection and Visualization from Targeted DNA Sequencing. *PLoS Comput Biol* **12**, e1004873 (2016). 27100738 10.1371/journal.pcbi.1004873 PMC4839673

[43] Wang, S., Tao, Z., Wu, T. & Liu, X. S. Sigflow: an automated and comprehensive pipeline for cancer genome mutational signature analysis. *Bioinformatics.* **37**, 1590–1592 (2021). 33270873 10.1093/bioinformatics/btaa895 PMC8275980

[44] Wang, S. et al. Copy number signature analysis tool and its application in prostate cancer reveals distinct mutational processes and clinical outcomes. *PLoS Genet* **17**, e1009557 (2021). 33945534 10.1371/journal.pgen.1009557 PMC8121287

[45] Forbes, S. A. et al. COSMIC: exploring the world's knowledge of somatic mutations in human cancer. *Nucleic Acids Res* **43**, D805–D811 (2015). 25355519 10.1093/nar/gku1075 PMC4383913

[46] Kawaguchi, S. & Matsuda, F. High-Definition Genomic Analysis of HLA Genes Via Comprehensive HLA Allele Genotyping. *Methods Mol Biol* **2131**, 31–38 (2020). 32162249 10.1007/978-1-0716-0389-5_3

[47] Schenck, R. O. et al. NeoPredPipe: high-throughput neoantigen prediction and recognition potential pipeline. *BMC Bioinformatics* **20**, 264 (2019). 31117948 10.1186/s12859-019-2876-4 PMC6532147

