# Supplementary material for: Addition of SHR-1701 to first-line capecitabine and oxaliplatin (XELOX) plus bevacizumab for unresectable metastatic colorectal cancer
Source: Signal Transduct Target Ther. 2024 Dec 16;9:349. doi: 10.1038/s41392-024-02063-0 (PMC11647033; doi:10.1038/s41392-024-02063-0)
Supplement: Supplementary file 1 — Supplementary material [file 41392_2024_2063_MOESM1_ESM.docx]

Supplementary Materials for

**Efficacy and safety of SHR-1701 in combination with bevacizumab and XELOX as first-line treatment for patients with unresectable metastatic colorectal cancer**

Miao-Zhen Qiu, Yuxian Bai, Jufeng Wang, Kangsheng Gu, Mudan Yang, Yifu He, Cheng Yi, Yongdong Jin, Bo Liu, Feng Wang, Yu-kun Chen, Wei Dai, Yingyi Jiang, Chuanpei Huang, Rui-Hua Xu, Hui-Yan Luo

**Correspondence to:**

Hui-Yan Luo (luohy@sysucc.org.cn), Rui-Hua Xu ([xurh@sysucc.org.cn](mailto:xurh@sysucc.org.cn)) and Miao-Zhen Qiu (qiumzh@sysucc.org.cn)

**This PDF file includes:**

Figure S1

Tables S1 to S8


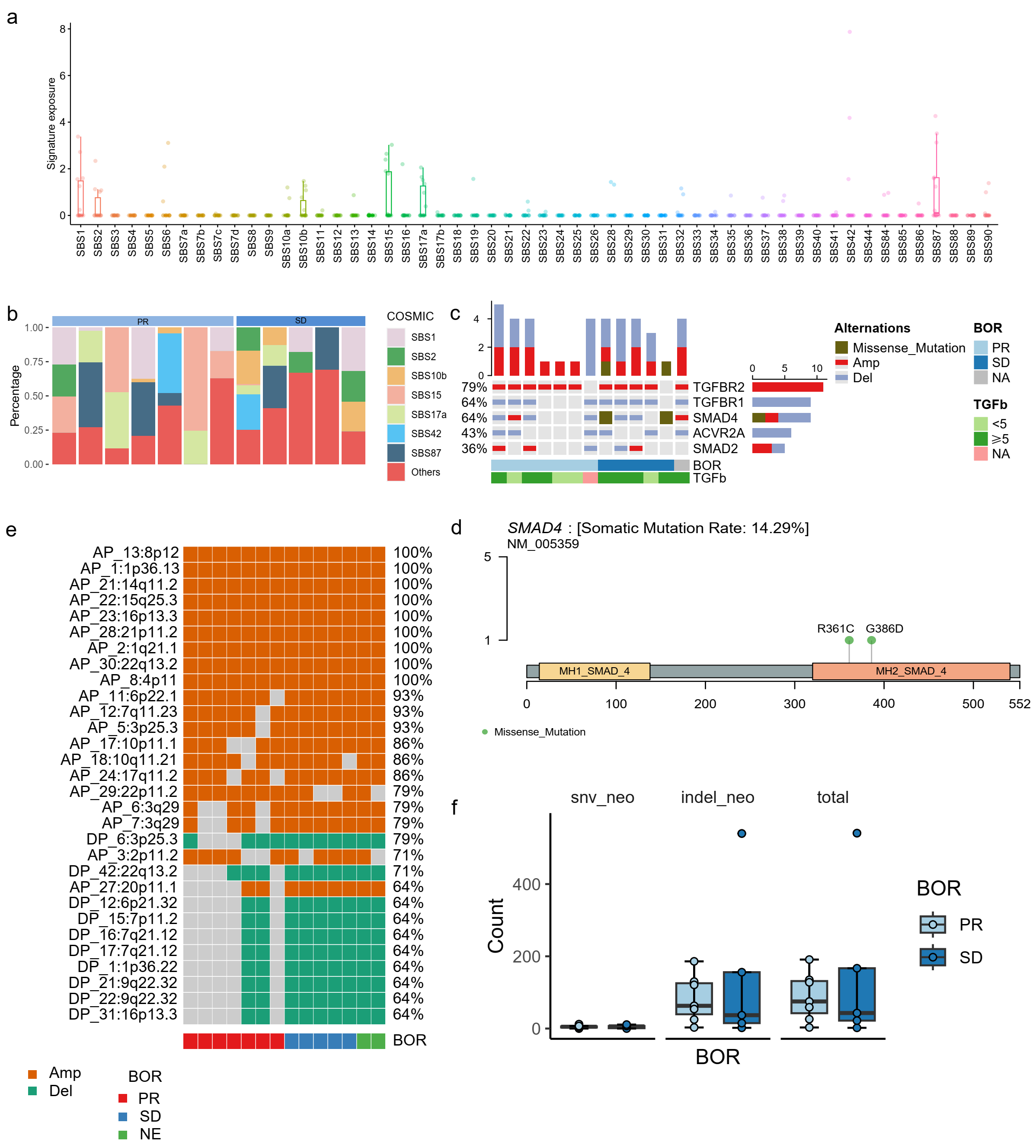


# Figure S1. Genomic characterization and mutational analysis of the 14 patients in the SHR-1701 cohort.

(A) Boxplot depicting the absolute exposure scores of COSMIC Single Base Substitution (SBS) signatures. (B) Proportion of SBS signature exposure across the 14 patients in the SHR-1701 cohort. (C) Genomic alteration landscape of the TGF-β pathway in the SHR-1701 cohort. (D) Detailed mutational sites of the *SMAD4* gene. (E) Somatic copy number alteration (SCNA) landscape of the SHR-1701 cohort, inferred using GISTIC 2.0. (F) Boxplot comparison of predicted neoantigen burden derived from single nucleotide variants (SNVs) and insertions/deletions (indels) between PR and stable SD groups.

SBS, single base substitution; GISTIC, Genomic Identification of Significant Targets in Cancer; SCNA, somatic copy number alteration; SNV, single nucleotide variant; PR, partial response; SD, stable disease.

# Table S1. Subsequent anticancer therapy after the end of study treatment

|  | **All patients (n=62)** |
| --- | --- |
| Any subsequent therapy | 24 (38.7) |
| Any anti-PD-1/PD-L1 therapy | 1 (1.6) |
| Any non-anti-PD-1/PD-L1 therapy | 24 (38.7) |
| Chemotherapy | 23 (37.1) |
| VEGF inhibitor | 15 (24.2) |
| EGFR inhibitor | 3 (4.8) |

Data are n (%).

PD-1, programmed death-1; PD-L1, programmed death-ligand 1; VEGF: vascular endothelial growth factor; EGFR: epidermal growth factor receptor.

# Table S2. Analysis of objective response rates and progression-free survival based on PD-L1 expression, *RAS/BRAF* gene status, primary tumor site and location, TGF-β1 expression, ,pSMAD2/3 expression, and LDH levels.

| **Variables** | **Subgroups** | **ORR, n (%) [95% CI]** | **PFS, median (95% CI), months** |
| --- | --- | --- | --- |
| PD-L1 expression^#^ | Positive (TPS ≥ 1% or CPS ≥ 1) (n=33) | 19 (57.6%) [40.8-72.8] | 13.9 (8.3-NR) |
|  | Negative (n=26) | 16 (61.5%) [42.5-77.6] | 9.3 (7.1-13.7) |
| *RAS* gene status | Wild-type (n=24) | 15 (62.5%) [42.7-78.8] | 14.0 (8.4-NR) |
|  | Mutant-type (n=36) | 21 (58.3%) [42.2-72.9] | 9.9 (6.7-13.7) |
| *BRAF* gene status | Wild-type (n=47) | 27 (57.4%) [43.3-70.5] | 11.3 (7.3, 13.9) |
|  | Mutant-type (n=4) | 3 (75.0%) [30.1-95.4] | NR (11.5, NR) |
| *RAS/BRAF* gene status | *RAS* and *BRAF* wild-type (n=11) | 7 (63.6%) [35.4-84.8] | NR (8.4-NR) |
|  | *RAS* mutant-type or *BRAF* mutant-type (n=40) | 24 (60.0%) [44.6-73.7] | 11.3 (7.1-13.9) |
| Primary tumor site | Right (n=22) | 14 (63.6%) [43.0-80.3] | 8.5 (4.7-11.5) |
|  | Left or rectum (n=40) | 23 (57.5%) [42.2-71.5] | 11.9 (8.4-15.0) |
| Location of primary tumor | Colon (n=40) | 26 (65.0%) [49.5-77.9] | 9.3 (7.1-11.9) |
|  | Rectum (n=22) | 11 (50.0%) [30.7-69.3] | 13.7 (7.3-NR) |
| TGF-β1 expression* | Negative (n=9) | 5 (55.6%) [26.7-81.1] | 7.1 (2.1-13.9) |
|  | Positive (n=10) | 4 (40.0%) [16.8-68.7] | 8.1 (3.2-8.9) |
| pSMAD 2/3 expression* | ≥ 80% (n=10) | 5 (50.0%) [23.7-76.3] | 8.5 (2.1-13.9) |
|  | < 80% (n=9) | 4 (44.4%) [18.9-73.3] | 7.3 (4.7-8.5) |
| LDH | < ULN (n=44) | 30 (68.2%) [53.4-80.0] | 11.9 (8.5-14.1) |
|  | > ULN (n=18) | 7 (38.9%) [20.3-61.4] | 7.1 (3.2-11.5) |

^#^ PD-L1 positive was defined as TPS ≥1% or CPS ≥1; PD-L1 negative was defined as any values other than TPS ≥ 1% or CPS ≥ 1 in cases with CPS or TPS detection.

^*^ Tumor TGF-β1 and pSMAD2/3 expressions were only detected in 19 patients.

Note: Prespecified subgroup analyses of ORR and PFS were conducted based on PD-L1 expression (positive vs. negative), *RAS* gene status (mutant vs. wild-type), primary tumor site (left colon or rectum vs. right colon), and primary lesion location (colon vs. rectum). Additionally, subgroup analyses of ORR and PFS were performed based on *BRAF* gene status (wild-type vs. mutant), combined *RAS/BRAF* gene status (*RAS* and *BRAF* wild-type vs. *RAS* mutant or *BRAF* mutant), TGF-β1 expression (negative vs. positive), pSMAD2/3 expression (≥ 80% vs. < 80%), and LDH levels (< ULN vs. > ULN) as post hoc analyses.

PD-L1, programmed death-ligand 1; ORR, objective response rate; PFS, progression-free survival; CI, confidence interval; TPS, tumor proportion score; CPS, combined positive score; NR, not reached; ULN, upper limit of normal.

# Table S3. Demographics and baseline characteristics of genome biomarker evaluable population (BEP)

|  | **All patients (n=14)** |
| --- | --- |
| Age, years, median (range) | 46 (23-66) |
| Sex, n (%) |  |
| Male | 8 (57.1) |
| Female | 6 (42.9) |
| BMI, kg/m^2^, mean ± SD | 22.3 ± 7.1 |
| Location of primary tumor, n (%) |  |
| Colon | 9 (64.3) |
| Rectum | 5 (35.7) |
| Primary tumor site, n (%) |  |
| Left or rectum | 9 (64.3) |
| Right | 5 (35.7) |
| MSS/pMMR, n (%) | 14 (100) |
| Surgery on primary tumor, n (%) |  |
| Radical surgery | 8 (57.1) |
| Palliative surgery | 4 (28.6) |
| Previous (neo) adjuvant therapy, n (%) | 2 (14.3) |
| ECOG performance status, n (%) |  |
| 0 | 14 (100) |
| Metastasis sites, n (%) |  |
| Liver | 12 (85.7) |
| Peritoneum | 9 (64.3) |
| *RAS* gene status, n (%) ^a^ |  |
| Wild-type | 3 (21.4) |
| Mutant-type | 11 (78.6) |
| *BRAF* gene status, n (%) ^a^ |  |
| Wild-type | 11 (78.6) |
| Unknown | 3 (21.4) |
| *RAS*/*BRAF*, n (%) ^a^ |  |
| *RAS* and *BRAF* wild-type | 1 (7.1) |
| *RAS* mutant-type or *BRAF* mutant-type | 11 (78.6) |
| PD-L1 expression, n (%) ^a b^ |  |
| Positive (TPS ≥ 1% or CPS ≥ 1) | 10 (71.4) |
| Negative | 4 (28.6) |
| TGF-β1, n (%) ^a^ |  |
| Positive | 8 (57.1) |
| Negative | 5 (35.7) |
| pSMAD 2/3 expression, n (%) ^a^ |  |
| ≥ 80% | 7 (50.0) |
| < 80% | 6 (42.9) |

^a^ The status was not detected in remaining patients.

^b^ PD-L1 positive was defined as TPS ≥ 1% or CPS ≥ 1; PD-L1 negative was defined as any values other than TPS ≥ 1% or CPS ≥ 1 in cases with CPS or TPS detection.

BEP, biomarker evaluable population; BMI, body mass index; SD, standard deviation; MSS, microsatellite stable; pMMR, mismatch repair-proficient; ECOG, Eastern Cooperative Oncology Group; PD-L1, programmed death-ligand 1; TPS, tumor proportion score; CPS, combined positive score.

# Table S4. TRAEs leading to discontinuation of the study treatment

|  | **All patients (n=62)** | |
| --- | --- | --- |
|  | **Any grade** | **Grade ≥ 3** |
| TRAEs leading to any treatment discontinuation | 13 (21.0) | 7 (11.3) |
| Anal hemorrhage | 1 (1.6) | 1 (1.6) |
| Immune-mediated enterocolitis | 1 (1.6) | 1 (1.6) |
| Lower gastrointestinal hemorrhage | 1 (1.6) | 1 (1.6) |
| Bilirubin conjugated increased | 1 (1.6) | 1 (1.6) |
| Blood bilirubin increased | 1 (1.6) | 1 (1.6) |
| Blood bilirubin unconjugated increased | 1 (1.6) | 1 (1.6) |
| Hypopituitarism | 1 (1.6) | 1 (1.6) |
| Immune-mediated hypophysitis | 1 (1.6) | 1 (1.6) |
| Infusion related reaction | 1 (1.6) | 1 (1.6) |
| Blood creatinine increased | 1 (1.6) | 0 |
| Platelet count decreased | 1 (1.6) | 0 |
| Anemia | 1 (1.6) | 0 |
| Palpitations | 1 (1.6) | 0 |
| Skin infection | 1 (1.6) | 0 |
| Neurotoxicity | 1 (1.6) | 0 |
| Proteinuria | 1 (1.6) | 0 |
| Immune-mediated dermatitis | 1 (1.6) | 0 |
| Venous thrombosis limb | 1 (1.6) | 0 |

Data are presented as n (%).

TRAEs, treatment-related adverse events.

# Table S5. Immune-related adverse events regardless of attribution to study treatment.

|  | **All patients (n=62)** | |
| --- | --- | --- |
|  | **Any grade** | **Grade ≥ 3** |
| Any irAEs | 21 (33.9) | 9 (14.5) |
| Hypothyroidism | 10 (16.1) | 0 |
| Hyperthyroidism | 7 (11.3) | 0 |
| Aspartate aminotransferase increased | 3 (4.8) | 0 |
| Blood thyroid stimulating hormone decreased | 3 (4.8) | 0 |
| Gamma-glutamyltransferase increased | 2 (3.2) | 2 (3.2) |
| Alanine aminotransferase increased | 2 (3.2) | 0 |
| Blood alkaline phosphatase increased | 2 (3.2) | 0 |
| Pruritus | 2 (3.2) | 0 |
| Thyroxine free increased | 2 (3.2) | 0 |
| Tri-iodothyronine free increased | 2 (3.2) | 0 |
| Amylase increased | 1 (1.6) | 0 |
| Bilirubin conjugated increased | 1 (1.6) | 1 (1.6) |
| Blood bilirubin increased | 1 (1.6) | 1 (1.6) |
| Blood bilirubin unconjugated increased | 1 (1.6) | 1 (1.6) |
| Hypopituitarism | 1 (1.6) | 1 (1.6) |
| Immune-mediated dermatitis | 1 (1.6) | 1 (1.6) |
| Immune-mediated enterocolitis | 1 (1.6) | 1 (1.6) |
| Immune-mediated hypophysitis | 1 (1.6) | 1 (1.6) |
| Infusion related reaction | 1 (1.6) | 1 (1.6) |
| Intestinal obstruction | 1 (1.6) | 1 (1.6) |
| Multiple organ dysfunction syndrome | 1 (1.6) | 1 (1.6) |
| Sepsis | 1 (1.6) | 1 (1.6) |
| Septic shock | 1 (1.6) | 1 (1.6) |
| Blood prolactin increased | 1 (1.6) | 0 |
| Blood thyroid stimulating hormone increased | 1 (1.6) | 0 |
| Colitis ulcerative | 1 (1.6) | 0 |
| Constipation | 1 (1.6) | 0 |
| Decreased appetite | 1 (1.6) | 0 |
| Dermatitis | 1 (1.6) | 0 |
| Diarrhea | 1 (1.6) | 0 |
| Hemoptysis | 1 (1.6) | 0 |
| Hyperglycemia | 1 (1.6) | 0 |
| Hypertriglyceridemia | 1 (1.6) | 0 |
| Hypoaesthesia | 1 (1.6) | 0 |
| Immune-mediated hypothyroidism | 1 (1.6) | 0 |
| Immune-mediated lung disease | 1 (1.6) | 0 |
| Lipase increased | 1 (1.6) | 0 |
| Platelet count decreased | 1 (1.6) | 0 |
| Protein urine present | 1 (1.6) | 0 |
| Rash | 1 (1.6) | 0 |
| Renal impairment | 1 (1.6) | 0 |
| Skin infection | 1 (1.6) | 0 |
| Stomatitis | 1 (1.6) | 0 |
| Thyroglobulin increased | 1 (1.6) | 0 |
| Thyroxine increased | 1 (1.6) | 0 |
| Tri-iodothyronine increased | 1 (1.6) | 0 |

Data are presented as n (%).

irAE, immune-related adverse event.

# Table S6. Treatment-related SAEs

|  | **All patients (n=62)** | |
| --- | --- | --- |
|  | **Any grade** | **Grade ≥ 3** |
| Any treatment-related SAEs | 19 (30.6) | 17 (27.4) |
| Pneumonia | 2 (3.2) | 2 (3.2) |
| Anal hemorrhage | 1 (1.6) | 1 (1.6) |
| Immune-mediated enterocolitis | 1 (1.6) | 1 (1.6) |
| Intestinal obstruction | 1 (1.6) | 1 (1.6) |
| Nausea | 1 (1.6) | 1 (1.6) |
| Small intestinal obstruction | 1 (1.6) | 1 (1.6) |
| Upper gastrointestinal hemorrhage | 1 (1.6) | 1 (1.6) |
| Gastrointestinal infection | 1 (1.6) | 1 (1.6) |
| Sepsis | 1 (1.6) | 1 (1.6) |
| Septic shock | 1 (1.6) | 1 (1.6) |
| Death | 1 (1.6) | 1 (1.6) |
| Fatigue | 1 (1.6) | 1 (1.6) |
| Multiple organ dysfunction syndrome | 1 (1.6) | 1 (1.6) |
| Hypopituitarism | 1 (1.6) | 1 (1.6) |
| Immune-mediated hypophysitis | 1 (1.6) | 1 (1.6) |
| Alanine aminotransferase increased | 1 (1.6) | 1 (1.6) |
| Blood creatinine increased | 1 (1.6) | 1 (1.6) |
| Infusion related reaction | 1 (1.6) | 1 (1.6) |
| Hypokalaemia | 1 (1.6) | 1 (1.6) |
| Cerebral hemorrhage | 1 (1.6) | 1 (1.6) |
| Renal failure | 1 (1.6) | 1 (1.6) |
| Abnormal uterine bleeding | 1 (1.6) | 1 (1.6) |
| Hypovolemic shock | 1 (1.6) | 1 (1.6) |
| Lower gastrointestinal hemorrhage | 1 (1.6) | 0 |
| Mouth hemorrhage | 1 (1.6) | 0 |
| Epistaxis | 1 (1.6) | 0 |
| Immune-mediated lung disease | 1 (1.6) | 0 |
| Ocular hyperaemia | 1 (1.6) | 0 |

Data are n (%).

SAE, serious adverse event.

# Table S7. Study sites and principal investigators

| **Study center** | **Principle investigator** | **No. of patients enrolled** |
| --- | --- | --- |
| Department of Medical Oncology, Sun Yat-sen University Cancer Center, Guangzhou, China | Ruihua Xu | 24 |
| Department of Gastroenterology 1, Harbin Medical University Cancer Hospital, Harbin, China | Yuxian Bai | 11 |
| Medical Oncology, Henan Cancer Hospital/Affiliated Cancer Hospital of Zhengzhou University, Zhengzhou, China | Jufeng Wang | 6 |
| Oncology Ward 1, The First Affiliated Hospital of Anhui Medical University, Hefei, China | Kangsheng Gu | 5 |
| Gastroenterology Ward (2), Shanxi Provincial Cancer Hospital, Taiyuan, China | Mudan Yang | 4 |
| Medical Oncology Ward 1, Anhui Provincial Cancer Hospital, Hefei, China | Yifu He | 4 |
| Abdominal Oncology, West China School of Medicine/West China Hospital of Sichuan University, Chengdu, China | Cheng Yi | 3 |
| Sichuan Cancer Hospital & Institute, Sichuan Cancer Center, University of Electronic Science and Technology of China, Medical Oncology, Chengdu, China | Yongdong Jin | 2 |
| Medical Oncology, Cancer Hospital Affiliated to Shandong First Medical University, Zhenzhou, China | Bo Liu | 2 |
| Oncology Department 1, The First Affiliated Hospital of Zhengzhou University, Zhengzhou, China | Feng Wang | 1 |

# Table S8. Target gene list and the synonymous genes conversions

| **Gene list** | **Latest name** | **Gene list** | **Latest name** | **Gene list** | **Latest name** |
| --- | --- | --- | --- | --- | --- |
| ABCB1 | ABCB1 | BLM | BLM | CSF1R | CSF1R |
| ABCG2 | ABCG2 | BMPR1A | BMPR1A | CSF3R | CSF3R |
| ABL1 | ABL1 | BRAF | BRAF | CTLA4 | CTLA4 |
| ABL2 | ABL2 | BRCA1 | BRCA1 | CTNNB1 | CTNNB1 |
| ADAR | ADAR | BRCA2 | BRCA2 | CXCR4 | CXCR4 |
| AKT1 | AKT1 | BRD4 | BRD4 | DCUN1D1 | DCUN1D1 |
| AKT2 | AKT2 | BRIP1 | BRIP1 | DDR1 | DDR1 |
| AKT3 | AKT3 | BTG1 | BTG1 | DDR2 | DDR2 |
| ALK | ALK | BTK | BTK | DDX41 | DDX41 |
| ALOX12B | ALOX12B | C11orf30 | EMSY | DHX15 | DHX15 |
| AMER1 | AMER1 | CALR | CALR | DICER1 | DICER1 |
| APC | APC | CBL | CBL | DNAJB1 | DNAJB1 |
| APOBEC3A | APOBEC3A | CCND1 | CCND1 | DNMT1 | DNMT1 |
| APOBEC3B | APOBEC3B | CCND2 | CCND2 | DNMT3A | DNMT3A |
| AR | AR | CCND3 | CCND3 | DNMT3B | DNMT3B |
| ARAF | ARAF | CCNE1 | CCNE1 | DOT1L | DOT1L |
| ARID1A | ARID1A | CD19 | CD19 | DUSP4 | DUSP4 |
| ARID1B | ARID1B | CD274 | CD274 | EED | EED |
| ARID2 | ARID2 | CD276 | CD276 | EGFL7 | EGFL7 |
| ARID5B | ARID5B | CD44 | CD44 | EGFR | EGFR |
| ATM | ATM | CD74 | CD74 | EIF1AX | EIF1AX |
| ATR | ATR | CD79A | CD79A | EP300 | EP300 |
| ATRX | ATRX | CD79B | CD79B | EPAS1 | EPAS1 |
| AURKA | AURKA | CDC42 | CDC42 | EPCAM | EPCAM |
| AURKB | AURKB | CDH1 | CDH1 | EPHA2 | EPHA2 |
| AXIN1 | AXIN1 | CDK12 | CDK12 | EPHA3 | EPHA3 |
| AXIN2 | AXIN2 | CDK4 | CDK4 | EPHA5 | EPHA5 |
| AXL | AXL | CDK6 | CDK6 | ERAP1 | ERAP1 |
| B2M | B2M | CDK8 | CDK8 | ERBB2 | ERBB2 |
| BACH1 | BACH1 | CDKN1A | CDKN1A | ERBB3 | ERBB3 |
| BAP1 | BAP1 | CDKN1B | CDKN1B | ERBB4 | ERBB4 |
| BARD1 | BARD1 | CDKN2A | CDKN2A | ERCC1 | ERCC1 |
| BBC3 | BBC3 | CDKN2B | CDKN2B | ERCC2 | ERCC2 |
| BCL10 | BCL10 | CDKN2C | CDKN2C | ERCC3 | ERCC3 |
| BCL2 | BCL2 | CENPA | CENPA | ERCC4 | ERCC4 |
| BCL2L1 | BCL2L1 | CHD1 | CHD1 | ERCC5 | ERCC5 |
| BCL2L11 | BCL2L11 | CHEK1 | CHEK1 | ERG | ERG |
| BCL2L2 | BCL2L2 | CHEK2 | CHEK2 | ERRFI1 | ERRFI1 |
| BCL6 | BCL6 | CIC | CIC | ESR1 | ESR1 |
| BCORL1 | BCORL1 | CLDN18 | CLDN18 | ESR2 | ESR2 |
| BCR | BCR | CRKL | CRKL | ETV4 | ETV4 |
| ETV6 | ETV6 | HDAC1 | HDAC1 | KDM6A | KDM6A |
| EWSR1 | EWSR1 | HDAC2 | HDAC2 | KDR | KDR |
| EZH2 | EZH2 | HGF | HGF | KEAP1 | KEAP1 |
| FAM175A | ABRAXAS1 | HLA-A | HLA-A | KIT | KIT |
| FAM46C | TENT5C | HLA-B | HLA-B | KLF4 | KLF4 |
| FAM58A | CCNQ | HLA-C | HLA-C | KMT2A | KMT2A |
| FANCA | FANCA | HLA-DPA1 | HLA-DPA1 | KMT2B | KMT2B |
| FANCC | FANCC | HLA-DPB1 | HLA-DPB1 | KMT2C | KMT2C |
| FANCD2 | FANCD2 | HLA-DQA1 | HLA-DQA1 | KMT2D | KMT2D |
| FANCE | FANCE | HLA-DQB1 | HLA-DQB1 | KRAS | KRAS |
| FANCF | FANCF | HLA-DRB1 | HLA-DRB1 | LATS1 | LATS1 |
| FANCG | FANCG | HLA-DRB3 | HLA-DRB3 | LATS2 | LATS2 |
| FANCI | FANCI | HLA-DRB4 | HLA-DRB4 | LMO1 | LMO1 |
| FANCL | FANCL | HLA-DRB5 | HLA-DRB5 | LRP1B | LRP1B |
| FAT1 | FAT1 | HOXB13 | HOXB13 | LYN | LYN |
| FBXO31 | FBXO31 | HRAS | HRAS | MAF | MAF |
| FBXW7 | FBXW7 | HSP90AA1 | HSP90AA1 | MALT1 | MALT1 |
| FGF19 | FGF19 | ICOSLG | ICOSLG | MAP2K1 | MAP2K1 |
| FGFR1 | FGFR1 | IDH1 | IDH1 | MAP2K2 | MAP2K2 |
| FGFR2 | FGFR2 | IDH2 | IDH2 | MAP2K4 | MAP2K4 |
| FGFR3 | FGFR3 | IFNG | IFNG | MAP3K1 | MAP3K1 |
| FGFR4 | FGFR4 | IFNGR1 | IFNGR1 | MAP3K7 | MAP3K7 |
| FH | FH | IFNGR2 | IFNGR2 | MAPK1 | MAPK1 |
| FLCN | FLCN | IGF1 | IGF1 | MAPK3 | MAPK3 |
| FLT1 | FLT1 | IGF1R | IGF1R | MCL1 | MCL1 |
| FLT3 | FLT3 | IGF2 | IGF2 | MDC1 | MDC1 |
| FLT4 | FLT4 | IKBKE | IKBKE | MDM2 | MDM2 |
| FOXA1 | FOXA1 | IKZF1 | IKZF1 | MDM4 | MDM4 |
| FOXO1 | FOXO1 | IL10 | IL10 | MED12 | MED12 |
| FOXP1 | FOXP1 | IL6 | IL6 | MEN1 | MEN1 |
| FYN | FYN | INHBA | INHBA | MET | MET |
| GATA1 | GATA1 | INSR | INSR | MITF | MITF |
| GATA2 | GATA2 | IRF1 | IRF1 | MKNK1 | MKNK1 |
| GATA3 | GATA3 | IRF2 | IRF2 | MLH1 | MLH1 |
| GATA4 | GATA4 | IRF4 | IRF4 | MLH3 | MLH3 |
| GATA6 | GATA6 | IRS1 | IRS1 | MPL | MPL |
| GEN1 | GEN1 | IRS2 | IRS2 | MRE11A | MRE11 |
| GLI1 | GLI1 | JAK1 | JAK1 | MSH2 | MSH2 |
| GNA11 | GNA11 | JAK2 | JAK2 | MSH3 | MSH3 |
| GNA13 | GNA13 | JAK3 | JAK3 | MSH6 | MSH6 |
| GNAQ | GNAQ | KAT6A | KAT6A | MST1 | MST1 |
| GNAS | GNAS | KDM5A | KDM5A | MST1R | MST1R |
| GSK3B | GSK3B | KDM5C | KDM5C | MTAP | MTAP |
| MTOR | MTOR | PIK3C2B | PIK3C2B | REL | REL |
| MUTYH | MUTYH | PIK3C2G | PIK3C2G | RET | RET |
| MYC | MYC | PIK3C3 | PIK3C3 | RFWD2 | COP1 |
| MYCN | MYCN | PIK3CA | PIK3CA | RHEB | RHEB |
| MYD88 | MYD88 | PIK3CB | PIK3CB | RHOA | RHOA |
| NBN | NBN | PIK3CD | PIK3CD | RICTOR | RICTOR |
| NCOA3 | NCOA3 | PIK3R1 | PIK3R1 | RIT1 | RIT1 |
| NCOR1 | NCOR1 | PIK3R3 | PIK3R3 | RNF43 | RNF43 |
| NF1 | NF1 | PIM1 | PIM1 | ROS1 | ROS1 |
| NF2 | NF2 | PLCG2 | PLCG2 | RPS6KA4 | RPS6KA4 |
| NFE2L2 | NFE2L2 | PLK2 | PLK2 | RPS6KB2 | RPS6KB2 |
| NKX2-1 | NKX2-1 | PMAIP1 | PMAIP1 | RPTOR | RPTOR |
| NOTCH1 | NOTCH1 | PMS1 | PMS1 | RSPO2 | RSPO2 |
| NOTCH2 | NOTCH2 | PMS2 | PMS2 | RTEL1 | RTEL1 |
| NOTCH3 | NOTCH3 | POLD1 | POLD1 | RUNX1 | RUNX1 |
| NOTCH4 | NOTCH4 | POLE | POLE | RYBP | RYBP |
| NPM1 | NPM1 | PPARG | PPARG | SDHA | SDHA |
| NRAS | NRAS | PPM1D | PPM1D | SDHB | SDHB |
| NRG1 | NRG1 | PPP2R1A | PPP2R1A | SDHC | SDHC |
| NSD1 | NSD1 | PPP2R2A | PPP2R2A | SDHD | SDHD |
| NT5C2 | NT5C2 | PRDM1 | PRDM1 | SERPINB3 | SERPINB3 |
| NTRK1 | NTRK1 | PREX2 | PREX2 | SERPINB4 | SERPINB4 |
| NTRK2 | NTRK2 | PRKCI | PRKCI | SETD1A | SETD1A |
| NTRK3 | NTRK3 | PRKD1 | PRKD1 | SETD2 | SETD2 |
| NUF2 | NUF2 | PRKDC | PRKDC | SF3B1 | SF3B1 |
| P2RY8 | P2RY8 | PSMB8 | PSMB8 | SGK1 | SGK1 |
| PAK1 | PAK1 | PTCH1 | PTCH1 | SLX4 | SLX4 |
| PALB2 | PALB2 | PTEN | PTEN | SMAD2 | SMAD2 |
| PARP1 | PARP1 | PTPN11 | PTPN11 | SMAD3 | SMAD3 |
| PARP2 | PARP2 | RAC1 | RAC1 | SMAD4 | SMAD4 |
| PARP3 | PARP3 | RAD21 | RAD21 | SMARCA4 | SMARCA4 |
| PBRM1 | PBRM1 | RAD50 | RAD50 | SMARCB1 | SMARCB1 |
| PDCD1 | PDCD1 | RAD51 | RAD51 | SMO | SMO |
| PDCD1LG2 | PDCD1LG2 | RAD51B | RAD51B | SOCS1 | SOCS1 |
| PDGFB | PDGFB | RAD51C | RAD51C | SOS1 | SOS1 |
| PDGFRA | PDGFRA | RAD51D | RAD51D | SOX17 | SOX17 |
| PDGFRB | PDGFRB | RAD52 | RAD52 | SOX2 | SOX2 |
| PDK1 | PDK1 | RAD54L | RAD54L | SOX9 | SOX9 |
| PDPK1 | PDPK1 | RAF1 | RAF1 | SPEN | SPEN |
| PGR | PGR | RARA | RARA | SRSF2 | SRSF2 |
| PIAS4 | PIAS4 | RASA1 | RASA1 | SSTR2 | SSTR2 |
| SPOP | SPOP | RB1 | RB1 | STAG1 | STAG1 |
| SRC | SRC | RECQL4 | RECQL4 | STAG2 | STAG2 |
| STAT5A | STAT5A |  |  |  |  |
| STAT5B | STAT5B |  |  |  |  |
| STK11 | STK11 |  |  |  |  |
| SUZ12 | SUZ12 |  |  |  |  |
| SYK | SYK |  |  |  |  |
| TAP1 | TAP1 |  |  |  |  |
| TAP2 | TAP2 |  |  |  |  |
| TCEB1 | ELOC |  |  |  |  |
| TERT | TERT |  |  |  |  |
| TET1 | TET1 |  |  |  |  |
| TET2 | TET2 |  |  |  |  |
| TGFBR1 | TGFBR1 |  |  |  |  |
| TGFBR2 | TGFBR2 |  |  |  |  |
| TMPRSS2 | TMPRSS2 |  |  |  |  |
| TNFAIP3 | TNFAIP3 |  |  |  |  |
| TNFRSF14 | TNFRSF14 |  |  |  |  |
| TOP1 | TOP1 |  |  |  |  |
| TOP2A | TOP2A |  |  |  |  |
| TP53 | TP53 |  |  |  |  |
| TRAF2 | TRAF2 |  |  |  |  |
| TSC1 | TSC1 |  |  |  |  |
| TSC2 | TSC2 |  |  |  |  |
| TYRO3 | TYRO3 |  |  |  |  |
| U2AF1 | U2AF1 |  |  |  |  |
| VEGFA | VEGFA |  |  |  |  |
| VHL | VHL |  |  |  |  |
| VTCN1 | VTCN1 |  |  |  |  |
| WT1 | WT1 |  |  |  |  |
| WWTR1 | WWTR1 |  |  |  |  |
| XIAP | XIAP |  |  |  |  |
| XPO1 | XPO1 |  |  |  |  |
| XRCC2 | XRCC2 |  |  |  |  |
| XRCC3 | XRCC3 |  |  |  |  |
| XRCC4 | XRCC4 |  |  |  |  |
| YAP1 | YAP1 |  |  |  |  |
| YES1 | YES1 |  |  |  |  |
| ZNF703 | ZNF703 |  |  |  |  |
