# Supplementary material for: Addition of SHR-1701 to first-line capecitabine and oxaliplatin (XELOX) plus bevacizumab for unresectable metastatic colorectal cancer
Source: Signal Transduct Target Ther. 2024 Dec 16;9:349. doi: 10.1038/s41392-024-02063-0 (PMC11647033; doi:10.1038/s41392-024-02063-0)
Supplement: Supplementary file 2 — Study protocol [file 41392_2024_2063_MOESM2_ESM.docx]

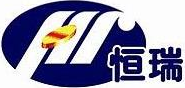


**A Randomized, Double-blind, Placebo-controlled, Multicenter Phase 2/3 Clinical Study of SHR-1701 or Placebo Combined with BP102 (Bevacizumab Biosimilar) and XELOX for First-line Treatment of Advanced Colorectal Cancer**

Protocol No.: SHR-1701-III-301

Study Phase: Phase 2/3

Compound No.: SHR-1701, BP102

Compound Name:

Study Director: Yang Qing

Leading site of clinical study: Sun Yet-sen University Cancer Center

Principal investigator: Rui-Hua Xu, professor

Version number: 3.0

Version Date: November 5, 2021

**Sponsor:** Jiangsu Hengrui Pharmaceutical Co., Ltd.

No.38, Huanghe Road, Lianyungang Economic and Technological Development Zone, Jiangsu Province, 222047

| **Confidential Statement**  The information contained in this protocol is confidential and only available for clinical investigators, which shall not be disclosed unless required by current laws or regulations. Its copyright is owned by Jiangsu Hengrui Pharmaceutical Co., Ltd. or its subsidiaries. The confidential information shall not be copied or distributed to any person not involved in this clinical study, except to those who have signed a confidentiality agreement with Jiangsu Hengrui Pharmaceutical Co., Ltd. or its subsidiaries. |
| --- |

**Protocol Signature Page for the Sponsor**

I have read and confirmed this clinical study protocol (Protocol No.: SHR-1701-III-301, Version No.: 3.0, Version Date: November 05, 2021). I agree to perform my duties in accordance with the laws of China, the Declaration of Helsinki, ICH-GCP, and this protocol.

**Sponsor:** Jiangsu Hengrui Pharmaceutical Co., Ltd.

| Yang Qing |  |  |
| --- | --- | --- |
| Senior Medical Director (Printed name) | Senior Medical Director (Signature) | Signature Date (MM/DD/YY) |

**Protocol Signature Page for the Principal Investigator (Leading Site)**

Protocol title: A Randomized, Double-blind, Placebo-controlled, Multicenter Phase 2/3 Clinical Study of SHR-1701 or Placebo Combined with BP102 (Bevacizumab Biosimilar) and XELOX for First-line Treatment of Advanced Colorectal Cancer

Protocol No.: SHR-1701-III-301

Version No and Date: version 3.0, November 5, 2021

By signing this protocol signature page, the investigator acknowledges and agrees that:

I have read this study protocol and its attachments.

I have fully discussed the contents of this protocol with the sponsor Jiangsu Hengrui Pharmaceutical Co., Ltd.

I agree to conduct the study in accordance with this protocol and to perform my duties in accordance with ICH-GCP, local regulations, and other applicable regulations.

This document contains confidential information, which shall not be disclosed without written authorization from the sponsor, except to persons directly involved in the conduct of the study or ethical/regulatory review.

I agree to ensure that all staff involved in this study have understood their obligations in fulfilling the above commitments.

**Testing facility:** ______________________________

| Rui-Hua Xu, professor |  |  |
| --- | --- | --- |
| Principal Investigator (Printed name) | Principal Investigator (Signature) | Signature Date (MM/DD/YY) |

**Protocol Signature Page of Principal Investigator (Participating Site)**

Protocol title: A Randomized, Double-blind, Placebo-controlled, Multicenter Phase 2/3 Clinical Study of SHR-1701 or Placebo Combined with BP102 (Bevacizumab Biosimilar) and XELOX for First-line Treatment of Advanced Colorectal Cancer

Protocol No.: SHR-1701-III-301

Version No and Date: version 3.0, November 5, 2021

By signing this protocol signature page, the investigator acknowledges and agrees that:

I have read this study protocol and its attachments.

I have fully discussed the contents of this protocol with the sponsor Jiangsu Hengrui Pharmaceutical Co., Ltd.

I agree to conduct the study in accordance with this protocol and to perform my duties in accordance with ICH-GCP, local regulations, and other applicable regulations.

This document contains confidential information, which shall not be disclosed without written authorization from the sponsor, except to persons directly involved in the conduct of the study or ethical/regulatory review.

I agree to ensure that all staff involved in this study have understood their obligations in fulfilling the above commitments.

**Testing facility:** __________________________________________

|  |  |  |
| --- | --- | --- |
| Principal Investigator (Printed name) | Principal Investigator (Signature) | Signature Date (MM/DD/YY) |

Synopsis

| **Study Title** | A Randomized, Double-blind, Placebo-controlled, Multicenter Phase 2/3 Clinical Study of SHR-1701 or Placebo Combined with BP102 (Bevacizumab Biosimilar) and XELOX for First-line Treatment of Advanced Colorectal Cancer |
| --- | --- |
| **Protocol No.** | SHR-1701-III-301 |
| **Version No. and Date** | Version 3.0, November 5, 2021 |
| **Study Phase** | II/III |
| **Investigational Drug** | **SHR-1701 injection**  (Manufacturer: Suzhou Suncadia Biopharmaceuticals Co., Ltd.)  **Placebo**  (Manufacturer: Suzhou Suncadia Biopharmaceuticals Co., Ltd.)  **BP102 injection**  (Manufacturer: Suzhou Suncadia Biopharmaceuticals Co., Ltd.)  **Oxaliplatin**  (Manufacturer: Jiangsu Hengrui Pharmaceutical Co., Ltd.)  **Capecitabine**  (Manufacturer: Jiangsu Hengrui Pharmaceutical Co., Ltd.) |
| **Sponsor** | Jiangsu Hengrui Pharmaceutical Co., Ltd. |
| **Principal Investigator** | Rui-Hua Xu, professor |
| **Participating Sites** | Approximately 50 sites |
| **Objectives** | **Phase 2 Study**  **Primary objectives:**   - To evaluate the safety of SHR-1701 combined with BP102 and XELOX for the first-line treatment of patients with advanced colorectal cancer; - To evaluate the objective response rate (ORR, evaluated by the investigator) achieved with SHR-1701 combined with BP102 and XELOX for the first-line treatment of patients with advanced colorectal cancer;   **Secondary objectives:**   - To evaluate the disease control rate (DCR), progression-free survival (PFS), duration of response (DoR) and overall survival (OS) achieved with SHR-1701 combined with BP102 and XELOX for the first-line treatment of patients with advanced colorectal cancer;   **Exploratory objective:**   - To explore the possible influence of immunogenicity of SHR-1701 on efficacy, safety and PK;   **Phase 3 Study**  **Primary study objective**   - To evaluate the PFS (evaluated by IRC) achieved with SHR-1701 combined with BP102 and XELOX vs. placebo combined with BP102 and XELOX for the first-line treatment of patients with advanced colorectal cancer;   **Secondary study objectives**   - To evaluate the PFS (evaluated by the investigator), ORR, DoR, DCR and OS achieved with SHR-1701 combined with BP102 and XELOX vs. placebo combined with BP102 and XELOX for the first-line treatment of patients with advanced colorectal cancer; - To evaluate the safety of SHR-1701 combined with BP102 and XELOX vs. placebo combined with BP102 and XELOX for the first-line treatment of patients with advanced colorectal cancer;   **Exploratory objective:**   - To explore the possible influence of immunogenicity of SHR-1701 on efficacy, safety and PK; |
| **Endpoints** | **Phase 2 Study**  **Primary endpoints:**   - Safety: - ORR (RECIST 1.1 criteria, evaluated by the investigator);   **Secondary endpoints:**   - DCR (RECIST 1.1 criteria, evaluated by the investigator); - DOR (RECIST 1.1 criteria, evaluated by the investigator); - PFS (RECIST 1.1 criteria, evaluated by the investigator); - OS;   **Exploratory endpoints:**   - Immunogenicity indicators (ADA, NAb) of SHR-1701, and trough concentration of SHR-1701 (C_trough_);   **Phase 3 Study**  **Primary endpoint**   - PFS (RECIST 1.1 criteria, evaluated by IRC);   **Secondary endpoints:**   - PFS (RECIST 1.1 criteria, evaluated by the investigator); - ORR (RECIST 1.1 criteria); - DoR (RECIST 1.1 criteria); - DCR (RECIST 1.1 criteria); - OS; - Safety: AE, laboratory test measurements, etc.   **Exploratory endpoints:**   - Immunogenicity indicators (ADA, NAb) of SHR-1701, and trough concentration of SHR-1701 (C_trough_); |
| **Study population** | Patients with unresectable recurrent or metastatic advanced colorectal cancer who have not received any previous systematic anti-tumor therapy. |
| **Study design** | This study is designed to be a Phase 2/3 study, with Phase 2 being a single-arm, multicenter study, aimed to assess the safety and efficacy of SHR-1701 combined with BP102 and XELOX for the first-line treatment of patients with unresectable recurrent or distantly metastatic advanced colorectal cancer; and Phase 3 being a randomized, double-blind, placebo-controlled, multicenter study, aimed to assess the efficacy and safety of SHR-1701 combined with BP102 and XELOX vs. placebo combined with BP102 and XELOX for the first-line treatment of patients with unresectable recurrent or distantly metastatic colorectal cancer.  **Phase 2 Study**  In Phase 2, it is planned to enroll 61 subjects who will receive SHR-1701 combined with BP102 and XELOX, with the administration route and imaging assessment consistent with those in the Phase 3 test group, with safety and investigator-assessed ORR as the primary study endpoints. Whether to start Phase 3 will be determined according to the safety and efficacy results of Phase 2, and these results will be used as the reference for hypothesis of efficacy in Phase 3.  **Phase 3 Study**  Phase 3 is a randomized, double-blind, placebo-controlled, multicenter study, and it is planned to enroll 378 subjects. The eligible subjects will be randomized to receive SHR-1701 combined with BP102 and XELOX (test group) or placebo combined with BP102 and XELOX (control group) for treatment at a ratio of 1:1, with stratification factors as follows: PD-L1 expression (<1% or ≥1%), location of the primary tumor lesion (left or right) and RAS gene type (mutant type or wild type). PFS assessed by the independent review committee (IRC) based on RECIST v1.1 criteria will be used as the primary study endpoint.  It is planned to conduct an interim analysis when 70% PFS events are collected in Phase 3, and the purpose of this interim analysis is to demonstrate that PFS in the test group is superior to that in the control group.  Subjects will enter the screening period after comprehensive understanding of the study and signing the informed consent form. The screening period of the study is 28 days, and after completion of screening inspection and assessment, subjects who pass the screening will be randomized to the test group or the control group for corresponding treatment.  **Test group:** SHR-1701, 30 mg/kg, intravenous infusion, D1; BP102, 7.5 mg/kg, intravenous infusion, D1: oxaliplatin, 130 mg/m^2^, intravenous infusion, D1; capecitabine, 1000 mg/m^2^/time, oral administration, twice daily for two weeks in each cycle, followed by a week of rest.  **Control group:** Placebo, intravenous infusion, D1; BP102, 7.5 mg/kg, intravenous infusion, D1: oxaliplatin, 130 mg/m^2^, intravenous infusion, D1; capecitabine, 1000 mg/m^2^/time, oral administration, twice daily for two weeks in each period, followed by a period of rest.  For both the test group and the control group, one treatment cycle lasts 21 days, and subjects who have completed up to 8 cycles of combination with oxaliplatin and have no progressive disease or who can benefit from continuing the maintenance treatment in the opinion of the investigator can enter the maintenance treatment stage, and receive treatment with SHR-1701/ placebo combined with BP102 and capecitabine until progressive disease (PD), toxicity intolerance, start of new anti-tumor therapy, subjects’ voluntary withdrawal from the study, or the investigator judges that the subjects need to be withdrawn from the study. The route and dosage of administration in the maintenance phase are the same as before. The maximum medication duration of SHR-1701/ placebo /BP102/ capecitabine is 2 years.  The tumor imaging assessment begins at randomization (first administration in Phase 2), and the imaging examination will be performed every 6 weeks (±7 days) for the first 48 weeks and every 12 weeks (±7 days) thereafter to evaluate the efficacy. If clinically indicated, imaging examination and assessment may be conducted additionally at any time. All subjects will complete safety examination and imaging assessment at withdrawal visit. After that, they will enter the safety follow-up visit. The survival follow-up visit will be conducted once every two months starting from the last dose; For those subjects who discontinue the study not due to progressive disease confirmed by imaging assessment, they should continue to receive follow-up visits to monitor tumor progress, and continue to receive imaging assessment at the frequency specified in the protocol until progressive disease, start of new anti-tumor therapy, withdrawal of informed consent, loss to follow-up or death. |
| **Administration route** | **Phase 2 Study**  All subjects will receive treatment with SHR-1701 combined with BP102 and XELOX in the following sequence:  SHR-1701, 30 mg/kg, intravenous infusion (30-60 min, no more than 2 hours), D1, once every 21 days;  BP102, 7.5 mg/kg, intravenous infusion, D1, once every 21 days (above 90 min for the first intravenous infusion, and if the first intravenous infusion is well tolerated, the second infusion can be shortened to 60 min, and if the 60 min of infusion is also well tolerated, the subsequent infusions can be completed within 30 min);  Oxaliplatin, 130 mg/m^2^, intravenous infusion (at least 2 hours), D1, once every 21 days;  Capecitabine, 1000 mg/m^2^/time, oral administration, twice daily (within 30 min after meal) consecutively for two weeks in each cycle (21 days as one cycle), followed by a week of rest.  Subjects who have completed up to 8 cycles of combination with oxaliplatin and have no progressive disease or who can benefit from continuing the maintenance treatment in the opinion of the investigator can enter the maintenance treatment stage, and receive treatment with SHR-1701 combined with BP102 and capecitabine until progressive disease (PD), toxicity intolerance, start of new anti-tumor therapy, subjects’ voluntary withdrawal from the study, or the investigator judges that the subjects need to be withdrawn from the study. The route and dosage of administration in the maintenance phase are the same as before. The maximum medication duration of SHR-1701/BP102/ capecitabine is 2 years.  **Phase 3 Study**  **Test group (to be dosed in the following sequence):**  SHR-1701, 30 mg/kg, intravenous infusion (30-60 min, no more than 2 hours), D1, once every 21 days;  BP102, 7.5 mg/kg, intravenous infusion, D1, once every 21 days (above 90 min for the first intravenous infusion, and if the first intravenous infusion is well tolerated, the second infusion can be shortened to 60 min, and if the 60 min of infusion is also well tolerated, the subsequent infusions can be completed within 30 min);  Oxaliplatin, 130 mg/m^2^, intravenous infusion (at least 2 hours), D1, administered once every 21 days;  Capecitabine, 1000 mg/m^2^/time, oral administration, twice daily (within 30 min after meal) consecutively for two weeks in each cycle (21 days as one cycle), followed by a week of rest.  **Control group (to be dosed in the following sequence):**  Placebo, intravenous infusion (30-60 min, no more than 2 hours), D1, once every 21 days;  BP102, 7.5 mg/kg, intravenous infusion, D1, once every 21 days (above 90 min for the first intravenous infusion, and if the first intravenous infusion is well tolerated, the second infusion can be shortened to 60 min, and if the 60 min of infusion is also well tolerated, the subsequent infusions can be completed within 30 min);  Oxaliplatin, 130 mg/m^2^, intravenous infusion (at least 2 hours), D1, administered once every 21 days;  Capecitabine, 1000 mg/m^2^/time, oral administration, twice daily (within 30 min after meal) consecutively for two weeks in each cycle (21 days as one cycle), followed by a week of rest.  Subjects who have completed up to 8 cycles of combination with oxaliplatin and have no progressive disease or who can benefit from continuing the maintenance treatment in the opinion of the investigator can enter the maintenance treatment stage, and receive treatment with SHR-1701/placebo combined with BP102 and capecitabine until progressive disease (PD), toxicity intolerance, start of new anti-tumor therapy, subjects’ voluntary withdrawal from the study, or the investigator judges that the subjects need to be withdrawn from the study. The route and dosage of administration in the maintenance phase are the same as before. The maximum medication duration of SHR-1701/ placebo /BP102/ capecitabine is 2 years. |
| **Inclusion criteria** | **Patients must meet all of the following criteria to be enrolled in this study:**   1. Patients participate in this study voluntarily and sign the informed consent form; 2. Age ≥ 18 years and ≤75 years, both males and females; 3. Patients with unresectable recurrent or metastatic colorectal adenocarcinoma diagnosed by histology; 4. The subjects are required to provide tissue samples for biomarker detection (such as RAS gene type and PD-L1 expression), the newly acquired tissues are preferred, and patients who cannot provide newly acquired tissues can provide 10-15 archived paraffin sections with a thickness of ≥3μM; 5. Patients who have not received any previous systemic anti-tumor therapy (including but not limited to systemic chemotherapy, molecular targeted therapy, immunotherapy, biological therapy or other study drugs); for subjects who have received previous neo-adjuvant or adjuvant therapy, the first discovery of recurrence or metastasis must be ≥12 months after the last administration of the neo-adjuvant or adjuvant therapy; 6. There is at least 1 measurable lesion according to RECIST v1.1 criteria (lesions which have received previous local treatment such as radiotherapy cannot be regarded as measurable lesions); 7. ECOG score 0-1; 8. Expected survival ≥ 3 months; 9. The functions of important organs meet the following requirements (no blood components, cell growth factors or related drugs are allowed to be used for correction within 2 weeks before the start of study treatment): 10. Absolute neutrophil count (ANC) ≥ 1.5 × 10^9^/L 11. Platelets ≥100×10^9^/L; 12. Hemoglobin ≥9 g/dL; 13. Serum albumin ≥3.0g/dL; 14. Total bilirubin ≤1.5 × ULN, ALT, AST and/or AKP ≤ 2.5× ULN; in case of liver metastasis, ALT and/or AST ≤5 × ULN, and total bilirubin ≤ 3× ULN; in case of liver metastasis or bone metastasis, AKP ≤5 × ULN 15. Creatinine clearance rate ≥50mL/min (calculated according to Cockcroft-Gault equation) or serum creatinine ≤ 1.5× ULN; 16. International standardized ratio (INR) ≤ 1.5×ULN, prothrombin time (PT) and activated partial thromboplastin time (APTT) ≤ 1.5×ULN; 17. Urinary protein < 2+ (if urinary protein is ≥2+, 24-hour (h) urinary protein quantification can be performed, and subjects with 24 h urinary protein quantification < 2.0 g can be enrolled). 18. Women with childbearing potential must agree to abstain from sexual intercourse (avoid heterosexual intercourse) or use reliable and effective methods of contraception from the signing of informed consent form to at least 6 months after the last dose of the investigational drug. Furthermore, the serum HCG test must be negative within 3 days before the start of the study treatment, and the subjects must be in non-lactating period. A female patient is considered to have childbearing potential if she has menstruated, has not yet achieved the post-menopausal state (non-menstrual period ≥12 consecutive months, and no other causes have been found except menopause), and has not received sterilization surgery (such as hysterectomy, bilateral tubal ligation or bilateral oophorectomy); 19. Male patients whose partners are women with childbearing potential must agree to abstain from sexual intercourse or use reliable and effective methods of contraception from the signing of informed consent form to at least 6 months after the last dose of the investigational drug. Male patients must also agree not to donate sperm during the same period. Male subjects whose partners are pregnant must use condoms, and other contraceptive methods are unnecessary. |
| **Exclusion criteria** | **Patients meeting any of the following criteria cannot be enrolled in this study**   1. Patients with recurrent or metastatic lesions that can be treated by radical surgery; 2. Patients with central nervous system or meningeal metastasis; 3. The subjects’ tumor type is known to be mis-match repair deficiency/microsatellite instability-high (dMMR/MSI-H); 4. Patients with bleeding tendency, high bleeding risk, coagulation dysfunction or thrombosis tendency: 5. Patients with a history of thrombotic disease within 6 months and/or hemoptysis within 3 months (coughing up at least 1/2 teaspoon of blood (about 2.5 mL) at a time); 6. Patients who have received full-dose oral or injectable anticoagulants or thrombolytic drugs for treatment purposes within 7 days before the start of the study treatment, however, preventive anticoagulation treatment for open intravenous infusion system and preventive use of low molecular weight heparin (enoxaparin ≤40 mg/ day) are allowed; 7. Patients who have used aspirin (> 325 mg/ day), dipyridamole, ticlopidine, clopidogrel, cilostazol or other drugs that inhibit platelet function within 7 days before the start of study treatment; 8. Patients whose CR/MRI suggests that tumor surrounds or invades large blood vessels (such as pulmonary artery or superior vena cava); 9. Patients with moderate or severe ascites with clinical symptoms (that is, those who need therapeutic puncture and drainage within 2 weeks before starting the study treatment, while those who only show a small amount of ascites on imaging without clinical symptoms can be enrolled); uncontrolled or moderate or severer pleural effusion and pericardial effusion; 10. Subjects whose toxicity from previous anti-tumor therapies has not recovered to ≤ CTCAE grade 1 (except for alopecia, achieving the numerical requirements in the inclusion criteria, or other AEs which will not affect the treatment with investigational drug in the opinion of the investigator); 11. Subjects with poorly controlled hypertension (systolic blood pressure ≥140 mmHg and/or diastolic blood pressure ≥90 mmHg under regular antihypertensive treatment), and subjects with previous history of hypertensive crisis or hypertensive encephalopathy; 12. Patients with severe cardiovascular and cerebrovascular diseases, including cerebrovascular accident (CVA), transient ischemic attack (TIA), myocardial infarction and significant vascular diseases (including but not limited to aortic aneurysm requiring surgical repair or recent arterial thrombosis) within 6 months prior to enrollment; patients with poorly controlled clinical symptoms or diseases of the heart, such as unstable angina pectoris, heart failure of NYHA (New York Heart Association ) Grade II or above, left ventricular ejection fraction < 50% by color Doppler echocardiography, or serious arrhythmia that cannot be controlled by medication; 13. Patients who have received major surgery within 4 weeks before the start of the study treatment (major surgery is defined as surgery under general anesthesia that requires at least 3 weeks of recovery time before receiving the study drug treatment); patients with incurable wounds (severe, unhealed or open), active peptic ulcers or untreated fractures; patients with gastrointestinal bleeding (including melena, hematochezia, etc., if the bleeding is confirmed as hemorrhoid bleeding or only manifested as occult blood in stool, the patient can be enrolled), tracheoesophageal fistula, gastrointestinal perforation or gastrointestinal fistula, or abdominal abscess within 6 months before the start of the study treatment; patients with extragastrointestinal bleeding (such as abnormal vaginal bleeding, hematemesis) with CTCAE Grade 3 or above within 6 months or with CTCAE Grade 2 or above within 3 months before the start of the study treatment; 14. Patients with intestinal obstruction or symptoms and signs of intestinal obstruction within 6 months before the start of treatment, but subjects who have received surgical treatment and whose obstruction is completely relieved can be screened; patients who have received previous intestinal stent implantation and whose intestinal stent has not been removed until the screening period; 15. Patients who were found to have active tuberculosis infection by medical history or CT examination, or who have had a medical history of active tuberculosis infection within 1 year prior to enrollment, or who have had active tuberculosis infection before more than 1 year but have not received regular treatment; 16. Patients with active autoimmune disease or history of autoimmune disease (including but not limited to: autoimmune hepatitis, interstitial pneumonia, uveitis, enteritis, hypophysitis, vasculitis, nephritis, hyperthyroidism and hypothyroidism [subjects whose disease can be controlled just by thyroid hormone replacement therapy can be included]); subjects with skin diseases that do not need systematic treatment (such as vitiligo, psoriasis, alopecia), controlled type I diabetes treated with insulin, or asthma that has been completely relieved in childhood and does not need any intervention in adulthood, can be included (patients with asthma who need medical intervention with bronchodilators cannot be included); 17. Subjects with severe infection (CTCAE ≥Grade 3) within 4 weeks before the start of study and treatment, including but not limited to bacteremia, severe pneumonia or other serious infection complications requiring hospitalization; patients with active infection or unexplained fever > 38.5℃ within 2 weeks before the first dose (subjects with fever due to tumor (as judged by the investigator) can be included); patients who have received oral or intravenous administration of therapeutic antibiotics within 2 weeks before the start of the study (patients who use preventive antibiotics can participate in this study); 18. Subjects who are currently accompanied by interstitial pneumonia or interstitial lung disease, or have a history of previous interstitial pneumonia or interstitial lung disease requiring hormone therapy; or subjects with pulmonary fibrosis, organized pneumonia (for example, bronchiolitis obliterans), pneumoconiosis, drug-related pneumonia, or idiopathic pneumonia that may interfere with the judgment and treatment of immune related pulmonary toxicities; or subjects with evidence of active pneumonia or severe impairment of lung function according to the chest computed tomography (CT) images during the screening period; 19. Patients with congenital or acquired immunodeficiency (such as HIV infection); a history of organ transplantation or allogeneic bone marrow transplantation; active hepatitis B (HbcAb and/or HbsAg positive, and HBV-DNA ≥ 2500 copies /mL or 500 IU/ml) or hepatitis C (HCV antibody positive, and HCV-RNA higher than the limit of detection of the analysis method); complicated with hepatitis B and hepatitis C infection (HbsAg or HbcAb positive and HCV antibody positive); 20. Patients who have been diagnosed with other malignant tumors within 5 years before the first use of the study drug, however, patients with effectively treated skin basal cell carcinoma, skin squamous cell carcinoma and/or effectively resected in situ cervical cancer and/or breast cancer may be allowed to be included after assessment; 21. Patients with known allergic reaction, hypersensitivity or contraindication to SHR-1701/ placebo, BP102, oxaliplatin, capecitabine or any component used in these products; 22. Patients who have received treatment with immunosuppressants or corticosteroids (prednisone at a dose of > 10 mg/day or equivalent doses of other hormones) within 14 days before the start of the study treatment for the purpose of immunosuppression; in the absence of active autoimmune diseases, steroid hormones are allowed to be inhaled or used locally, and hormone replacement therapy with prednisone at a dose of ≤ 10mg/ day or equivalent doses of other hormones is allowed; 23. Patients who have participated in other clinical studies within 4 weeks prior to the use of investigational drug in this study (for the subjects who have entered the follow-up visit period, the duration is calculated based on the time of the last use of investigational drug or device) or are participating in other clinical studies. 24. Patients who have previously received treatment with targeted T cell costimulatory molecules or immune checkpoint inhibitors (including but not limited to PD-1/PD-L1 inhibitors, CTLA-4 inhibitors, etc.); patients who have previously received anti-epidermal growth factor receptor therapy (including but not limited to cetuximab, panizumab, etc.) or any anti-angiogenic drug therapy (including but not limited to bevacizumab or its biosimilars, regorafenib, fruquintinib, etc.); 25. Patients who have received live attenuated vaccine treatment within 28 days before the start of the study treatment, or who are expected to need such vaccine during the treatment period or within 60 days after the last dose; 26. Patients who have received radiotherapy for the primary lesions within 6 months before the start of the study treatment; palliative treatment for non-target lesions is allowed to control symptoms, such treatment must be completed 4 weeks before the start of study treatment; patients who have used traditional Chinese medicine or Chinese patent medicine with anti-tumor effect within 2 weeks before the start of the study treatment; 27. Pregnant or lactating women; 28. Other factors of the subjects that may affect the study results or lead to forced discontinuance of the study (as judged by the investigator), such as alcohol abuse, drug abuse, other serious diseases (including mental diseases) requiring combined treatment, seriously abnormal laboratory test values, family or social factors, and other conditions that may affect the safety of the subjects or the collection of study data. |
| **PK/immunogenicity evaluation** | All subjects should undergo PK and immunogenicity blood sample collection. The time points of blood collection are as follows: On C1D1, C2D1, C4D1, C7D1 and Day 1 of every 6 cycles thereafter, blood samples should be collected within 0.5 hour before the administration of SHR-1701/ placebo; in case of suspension of the administration of SHR-1701/ placebo, the scheduled pre-dose PK and immunogenic blood samples should still be collected as much as possible, and for subjects who cannot pay a return visit, the blood samples should be collected at the next visit. In case of permanent discontinuation of SHR-1701/ placebo (regardless of whether other drugs are discontinued) and it has been more than 28 days since the last dose, subjects should pay a return visit as soon as possible and have blood samples collected. Subjects may select to have blood samples collected either at the end-of-(SHR-1701/placebo) treatment visit or 30 days (±7 days) after the last dose of SHR-1701/placebo, and then have blood samples collected 60 days (±7 days, if applicable) and 90 days (±7 days, if applicable) after the last dose of SHR-1701/placebo, respectively. At each time point, about 6 mL of venous blood will be collected for PK and immunogenicity tests of SHR-1701.  PK and immunogenicity blood samples should be collected as scheduled, but for the purpose of PK and immunogenicity analysis, unscheduled blood samples may also be collected.  The concentration of SHR-1701 in human serum is detected by the validated enzyme-linked immunosorbent assay (ELISA), the anti-SHR-1701 antibody (ADA) is detected by the validated electrochemiluminescence method based on agarose affinity purification, and the anti-SHR-1701 neutralizing antibody (NAb) is detected by the validated electrochemiluminescence method based on competitive ligand binding. |
| **Safety evaluation** | The severity of adverse events will be judged based on CTCAE v5.0 criteria. During the study, the record form of adverse events should be filled in truthfully, including their onset time, severity, relation with the study treatment, duration, actions taken and outcome. |
| **Efficacy evaluation** | **Phase 2 Study**  The primary efficacy endpoint is ORR: the objective response against tumor is evaluated according to RECIST v1.1, and the imaging evaluation is conducted every 6 weeks (±7 days) in the first 48 weeks and every 12 weeks (±7 days) thereafter. The best overall response is calculated by dividing the number of subjects who have achieved CR or PR by the total number, and the CR and PR after any new anti-tumor therapy will not be included in the calculation of ORR.  **Phase 3 Study**  The primary efficacy endpoint is PFS: it refers to the duration from randomization to the first occurrence of imaging evidence of disease progression or death (whichever comes first). Evaluation of primary efficacy endpoint PFS and other secondary/exploratory efficacy endpoints (ORR, DCR, DoR) according to RECIST v1.1 criteria: imaging evaluation is conducted every 6 weeks (±7 days) in the first 48 weeks starting from randomization and every 12 weeks (±7 days) thereafter. Once suspicious clinical disease progression occurs, the subjects should receive physical examination and it is necessary to confirm the imaging immediately, instead of waiting for the next scheduled imaging. |
| **Determination of sample size** | **Phase 2 Study**  Phase 2 is a single-arm study, with Simon two-stage design,, in which Optimal method is used to calculate the sample size based on ORR (the primary endpoint) evaluated by the investigator. That is, in the case of ineffective treatment, the sample size should be minimized, so that as few subjects as possible are exposed to the ineffective treatment in Phase 2.  The α level is controlled at 0.025, the power is 80%. Assuming that the unacceptable response rate is ORR=40%, and the expected target response rate is ORR=60%, at least 16 subjects will be enrolled first. If ≥8 of the first 16 subjects who have obtained post-dose efficacy assessment have achieved response (CR/PR), enrollment will be further performed until 61 subjects, and if < 8 subjects have achieved response, the study will be terminated. After 61 subjects are enrolled, if ≥32 subjects achieve response (CR or PR), it is suggested that further development can be carried out; if < 32 subjects achieve response, the investigator and the sponsor will discuss whether to carry out follow-up research and development.  **Phase 3 Study**  Phase 3 is a randomized controlled study. Assuming that the median PFS of the control group is 10 months, the estimated hazard ratio (HR) of PFS (test group/control group) is 0.68, that is, the median PFS of the test group is 14.7 months. If the overall α level is controlled at one-sided 0.025, and the original hypothesis that the distribution of PFS is consistent between the two groups is tested by log-rank method, collecting 245 PFS events can provide a power of 85%. Assuming that the enrollment time is 12 months and the whole study duration is 26 months, and considering that 5% of the subjects drop every year, it is estimated that a total of 378 subjects are needed.  PFS data of Phase 2 subjects will be collected before the completion of phase 3 enrollment, and it is possible to update the efficacy hypothesis and increase the sample size or the number of expected events in Phase 3 based on the PFS results of Phase 2. Since the data results of Phase 3 are not used in adjustment of the sample size, α consumption is not involved.  It is planned to conduct an interim analysis when 70% PFS events (172 events) are collected in Phase 3, and the purpose of this interim analysis is to demonstrate that PFS in the test group is superior to that in the control group. |
| **Statistical analysis** | **Analysis set/population**  **Phase 2 Study**  Full analysis set (FAS-S1): subjects who receive the investigational drug at least once after enrollment in Phase 2;  Evaluable set (ES-S1): a subset of FAS-S1, including subjects who are enrolled and receive the investigational drug at least once, and receive at least tumor assessment after receiving treatment;  **Phase 3 Study**  Full analysis set (FAS-S2): subjects who receive the investigational drug at least once after randomization in Phase 3 according to the ITT principle;  Safety set (SS-S2): subjects who receive the investigational drug at least once in Phase 3 (regardless whether they are randomized);  **Statistical methods**  **Basic method**  Phase 2 of this study is designed to be a single-arm study, and Phase 3 is designed to be a parallel controlled study. Unless otherwise specified, all data will be analyzed by treatment stage and treatment group and using the corresponding statistics according to the data type: the measurement data are expressed as mean, standard deviation, median, minimum and maximum, the counting data are expressed as frequency and percentage, and for time-to-event data, the median survival time of each group will be estimated by Kaplan-Meier (KM) product limit method. The survival curve will be plotted and the 95% confidence interval of the median time will be estimated if necessary.  **Efficacy analysis**   1. **Primary efficacy endpoint analysis**   The efficacy analysis in Phase 2 will be conducted for the primary efficacy endpoint, the ORR evaluated by the investigator. The descriptive statistical analysis of ORR will be conducted based on the full analysis set (FAS-S1) of Phase 2. The descriptive statistical analysis of Phase 2 evaluable set (ES-S1) will be used as an auxiliary analysis.  The primary efficacy analysis in Phase 3 will be conducted for the primary efficacy endpoint, the PFS evaluated by IRC. Based on the Phase 3 full analysis set (FAS-S2), the end point PFS will be tested by stratified log-rank test based on stratification factors. The survival curve will be plotted by Kaplan-Meier method. The Greenwood's method will be used to calculate the standard error, and the Brookmeyer-Crowley method based on log-log transformation will be used to estimate the two-sided 95% confidence interval for median PFS.   1. **Supporting analysis**   The supporting analysis of the primary endpoint is only conducted in Phase 3. In Phase 3, the supporting analysis of PFS evaluated by IRC will be conducted based on PPS-S2 with the same method used for the primary efficacy analysis. In Phase 3, the stratified Cox proportional hazard model will be used to estimate the hazard ratio and 95% confidence interval of PFS evaluated by IRC in the test group in relation to the control group. In addition, in order to explore the influence of other factors on efficacy, in addition to the treatment group, other factors may also be added to the model as covariates to be involved in the model fitting.  **Secondary efficacy endpoint analysis**  The secondary efficacy analysis in Phase 2 of this study is based on DCR, PFS, DOR and OS evaluated by the investigator. The above-mentioned efficacy endpoints will be statistically analyzed based on the full analysis set (FAS-S1) in Phase 2.  The secondary efficacy analysis in Phase 3 will be based on the full analysis set (FAS-S2) in Phase 3, and will be based on ORR, DCR, PFS, DOR and OS evaluated by the investigator.  In Phase 3, for the endpoints OS and PFS evaluated by the investigator, the differences between groups will be compared by the stratified log-rank method and HR will be estimated based on Cox proportional hazard model, i.e., the same methods used for analyzing the primary endpoint, PFS evaluated by IRC, in Phase 3.  The analysis of DOR evaluated by the investigator in Phase 3 is limited to the subjects who achieve response, and the same analysis method is used as that for the primary endpoint in Phase 3, PFS evaluated by IRC.  For the ORR and DCR evaluated by the investigator, the test group and control group are summarized by descriptive statistical method, and the 95% confidence interval of a single treatment group are estimated by Wilson (Score) method. 95% CI of the rate difference between the two groups will be estimated by Newcombe method. The analysis of DOR will be limited to the subjects who have achieved response, and the corresponding descriptive analysis will be performed.  **Safety analysis**  Safety analysis will be based on Phase 2 FAS-S1 and Phase 3 safety set (SS-S2) (actual medication group). Safety analysis is limited to descriptive statistical summary, including but not limited to the following aspects:   - Summary of treatment-emergent adverse events (all-cause and treatment-related); - Incidence and severity of treatment-emergent adverse events (all-cause and treatment-related); - Summary of details of treatment-emergent serious adverse events; - Analysis of correlation of treatment-emergent adverse events; - Incidence of dose suspension, dose reduction and dose termination due to drug-related toxicity during the study; - Laboratory indicators, vital signs, ECG data and changes from baseline;   Number and ratio of cases of “normal to abnormal” or “worsening abnormality” in laboratory indicators, vital signs, ECG data after treatment. |
| **Interim analysis** | In the Phase 3 randomized controlled study, an interim analysis is planned for the primary efficacy endpoint, the progression free survival (PFS) evaluated by IRC, and it is planned to conduct this analysis when about 172 events are collected. The objectives of the interim analysis include:   1. Terminate the study early because of superior effect; 2. Continue the study as scheduled;   Judgment rule for study termination at interim analysis due to superior effect: determine the statistically significant analysis boundary according to Lan-DeMets α consumption function and O'Brien-Fleming boundary.  If the interim analysis does not terminate the study due to superior effect, the final analysis will be performed when about 245 PFS events are collected (estimated to be 26 months after enrollment of the first patient). According to Lan-DeMets α consumption function, when the single-sided *p* value is less than 0.023, it suggests that the efficacy in the test group is superior to that in the control group. |

Study schedule

| **Item** | **Screening period ^[2]^** | | **Treatment period ^[3]^ (including the maintenance treatment period) ^[29]^** | | | **End of treatment/withdrawal from the study ^[34]^**  **(±3 days)** | | **Safety follow-up ^[35]^** | | **Survival follow-up ^[36]^ (±7 days)** |
| --- | --- | --- | --- | --- | --- | --- | --- | --- | --- | --- |
|  |  |  | **Cycle 1** | **Cycle 2** | **Cycle 3** |  |  | **First** | **Second/third** |  |
|  | **D28-D1** | **D7-D1** | **C1D1** | **C2D1 (±3 days)** | **C3D1 (±3 days)** |  |  |  |  |  |
| Signing of informed consent form **^[1]^** | √ |  |  |  |  |  | |  |  |  |
| Verification of inclusion and exclusion criteria | √ | √ |  |  |  |  | |  |  |  |
| Demographics | √ |  |  |  |  |  | |  |  |  |
| Tumor medical history ^[4]^ | √ |  |  |  |  |  | |  |  |  |
| Other past medical history^[5]^ | √ |  |  |  |  |  | |  |  |  |
| ECOG-PS^[6]^ |  | √ |  | √ | √ | √ | |  |  |  |
| Physical examination ^[7]^ |  | √ |  | √ | √ | √ | |  |  |  |
| Vital signs ^[8]^ |  | √ |  | √ | √ | √ | |  |  |  |
| Hematology ^[9]^ |  | √ |  | √ | √ | √ | | √ |  |  |
| Urinalysis ^[10]^ |  | √ |  | √ | √ | √ | |  |  |  |
| Fecal occult blood ^[11]^ |  | √ |  | √ | √ | √ | |  |  |  |
| Blood biochemistry ^[12]^ |  | √ |  | √ | √ | √ | | √ |  |  |
| Coagulation function ^[13]^ |  | √ |  |  |  | √ | |  |  |  |
| CEA^[14]^ |  | √ |  |  | √ | √ | |  |  |  |
| Thyroid function ^[15]^ |  | √ |  | √ | √ | √ | | √ |  |  |
| 12-lead ECG ^[16]^ |  | √ |  | √ | √ | √ | |  |  |  |
| Echocardiography ^[17]^ |  | √ |  |  |  | √ | |  |  |  |
| HCG test ^[18]^ |  | √ |  |  |  | √ | |  |  |  |
| Virological test ^[19]^ | √ |  |  |  |  |  | |  |  |  |
| Tumor imaging examination ^[20]^ | √ |  | √ | | | √ | |  |  |  |
| Tumor tissue samples ^[21]^ | √ |  |  |  |  |  | |  |  |  |
| Biomarker test ^[22]^ | √ |  |  |  |  |  | |  |  |  |
| Randomization ^[23]^ |  | √ |  |  |  |  | |  |  |  |
| PK/immunogenicity blood collection ^[24]^ |  |  | √ | √ |  | √ | | √ | √ |  |
| SHR-1701/placebo administration ^[25]^ |  |  | √ | √ | √ |  | |  |  |  |
| BP102 administration ^[26]^ |  |  | √ | √ | √ |  | |  |  |  |
| Oxaliplatin administration ^[27]^ |  |  | √ | √ | √ |  | |  |  |  |
| Capecitabine administration^[28]^ |  |  | √ | √ | √ |  | |  |  |  |
| Drug recovery ^[30]^ |  |  |  | √ | √ | √ | |  |  |  |
| Prior and concomitant medication/treatment ^[31]^ | √ | √ | √ | √ | √ | √ | | √ | √ | √ |
| Adverse events ^[32]^ | √ | √ | √ | √ | √ | √ | | √ | √ | √ |
| Recording of administered doses ^[33]^ |  |  | Continuously record, until the study treatment of the subjects terminates | | | | |  |  |  |
| Distribution, verification and recovery of subject diary ^[37]^ |  |  | √ | √ | √ | | √ |  |  |  |
| Survival information |  |  |  |  |  | |  |  | √ | √ |
| Subsequent anti-tumor therapy information |  |  |  |  |  | |  |  | √ | √ |

ECOG-PS: Eastern Cooperative Oncology Group - Performance Status; CEA: Carcinoembryonic antigen; HCG: Human chorionic gonadotropin; PK: Pharmacokinetics;

Note: In addition to the test items and time points listed in the schedule above, the investigator may add items that need to be tested at any time, and the test results should be filled in "unscheduled test" in CRF;

1. Before screening, the written informed consent signed by the subject or his/her guardian/notary witness must be obtained first;
2. Screening period: after the informed consent form is signed, the baseline information of the subjects will be collected, which should be conducted no more than 28 days before the start of study medication; if conventional tumor imaging evaluation has been performed before signing the informed consent, and such enhanced CT or MRI were completed within 28 days before the start of the study medication (for bone scanning, results within 42 days before the start of the study medication are acceptable), the tumor imaging does not need to be repeated during the screening period; among all the baseline assessment values, those obtained closest to the time of study medication should be selected; subjects who fail the screening may be re-screened, and informed consent must be obtained again and a new subject number must be assigned at re-screening, and subjects are allowed to be re-screened for only once.
3. Treatment period: the subjects' ECOG score, vital signs, physical examination, hematology, urinalysis, fecal occult blood test, blood chemistry, thyroid function and ECG will be assessed within 3 days before the scheduled treatment visit (during the screening period, these tests will be completed within 7 days before the first dose); if there is a delay in drug administration, the corresponding test items of scheduled visits should be consistent with the actual drug administration time; if there is a long delay in drug administration, the test interval should not exceed 6 weeks (except PK and immunogenicity testing);
4. Tumor medical history: including pathological diagnosis, location of primary tumor lesion ("Left" is defined as from splenic flexure to rectum, "Right" is defined as from ileocecum to splenic flexure), test results of gene mutation, and treatment process (such as surgery, interventional therapy, radiotherapy, local ablation, chemotherapy, etc.);
5. Other past medical history: including history of drug allergy, history of diagnosis and treatment of other concomitant diseases, and history of tumors other than colorectal cancer;
6. ECOG score: within 7 days before the first dose, before dosing on Day 1 of each cycle from Cycle 2, at the end of treatment/withdrawal from the study;
7. Physical examination: A comprehensive physical examination will be conducted within 7 days before the first dose and at the withdrawal from the study, including height (collected only before the first dose), weight, head and face, skin system, lymph nodes, eyes, ears, nose and throat, mouth, respiratory system, cardiovascular system, abdomen, reproductive and urinary system, musculoskeletal system, nervous system, mental status and others. During the study, only the physical examinations concerning body weight and important or abnormal parts will be performed. Note: Except for the physical examinations during the screening period and at the end-of-treatment visit, it is not necessary to record the complete physical examination results in eCRF during the study period, but only the body weight and abnormal conditions;
8. Vital signs: blood pressure, pulse, body temperature, respiratory rate; within 7 days before the first dose, before dosing on Day 1 of each cycle from Cycle 2, and at the end of treatment/withdrawal from the study; it is suggested that the subjects monitor their blood pressure daily, and if they feel unwell or have high blood pressure, they should seek medical advice or contact the study physician in a timely manner.
9. Hematology test: including red blood cell count, hemoglobin, platelet count, white blood cell count, neutrophil count and lymphocyte count; within 7 days before the first dose, before dosing on Day 1 of each cycle from Cycle 2, at the end of treatment/withdrawal from the study, and 30 days after the last treatment;
10. Urinalysis: white blood cell, red blood cell and urinary protein: within 7 days before the first dose, before dosing on Day 1 of each cycle from Cycle 2, at the end of treatment/withdrawal from the study. If the urinary protein is ≥ 2+, it is necessary to conduct the 24 h quantitative determination of urinary protein. During the study, unscheduled examinations can be added as clinically indicated or at the discretion of the investigator;
11. Fecal occult blood: the FIT (fecal immunochemical test) method is preferred; within 7 days before the first dose, before dosing on Day 1 of each cycle from Cycle 2, and at the end of treatment/withdrawal from the study; if the subjects show changes in stool characteristics (melena, hematochezia, etc.) or testing is required in the opinion of the investigator, an unscheduled test will be added;
12. Blood chemistry: ALT, AST, GGT, total bilirubin, direct bilirubin, AKP, blood urea nitrogen or urea (preferably blood urea nitrogen), total protein, albumin, creatinine, blood sugar, lactate dehydrogenase, K+, Na+, Ca2+, Mg2+, Cl-; within 7 days before the first dose, before dosing on Day 1 of each cycle from Cycle 2, at the end of treatment/withdrawal from the study and 30 days after the last treatment;
13. Coagulation function: APTT, PT, FIB and INR. Within 7 days before the first dose, at the end of treatment/withdrawal from the study; it will be tested as clinically indicated or at the discretion of the investigator during study;
14. CEA: within 7 days before the first dose, before dosing on Day 1 of Cycle 2, at the end of treatment/withdrawal from the study;
15. Thyroid function test: TSH, FT3, FT4; within 7 days before the first dose, before dosing on Day 1 of each cycle from Cycle 2, at the end of treatment/withdrawal from the study, and 30 days after the last treatment;
16. 12-lead ECG: heart rate, PR interval, QT interval, QTcF. Within 7 days before the first dose, before dosing on Day 1 of each cycle from Cycle 2, at the end of treatment/withdrawal from the study;
17. Echocardiography: it will be performed within 7 days before the first dose, at the end of treatment/withdrawal from the study, and during the study, unscheduled echocardiography may be added at the discretion of the investigator;
18. Blood HCG test: only for women of childbearing age, serum pregnancy test will be performed; within 3 days before the first dose, at the end of treatment/withdrawal from the study, and at the discretion of the investigator in other necessary situations;
19. Virological test: quantitative determination of HbsAg, HBsAb, HBeAg, HBeAb, HBcAb and HBV DNA (if the results of "five hepatitis B test items” show that there is hepatitis B virus infection [HbcAb and/or HBsAg positive] or there is a past history of hepatitis B virus infection, HBV DNA quantitative determination is required; if both HBsAb and HBcAb are positive, HBV DNA quantitative determination may not be performed); HCV-Ab (if HCV-Ab is positive, HCV-RNA quantitative test is required); and HIV-Ab; within 14 days before the first dose;
20. Tumor imaging examination: CT or MRI of chest and abdomen (including pelvic cavity) (enhanced CT or MRI, except for the situations where contract agent is contradicted and plain scanning can be used instead), MRI of brain is required in case of suspected or diagnosed brain metastasis (CT can be used instead where MRI is contradicted (enhanced CT or MRI, except for the situations where contract agent is contradicted and plain scanning can be used instead)), and bone scanning is only performed when clinically indicated.

- During the screening period, the imaging examination results obtained before signing the informed consent can be used for baseline tumor assessment as long as they meet the requirements of RECIST1.1, and the baseline imaging examination can be extended to 4 weeks before the first dose. Bone scanning is needed in case of suspected or confirmed bone metastasis, and the test can be extended to 42 days before the first dose;
- During the treatment period, the imaging examination will be performed every 6 weeks (±7 days) in the first 48 weeks and every 12 weeks (±7 days) thereafter. In case of suspicion of progressive disease, an unscheduled imaging examination may be performed. A subject who withdraws from the study for any reason should receive imaging examination in a timely manner (if the previous examination is less than 4 weeks from the withdrawal visit, re-examination is not required). The imaging examination conditions should be consistent with those at the baseline (including scanning thickness, contract agent, etc.);
- If a subject receives an unscheduled imaging examination, and it is less than 4 weeks from the next scheduled examination, it is unnecessary to perform the next scheduled imaging examination, and it can be resumed just at the scheduled time point thereafter;
- The allowable window period of imaging examination is ±7 days. In addition to the progressive disease confirmed by imaging, the subjects who terminate the study treatment for other reasons should also receive imaging examination at the scheduled imaging visit until progressive disease is observed, new anti-tumor therapy is started, the subjects withdraw their informed consent form, loss to follow-up or death.

1. Tumor tissue samples: the newly acquired tissues are preferred, or the archived tumor tissues will be collected;
2. Test of biomarkers: The biomarkers (RAS gene type, PD-L1 expression, etc.) in Phase 2 will be tested by the study site or using the previous test reports of subjects; RAS gene type and PD-L1 expression should be tested in the central laboratory before Phase 3 randomization.
3. Randomization: The subjects will be assigned to the test group or the control group by randomization system after being confirmed to meet the inclusion and exclusion criteria, and the first dose should be given within 2 days after randomization.
4. PK/immunogenicity blood samples collected: All subjects should undergo PK and immunogenicity blood sample collection at the following time points: At C1D1, C2D1, C4D1, C7D1 and the Day 1 of every 6 cycles thereafter, blood samples should be collected within 0.5 hour before the administration of SHR-1701; in case of suspension of the administration of SHR-1701, the scheduled pre-dose PK and immunogenic blood samples should still be collected as much as possible, and for subjects who cannot pay a return visit, the blood samples should be collected at the next visit. In case of permanent discontinuation of SHR-1701/ placebo (regardless of whether other drugs have been discontinued) and it has been more than 28 days since the last dose, subjects should pay a return visit as soon as possible and have blood samples collected. Subjects may select to have blood samples collected either at the end-of-(SHR-1701/placebo) treatment visit or 30 days (±7 days) after the last dose of SHR-1701/placebo, and then have blood samples collected 60 days (±7 days, if applicable) and 90 days (±7 days, if applicable) after the last dose of SHR-1701/placebo, respectively; at each time point, about 6 mL of venous blood will be collected in a serum separation tube for PK and immunogenicity analysis of SHR-1701/placebo; the PK and immunogenicity blood samples will be collected according to the study schedule, but for the purpose of PK and immunogenicity analysis, unscheduled blood samples may also be collected;
5. Administration of SHR-1701/placebo: Day 1 of every cycle, with 3 weeks as a treatment cycle;
6. Administration of BP102: Day 1 of every cycle, with 3 weeks as a treatment cycle;
7. Administration of oxaliplatin: Day 1 of every cycle, with 3 weeks as a treatment cycle;
8. Administration of capecitabine: capecitabine will be dispensed on Day 1 of every cycle, administered for 2 consecutive weeks, followed by 1 week of rest, with every 3 weeks as a treatment cycle.
9. Maintenance therapy: The maintenance therapy consists of SHR-1701/placebo combined with BP102 and capecitabine;
10. Drug recovery: For capecitabine, on Day 1 of each cycle from Cycle 2, the remaining drug from the previous cycle will be recovered and the drug for the next cycle will be dispensed;
11. Prior and concomitant medication/treatment: All concomitant medications from 30 days before the first dose until the end of the safety follow-up period or until the start of new anti-tumor therapy (whichever comes first) will be recorded, and afterwards, only the concomitant medications for the treatment of adverse events related to the study drug will be recorded, and the vehicle and sealing related drugs are not required to be recorded in CRF;
12. Adverse events: The collection period starts from the signing of the informed consent form until 90 days after the last dose of SHR-1701/ placebo or 30 days after the last treatment of chemotherapy/targeted therapy (whichever is longer); if a patient starts a new anti-tumor therapy during the AE collection period, only the AEs related to the investigational drug will be collected after the new anti-tumor therapy;
13. Recording of administered doses: including the actual dosage, quantity, dose suspension and dosage adjustment of the subjects in each cycle;
14. End of treatment/withdrawal from the study: The end-of-treatment visit will not be conducted until all treatment drugs are discontinued permanently, and if the corresponding tests are completed 7 days prior to withdrawal from the treatment, these tests are not required during the visit;
15. Safety follow-up visit: After the last dose (permanent discontinuation of all drug treatments), subjects (including the subjects who have completed the end-of-treatment visit) should pay a safety follow-up visit; the safety follow-up period lasts until 90 days after the last dose of SHR-1701/ placebo or 30 days after the last treatment of chemotherapy/BP102, whichever is longer; if a patient starts a new anti-tumor therapy during the safety follow-up period, only AEs related to the investigational drug will be collected after the new anti-tumor therapy; the first safety follow-up visit is required 30 days (±7 days) after the last study dose, regardless of whether a new anti-tumor therapy has been started, and this visit must be conducted at the study site; 60 days (±7 days) and 90 days (±7 days) after the last study dose, the patient should pay a safety follow-up visit to the study site as far as possible, and have PK/ immunogenicity blood samples collected (if applicable); patients who indeed cannot go to the study site should receive a telephone visit to collect information on survival, subsequent anti-tumor therapy, concomitant medication/treatment, and AE/SAE; if it is less than 45 days from the completion of the first safety visit to the end of the safety follow-up period, the subsequent safety visit can be conducted only once on the last day (±7 days) of the safety follow-up period.
16. Survival follow-up: The survival follow-up period will start after completion of the safety follow-up period. After that, it will be conducted every 2 months (±7 days); survival follow-up visit will be completed by telephone to collect the subjects’ survival information and subsequent anti-tumor therapy information.
17. Distribution, verification and recovery of subjects’ diaries: Diaries will be distributed, verified and recovered on Day 1 of every cycle (on C1D1, diaries are only distributed), and at the end-of-treatment visit, the diaries are not distributed, but only verified and recovered.

Contents

[Synopsis 1](#_Toc144373313)

[Study schedule 12](#_Toc144373314)

[Contents 18](#_Toc144373315)

[List of Tables 22](#_Toc144373316)

[List of Figures 22](#_Toc144373317)

[Abbreviations 23](#_Toc144373318)

[1. Introduction 26](#_Toc144373319)

[1.1. Study background 26](#_Toc144373320)

[1.1.1. Epidemiology and treatment status of colorectal cancer 26](#_Toc144373321)

[1.1.2. Study progress of immunotherapy in the first-line treatment of colorectal cancer 26](#_Toc144373322)

[1.1.3. Anti-PD-L1/TGF-β bifunctional immunotherapy 27](#_Toc144373323)

[1.2. Scientific basis 28](#_Toc144373324)

[1.2.1. Study design basis 28](#_Toc144373325)

[1.2.2. Drug development rationale 31](#_Toc144373326)

[1.2.3. Rationales for regimen design 38](#_Toc144373327)

[1.2.4. Rationale for dose selection 39](#_Toc144373328)

[1.3. Potential Risks and benefits to study population 39](#_Toc144373329)

[1.3.1. Known potential risks 39](#_Toc144373330)

[1.3.2. Known possible benefits 40](#_Toc144373331)

[1.3.3. Potential benefit-risk assessments 41](#_Toc144373332)

[2. Study objectives and endpoints 41](#_Toc144373333)

[2.1. Objectives 41](#_Toc144373334)

[2.1.1. Phase 2 Study 41](#_Toc144373335)

[2.1.2. Phase 3 Study 41](#_Toc144373336)

[2.2. Endpoints 42](#_Toc144373337)

[2.2.1. Phase 2 Study 42](#_Toc144373338)

[2.2.2. Phase 3 Study 42](#_Toc144373339)

[3. Study design 43](#_Toc144373340)

[4. Study population 44](#_Toc144373341)

[4.1. Inclusion criteria 45](#_Toc144373342)

[4.2. Exclusion criteria 46](#_Toc144373343)

[4.3. Randomization criteria 49](#_Toc144373344)

[4.4. Requirements on life style 49](#_Toc144373345)

[4.4.1. Contraception 49](#_Toc144373346)

[4.5. Screening failure 50](#_Toc144373347)

[5. Study intervention 50](#_Toc144373348)

[5.1. Drug Assignment 50](#_Toc144373349)

[5.2. Supply of investigational drug 51](#_Toc144373350)

[5.2.1 Dosage form and packaging 51](#_Toc144373351)

[5.2.2. Preparation and dispensing 52](#_Toc144373352)

[5.3. Dose and administration 52](#_Toc144373353)

[5.3.1. Administration 52](#_Toc144373354)

[5.3.2. Dose interruption 56](#_Toc144373355)

[5.3.3. Treatment time 62](#_Toc144373356)

[5.3.4. Subject compliance 63](#_Toc144373357)

[5.4. Storage of investigational drug 63](#_Toc144373358)

[5.5. Record of investigational drugs 64](#_Toc144373359)

[5.5.1. Destruction of investigational drug 64](#_Toc144373360)

[5.6. Prior and concomitant medication 64](#_Toc144373361)

[5.6.1. Prior medication 64](#_Toc144373362)

[5.6.2. Concomitant medications or concomitant therapies 64](#_Toc144373363)

[5.6.3. Supportive care 66](#_Toc144373364)

[5.6.4. Rescue treatment 70](#_Toc144373365)

[5.7. Method to minimize bias 70](#_Toc144373366)

[5.7.1. Procedures of enrollment/randomization/blinding 70](#_Toc144373367)

[5.7.2. Evaluation of blindness 70](#_Toc144373368)

[5.7.3. Unblinding 71](#_Toc144373369)

[6. Study procedures 71](#_Toc144373370)

[6.1. Screening 72](#_Toc144373371)

[6.2. Treatment period 73](#_Toc144373372)

[6.3. End-of-treatment/study treatment withdrawal visit 75](#_Toc144373373)

[6.4. Follow-up period 76](#_Toc144373374)

[6.4.1. Safety follow-up visit 76](#_Toc144373375)

[6.4.2. Survival follow-up 76](#_Toc144373376)

[6.5. Unscheduled visits 77](#_Toc144373377)

[6.6. Subject's withdrawal from the study or permanent discontinuation of study treatment 77](#_Toc144373378)

[6.6.1. Withdraw from the study 77](#_Toc144373379)

[6.6.2. Permanent discontinuation of study treatment 77](#_Toc144373380)

[6.6.3. Loss to follow-up 78](#_Toc144373381)

[6.7. Premature termination or suspension of a study 78](#_Toc144373382)

[6.8. Definition of End of Study 79](#_Toc144373383)

[6.9. Further medication at the end of study 79](#_Toc144373384)

[7. Evaluation 79](#_Toc144373385)

[7.1. Efficacy evaluation 79](#_Toc144373386)

[7.1.1. Blind independent review committee (BIRC) 80](#_Toc144373387)

[7.2. Safety evaluation 80](#_Toc144373388)

[7.2.1. Pregnancy test 80](#_Toc144373389)

[7.2.2. Adverse event 80](#_Toc144373390)

[7.2.3. Laboratory safety evaluation 80](#_Toc144373391)

[7.2.4. Vital signs and physical examination 80](#_Toc144373392)

[7.2.5. 12 lead ECG 80](#_Toc144373393)

[7.3. Pharmacokinetic evaluation 80](#_Toc144373394)

[7.3.1. Blood sample collection for SHR-1701 PK analysis 80](#_Toc144373395)

[7.4. Immunogenicity 81](#_Toc144373396)

[8. Adverse event reporting 81](#_Toc144373397)

[8.1. Adverse event (AE) 81](#_Toc144373398)

[8.1.1. Definition of AE 81](#_Toc144373399)

[8.1.2. Criteria for judging the severity of AEs 82](#_Toc144373400)

[8.1.3. Judgment of relationship between AE and investigational product 82](#_Toc144373401)

[8.2. SAEs 82](#_Toc144373402)

[8.2.1. Definition of SAE 82](#_Toc144373403)

[8.2.2. Hospitalization 83](#_Toc144373404)

[8.2.3. Disease progression and death 83](#_Toc144373405)

[8.2.4. Other anti-tumor therapies 84](#_Toc144373406)

[8.2.5. Reporting system for SAEs 84](#_Toc144373407)

[8.3. AE of special interest (SIE) 84](#_Toc144373408)

[8.3.1. Abnormal liver test 85](#_Toc144373409)

[8.4. Pregnancy 85](#_Toc144373410)

[8.5. Follow-up visit of AEs/SAEs 86](#_Toc144373411)

[9. Clinical monitoring 86](#_Toc144373412)

[10. Statistical analysis 87](#_Toc144373413)

[10.1. Sample size 87](#_Toc144373414)

[10.2. Statistical analysis plan 88](#_Toc144373415)

[10.3. Statistical hypothesis 88](#_Toc144373416)

[10.4. Analysis population 89](#_Toc144373417)

[10.5. Statistical methods 89](#_Toc144373418)

[10.5.1. Basic method 89](#_Toc144373419)

[10.5.2. Primary efficacy endpoint analysis 89](#_Toc144373420)

[10.5.3. Secondary efficacy endpoint analysis 90](#_Toc144373421)

[10.5.4. Safety analysis 91](#_Toc144373422)

[10.5.5. Interim analysis 91](#_Toc144373423)

[10.5.6. Subgroup analysis 91](#_Toc144373424)

[10.5.7. Multiple comparisons/multiplicity 92](#_Toc144373425)

[10.5.8. Exploratory analysis 92](#_Toc144373426)

[11. Data monitoring committee 92](#_Toc144373427)

[11.1. Data monitoring committee (DMC) 92](#_Toc144373428)

[12. Data management method 92](#_Toc144373429)

[12.1. Data collection 92](#_Toc144373430)

[12.1.1. Completion of Electronic Case Report Form (eCRF) 92](#_Toc144373431)

[12.1.2. Use of Electronic Data Capture (EDC) system 92](#_Toc144373432)

[12.2. Data Management 93](#_Toc144373433)

[12.2.1. ECRF data review 93](#_Toc144373434)

[12.2.2. Data review meeting and database lock 93](#_Toc144373435)

[12.2.3. Data archiving 93](#_Toc144373436)

[13. Original Data and Original documents 93](#_Toc144373437)

[14. Quality Assurance and Quality Control 93](#_Toc144373438)

[15. Ethics 94](#_Toc144373439)

[15.1. Ethical Norms 94](#_Toc144373440)

[15.2. Independent ethics committee 94](#_Toc144373441)

[15.3. Informed Consent 95](#_Toc144373442)

[15.3.1. Informed consent form and other written information required by subjects 95](#_Toc144373443)

[15.3.2. Informed consent process and records 95](#_Toc144373444)

[15.4. Confidentiality of Subject Information 95](#_Toc144373445)

[15.5. Future Use of Preserved Specimens 96](#_Toc144373446)

[16. Publication of Study Results 96](#_Toc144373447)

[17. Finance and Insurance 96](#_Toc144373448)

[18. Reference 96](#_Toc144373449)

[Attachment 1 Response Evaluation Criteria in Solid Tumors version 1.1 (RECIST v1.1) 97](#_Toc144373450)

[Attachment 2 Performance Status Scoring Criteria (ECOG) 105](#_Toc144373451)

[Attachment 3 Percentage of Human Bone Marrow Content 106](#_Toc144373452)

[Attachment 4 Prohibited Traditional Chinese Medicine During Study 107](#_Toc144373453)

[Attachment 5 TNM Staging of Colorectal Cancer (Version 8) 108](#_Toc144373454)

List of Tables

[Table 1 Randomized controlled studies on first-line treatment of advanced colorectal cancer 30](#_Toc144372604)

[Table 2 administration route of investigational drug for injection 53](#_Toc144372605)

[Table 3 Criteria for confirmed PD 55](#_Toc144372606)

[Table 4 Dose adjustment for non-hematological toxicities of oxaliplatin 60](#_Toc144372607)

[Table 5 Dose adjustment for neurological toxicities of oxaliplatin 60](#_Toc144372608)

[Table 6 Dose adjustments for nonhematologic toxicities of capecitabine 61](#_Toc144372609)

[Table 7 Dose adjustments for hematologic toxicities of XELOX - neutropenia (ANC) 61](#_Toc144372610)

[Table 8 Dose adjustments for hematologic toxicities of XELOX - neutropenic fever (ANC) 61](#_Toc144372611)

[Table 9 Dose adjustments for hematologic toxicities of XELOX -thrombocytopenia 62](#_Toc144372612)

[Table 10 Criteria for administration of treatment/re-treatment with XELOX 62](#_Toc144372613)

[Table 11 Adverse reactions caused by glucocorticoids and suggestions for their prevention and treatment 69](#_Toc144372614)

[Table 12 Criteria for judging the severity of adverse events 82](#_Toc144372615)

[Table 13 Judgment criteria for abnormal liver function tests 85](#_Toc144372616)

[Table 14 Collection of AEs/SAEs/SIEs/pregnancy events 86](#_Toc144372617)

List of Figures

[Figure 1 Assessment of efficacy of M7824 in Phase 1 clinical study 36](#_Toc144372618)

[Figure 2 Study design diagram of Phase 2 44](#_Toc144372619)

[Figure 3 Study design diagram of Phase 3 44](#_Toc144372620)

[Figure 4 Schematic diagram of administration of capecitabine 54](#_Toc144372621)

Abbreviations

| **Abbreviations** |  | **Chinese interpretation** |
| --- | --- | --- |
| 12-Lead ECG | 12-Lead Electrocardiogram | 12-导联心电图 |
| ADA | Anti-Drug Antibody | 抗药性抗体 |
| AE | Adverse Event | 不良事件 |
| AKP | Alkaline Phosphatase | 碱性磷酸酶 |
| ALT | Alanine Aminotransferase | 谷氨酸丙氨酸氨基转移酶 |
| ANC | Absolute Neutrophil Count | 中性粒细胞计数 |
| ANOVA | Analysis Of Variance | 方差分析 |
| APC | Antigen Presentation Cell | 抗原递呈细胞 |
| AST | Aspartate Aminotransferase | 谷氨酸天门冬氨酸氨基转移酶 |
| AUC | Area Under The Curve | 药时曲线下面积 |
| BMI | Body Mass Index | 身体质量指数 |
| BUN | Blood Urea Nitrogen | 尿素氮 |
| CFDA | China Food And Drug Administration | 国家食品药品监督管理总局 |
| CK | Creatine Kinase | 肌酸激酶 |
| CK-MB | Creatine Kinase-MB | 肌酸激酶 MB 同工酶 |
| Cl^-^ | Blood Chlorine | 血氯 |
| Cr | Creatinine | 肌酐 |
| CR | Complete Remission | 完全缓解 |
| CRC | Colorectal Cancer | 结直肠癌 |
| CRF | Case Report Form | 病例报告表 |
| CT | Computer Tomography | 电子计算机断层扫描 |
| CTLA-4 | Cytotoxic T Lymphocyte Associated Antigen -4 | 细胞毒T淋巴细胞相关抗原-4 |
| D | Day | 天 |
| DCR | Disease Control Rate | 疾病控制率 |
| DLT | Dose-Limiting Toxicity | 剂量限制毒性 |
| dMMR | Mismatch Repair-Deficient | 错配修复基因缺失 |
| DMC | Data Monitoring Committee | 数据监查委员会 |
| DoR | Duration Of Response | 缓解持续时间 |
| EC | Ethics Committee | 伦理委员会 |
| ECOG | Eastern Cooperative Oncology Group | 东部肿瘤协作组体力状况评分标准 |
| EDC | Electronic Data Capture | 电子数据采集系统 |
| EGFR-TKI | Epidemal Growth Factor Receptor-Tyrosine Kinase Inhibitors | 表皮生长因子酪氨酸激酶抑制剂 |
| FT3 | Free Triiodothyromine | 游离三碘甲状腺原氨酸 |
| FT4 | Free Thyroxine | 游离甲状腺素 |
| GCP | Good Clinical Practice | 药物临床试验质量管理规范 |
| GGT | Gramma Glutamyl Transpeptdase | 谷氨酰转肽酶 |
| GLU | Blood Glucose | 血糖 |
| GLU-U | Uglu Urine Glucose | 尿糖 |
| H | Hour | 小时 |
| Hb | Hemoglobin | 血红蛋白 |
| HDL-C | High-Density Lipoproteincholesterol | 血浆高密度脂蛋白胆固醇 |
| HIV | Human Immunodeficiency Virus | 人类免疫缺陷病毒 |
| IB | Investigator’s Brochure | 研究者手册 |
| IC_50_ | Half Maximal Inhibitory Concentration | 50%抑制浓度 |
| IRC | Independent Review Committee | 独立评审委员会 |
| irAE | Immune Related Adverse Events | 免疫相关不良事件 |
| irORR | Immune Related Objective Response Rate | 免疫相关的客观缓解率 |
| irRECIST | Immune Related Response Evaluation Criteria In Solid Tumors | 免疫相关的实体瘤评价标准 |
| IU | International Unit | 国际单位 |
| LEU | Leukocytes In Urine | 尿白细胞 |
| K^+^ | Serum Potassium | 血钾 |
| KET | Urine Acetone Bodies | 尿酮体 |
| kg | Kilogram | 千克 |
| LDH | Lactate Dehydrogenase | 乳酸脱氢酶 |
| LDL-C | Low-Density Lipoprotein Cholesterol | 血浆低密度脂蛋白胆固醇 |
| LYMPH | Lymphocyte | 淋巴细胞 |
| Mcrc | Metastatic Colorectal Cancer | 转移性结直肠癌 |
| MFD | Maximum Feasible Dose | 最大可行剂量 |
| mg | Milligram | 毫克 |
| Min | Minutes | 分钟 |
| Ml | Milliliter | 毫升 |
| mm | Millimeter | 毫米 |
| MRI | Magnetic Resonance Imaging | 磁共振成像 |
| MSI | Microsatellite Instability | 微卫星不稳定性 |
| MTD | Maximum Tolerated Dose | 最大耐受药物剂量 |
| Na^+^ | Plasma Sodium | 血钠 |
| NCCN | National Comprehensive Cancer Network | 美国国立综合癌症网络 |
| NCI-CTC | National Cancer Institute Common Terminology Criteria | 国家肿瘤研究所通用毒性标准 |
| NEUT | Neutrophil | 中性粒细胞 |
| NMPA | National Medical Products Administration | 国家药品监督管理局 |
| NYHA | New York Heart Association | 纽约心脏协会 |
| NOAEL | No-Observed-Adverse-Effect Level | 无可见有害作用水平 |
| OBD | Optimal Biological Dose | 最适生物剂量 |
| ORR | Objective Response Rate | 客观缓解率 |
| OS | Overall Survival | 总生存期 |
| PACAP | Pituitary Adenylate Cyclase Activating Polypeptide | 垂体腺苷环化酶激活多肽 |
| PD | Progressive Disease | 疾病进展 |
| PD-1 | Programmed Cell Death Protein 1 | 程序性死亡受体-1 |
| PD-L1 | Programmed Death-Ligand 1 | 程序性死亡配体-1 |
| PFS | Progression Free Survival | 无进展生存期 |
| PI | Principal Investigator | 主要研究者 |
| PK | Pharmacokinetics | 药代动力学 |
| PLT | Blood Platelet | 血小板 |
| Pmmr | Mismatch Repair Proficient | 无错配修复基因缺失 |
| PRO | Protein In Urine | 尿蛋白 |
| PT | Prothrombin Time | 凝血酶原时间 |
| RBC | Red Blood Cell Count | 红细胞计数 |
| RECIST | Response Evaluation Criteria In Solid Tumors | 实体瘤疗效评价标准 |
| SAE | Serious Adverse Event | 严重不良事件 |
| SAP | Statistical Analysis Plan | 统计分析计划 |
| SD | Stable Disease | 疾病稳定 |
| SDV | Source Data Verification | 原始数据核查 |
| sec | Second | 秒 |
| SIE | Special Interest Event | 特别关注的不良事件 |
| Sua | Serum Uric Acid | 血尿酸 |
| T-BIL | Total Bilirubin | 总胆红素 |
| TC | Total Cholesterol | 总胆固醇 |
| TG | Triglyceride | 甘油三酯 |
| TGF-β | Transform Growth Factor-Β Receptor Type II | 转化生长因子-Βii型受体 |
| TSH | Thyroid Stimulating Hormone | 促甲状腺激素 |
| UA | Uric Acid | 尿酸 |
| UBIL | Urine Bilirubin | 尿胆红素 |
| ULN | Upper Limit Of Normal | 正常值上限 |
| URBC | Urine Red Blood Cell | 尿红细胞 |
| VEGF | Vascular Endothelial Growth Factor | 血管内皮生长因子 |
| WBC | White Blood Cell Count | 白细胞计数 |

# Introduction

## Study background

### Epidemiology and treatment status of colorectal cancer

Colorectal cancer (CRC) is one of the most common malignant tumors in the world, which seriously threatens human health. CRC ranks the third in incidence and the second in mortality, only second to lung cancer^[1]^. China is one of the high-prevalence areas of colorectal cancer, with nearly 400,000 new cases of colorectal cancer every year, and the trend is increasing year by year^[2]^. Early colorectal cancer is mainly treated by surgery, combined with chemotherapy and radiotherapy. However, due to the lack of specific clinical manifestations of early colorectal cancer, a considerable proportion of patients are already at the locally advanced stage or have distant metastasis at the time of initial diagnosis, thus losing the opportunity for surgery. At present, chemotherapy combined with targeted therapy is the main treatment for advanced colorectal cancer.

In the guidelines of the National Comprehensive Cancer Network (NCCN), FOLFOX (fluorouracil, oxaliplatin and calcium folinate), FOLFIRI (fluorouracil, irinotecan and calcium folinate) or XELOX (capecitabine, oxaliplatin) combined with or without targeted therapy are recommended as the first-line therapy for metastatic colorectal cancer. Bevacizumab is the most widely used targeted therapy for the treatment of colorectal cancer currently.

Bevacizumab is a humanized monoclonal antibody against vascular endothelial growth factor (VEGF), and one of the most commonly used targeted therapies in the treatment of advanced colorectal cancer. A number of Phase 3 clinical studies have confirmed that chemotherapy combined with bevacizumab is superior to chemotherapy alone. Bevacizumab was approved by the U.S. Food and Drug Administration (FDA) and the National Medical Product Administration (NMPA, China) respectively in 2004 and 2010, for the treatment of patients with metastatic colorectal cancer in combination with fluorouracil-based chemotherapy.

According to the available data, when receiving the first-line standard of care for metastatic colorectal cancer, less than 50% of the patients have response to treatment, and median PFS is only 10 months, median OS is only about 20 months, five-year survival rate is only about 12%, thus a more effective and safe treatment regimen is urgently required clinically^[3]^.

### Study progress of immunotherapy in the first-line treatment of colorectal cancer

Immunotherapies, especially immune checkpoint inhibitors, have developed rapidly in recent years, becoming the fourth most important tumor treatment method after surgery, radiotherapy and chemotherapy. Antibodies targeting programmed death receptor-1 (PD-1) and programmed death ligand-1(PD-L1) are the main immunotherapy drugs under study at present. Anti-PD-1/PD-L1 antibodies regulate the anti-tumor activity of T cells by blocking the mutual binding of PD-1 and PD-L1 to maximize the response of patients' own immune system to attack tumor cells, thus achieving the purpose of killing tumor cells^[4]^.

Breakthrough has been made in immunotherapies for treating MSI-H/dMMR (microsatellite instability-high/MIS-match repair deficiency) colorectal cancer, and several immunotherapy drugs have been approved by FDA for first-line and subsequent-line treatment of this kind of patients^[5]^. However, MSI-H/dMMR colorectal cancer accounts for only 5% of patients with advanced colorectal cancer. Most patients are having colorectal cancer of the microsatellite stability (MSS) type, and no immunotherapy drugs have been approved for this population.

At present, there are some exploratory study data reported on immunotherapies for the first-line treatment of MSS colorectal cancer. In a study on the first-line treatment of colorectal cancer with PD-1 monoclonal antibody Pembrolizumab combined with FOLFOX regimen, 30 subjects were enrolled, and the ORR was up to 53% and DCR was 100%^[6]^; in another study of PD-L1 monoclonal antibody Durvalumab and CTLA-4 monoclonal antibody Tremelimumab combined with FOLFOX, ORR was up to 62.5% and DCR was up to 87.5%^[7]^, demonstrating preliminary efficacy.

In an exploratory study (SHR-1210-210) on the first-line treatment of colorectal cancer with PD-1 monoclonal antibody Camrelizumab combined with BP102 (a biosimilar of Bevacizumab) and XELOX, as of November 24, 2020, 12 patients with MSS colorectal cancer were included, with ORR up to 66.7%, DCR 100%, median PFS 9.75 months, maximum PFS up to 22.4 months, and 4 (33.3%) subjects had a PFS of more than 22 months, suggesting that patients with MSS colorectal cancer could benefit from immunotherapy combined with the standard first-line treatment when compared with previous study data.

### Anti-PD-L1/TGF-β bifunctional immunotherapy

Although several exploratory studies have shown that immunotherapy may play a role in the first-line treatment of MSS colorectal cancer, the study progress is slow. MSS colorectal cancer is considered as a "cold tumor" insensitive to immunotherapy, and how to overcome the primary drug resistance against immunotherapy is an urgent problem to be solved at present^[4]^.

Recent studies have shown that TGF-β signaling pathway also plays a very important role in immune escape of colorectal cancer. The progression of colorectal cancer has a clear correlation with the successive changes in WNT, EGFR, P53 and TGF-β signaling pathways, and the change in TGF-β signaling pathway is a sign that colorectal cancer enters the advanced stage. TGF-β can up-regulate the expression of PD-L1 on antigen presenting cells (APC) which can release soluble PD-L1 into tumor microenvironment, and directly inhibit cytotoxic T lymphocytes or induce cytotoxic T lymphocytes to release inhibitory soluble molecules through the interaction between PD-L1 and its receptor. Therefore, targeting PD-L1 and TGF-β simultaneously can restore the activity of T cells, enhance the immune response, and more effectively improve the effect of inhibiting tumor occurrence and development^[8]^.

Bifunctional fusion protein is a fusion protein with two functional domains. Because of its specificity and bifunctionality, it has become a hot study topic in the field of bioengineering drugs, with promising application prospects in the fields of tumor immunotherapy and treatment of autoimmune diseases. M-7824, jointly developed by EMD Serono Inc and Merck KGaA, is the first bifunctional fusion protein against PD-L1 and TGF-β entering clinical stage. It has shown preliminary efficacy against several tumors, and has now entered the Phase 3 clinical study stage. Exploratory studies have also been conducted in the treatment of colorectal cancer with M7824, and 32 patients with MSS colorectal cancer who have received at least three-line treatment were enrolled to receive M7824 monotherapy, and among the 29 evaluable patients, 1 achieved PR and 1 achieved SD^[9]^.

SHR-1701 is an anti-PD-L1/TGF-βRII bifunctional fusion protein developed by Hengrui. Its molecular form consists of two moieties: the anti-PD-L1 monoclonal antibody, and the truncated extracellular domain of TGF-βRII. Specifically, it is formed by connecting the C-terminal amino acid of the heavy chain of anti-PD-L1 antibody with the N-terminal amino acid of TGF-β RII extracellular domain through the linker protein fragment (G4S) 4G. SHR-1701 can block PD-L1 pathway and neutralize TGF-β in tumor microenvironment. The co-inhibition of PD-L1 and TGF-β signals can induce more effective anti-tumor immune response than single inhibition of either pathway, thus achieving the purpose of enhancing anti-tumor efficacy.

The preclinical study data have demonstrated that in the mouse subcutaneous xenograft (colon cancer cell MC38/H11) model, SHR-1701 showed an anti-tumor activity significantly superior to PD-L1 monoclonal antibody. In a previous Phase 1 clinical study of SHR-1701, 5 patients with colorectal cancer were included, among whom 1 patient with MSI-H was evaluated as PR and the other 4 patients with MSS were evaluated as PD.

According to the study data of immunotherapies in the first-line and last-line treatment of colorectal cancer, the efficacy of immunotherapy alone in the treatment of advanced colorectal cancer was very limited, whereas the combination with standard of care may bring a breakthrough.

The objective of this clinical study is to explore the efficacy and safety of SHR-1701 combined with BP102 (a biosimilar of Bevacizumab) and XELOX vs. placebo combined with BP102 and XELOX in the first-line treatment of patients with unresectable recurrent or distantly metastatic colorectal cancer, which is expected to bring better survival benefits to majority of patients with colorectal cancer.

This clinical study should be conducted according to the study protocol, Good Clinical Practice (GCP) and relevant laws and regulations.

## Scientific basis

### Study design basis

SHR-1701 can block PD-1/PD-L1 pathway and neutralize TGF-β in tumor microenvironment. The co-inhibition of PD-L1 and TGF-β signals can induce more effective anti-tumor immune response than single inhibition of either pathway, thus achieving the purpose of enhancing anti-tumor efficacy.

In the standard of care of colorectal cancer, chemotherapy drugs can directly kill tumor cells through cytotoxicity, increase the antigen exposure of tumor cells and promote immune presentation, while reducing myeloid-derived suppressor cells (MDSCs) and increasing the ratio of cytotoxic lymphocytes to regulatory T cells, relieving the immunosuppression effect caused by regulatory T cells and increasing the activity of T cells and the identifiability of tumor cells^[10]^. Immunotherapy combined with chemotherapy has been approved for several tumor species, such as the first-line treatment of non-squamous non-small cell lung cancer with PD-1 monoclonal antibody pembrolizumab combined with pemetrexed and cisplatin, and the treatment of PD-L1 positive triple-negative breast cancer with PD-L1 monoclonal antibody atezolizumab combined with albumin paclitaxel.

Bevacizumab is an anti-angiogenic drug, and previous studies have shown that it can reduce the activity of MDSCs and regulatory T cells, so that T cells bound to tumor antigens can be initiated and activated more effectively. Meanwhile, as an ideal combination drug for immunotherapy, it can normalize tumor angioarchitecture and promote T cells to enter tumors^[11]^. Avelumab combined with axitinib or pembrolizumab combined with lenvatinib for the first-line treatment of advanced renal cell carcinoma has been recognized by FDA as breakthrough drug therapy. Meanwhile, atezolizumab combined with Bevacizumab and chemotherapy for the first-line treatment of non-small cell lung cancer and atezolizumab combined with Bevacizumab for the first-line treatment of liver cancer have been approved by FDA, demonstrating the synergistic effect of immunotherapy when combined with chemotherapy and anti-angiogenic drug therapy.

Therefore, SHR-1701 combined with anti-angiogenesis drug BP102 and chemotherapy is an ideal combination strategy for the first-line treatment of colorectal cancer. At present, randomized controlled studies on first-line treatment of advanced colorectal cancer with immune checkpoint inhibitors Nivolumab, HLX10, Atezolizumab and Sintilimab have been conducted (see Table 1), and the combined use of immune checkpoint inhibitors, chemotherapy and anti-angiogenic drugs may provide a new treatment regimen for the first-line treatment of advanced colorectal cancer.

Table 1 Randomized controlled studies on first-line treatment of advanced colorectal cancer

| Study name/NCT number | Indications | Study design | Sample size | Primary endpoint | Start date |
| --- | --- | --- | --- | --- | --- |
| CheckMate 9X8/  NCT03414983 | MSS mCRC | Nivolumab+FOLFOX+Bevacizumab vs FOLFOX+Bevacizumab | 180 | PFS | 2018.2.14 |
| IBI201910/  NCT04194359 | RAS mutant mCRC | Sintilimab+XELOX+Bevacizumab vs XELOX+Bevacizumab | 436 | PFS | 2020.6 |
| AtezoTRIBE/  NCT03721653 | mCRC | Atezolizumab+FOLFOXIRI+Bevacizumab vs FOLFOXIRI+Bev | 201 | PFS | 2018.11.30 |
| NCT04547166 | mCRC | HLX10+HLX04+XELOX vs Placebo+Bevacizumab+XELOX | 666 | PFS | 2020.9.18 |

### Drug development rationale

SHR-1701 is an anti-PD-L1/TGF-βRII bifunctional fusion protein developed by Hengrui. Its molecular form consists of two moieties: the anti-PD-L1 monoclonal antibody, and the truncated extracellular domain of TGF-βRII. Specifically, it is formed by connecting the C-terminal amino acid of the heavy chain of anti-PD-L1 antibody with the N-terminal amino acid of TGF-β RII extracellular domain through the linker protein fragment (G4S) 4G. SHR-1701 can block PD-1/PD-L1 pathway and neutralize TGF-β in tumor microenvironment. The co-inhibition of PD-L1 and TGF-β negative signals can induce more effective anti-tumor immune response than single inhibition of either pathway, thus achieving the purpose of enhancing anti-tumor efficacy.

Shanghai Hengrui Pharmaceutical Co., Ltd. and Jiangsu Hengrui Pharmaceutical Co., Ltd. have developed BP102 (a recombinant humanized monoclonal antibody injection against VEGF), a biosimilar of Avastin^®^. In all the pre-clinical studies, comparison was made between BP102 and Avastin^®^, and the pharmaceutical, pharmacological and toxicological tests demonstrated that BP102 was similar to the reference drug Avastin^®^. Previous studies have demonstrated that our product is similar to the original drug, supporting the clinical efficacy comparison test. At present, a randomized, controlled and double-blind Phase 3 clinical study of non-squamous non-small cell lung cancer has been completed in more than 40 study sites in China, and relevant data have been included in the NDA submission.

#### SHR1701 study data

##### Name, pharmaceutical characteristics and dosage form of SHR-1701

[Generic name]: SHR-1701 injection

[Development codes]: SHR-1701, HR301507, HRP00240

[English name]: SHR-1701 Injection

[Molecular weight]: about 175 k Daltons (Da.)

[Dosage form]: Injection

[Strength]: According to the need of clinical study, the strength of this product is tentatively determined to be 6 mL: 0.3 g.

[Physical and chemical properties]: Colorless to yellowish clear liquid, slightly opalescent, without visible particles.

[API, excipients]: The active ingredient of this product is SHR-1701 bifunctional fusion protein (anti-PD-L1/TGF-βRII bifunctional fusion protein), and the excipients include polysorbate 80, sucrose, citric acid, sodium citrate and water for injection.

[Storage]: This product should be protected from light, and stored and transported in original package at 2℃-8℃. Avoid freeze and thaw and shaking.

[Shelf life]: 24 months tentatively

##### Pharmacological type and mechanism of action

Programmed death 1 ligand 1 (PD-L1), also known as B7 homologue 1 (B7-H1), is a type I transmembrane protein of B7 family. It is usually expressed on antigen presenting cells (APCs), and mainly binds to programmed cell death 1 (PD-1; CD279) and releases inhibitory signals to limit the activation and expansion of T cells, thus playing an important role in down-regulating autoimmunity. In recent years, it has been found that PD-L1 is highly expressed on the surface of multiple tumor cells. PD-L1 inhibits the function of lymphocytes and mediates the immune escape of tumors by binding to PD-1 molecules on the surface of tumor infiltrating lymphocytes. Inhibitors targeting PD-1/PD-L1 pathway can relieve the immunosuppressive effect mediated by PD-L1, enhance the function of killer T cells, and mobilize the immune system to eliminate tumor cells in vivo.

Transforming growth factor -β (TGF-β) is a cytokine with various biological activities, which is involved in the regulation of cell proliferation, differentiation, development and apoptosis. TGF-β has opposite dual functions for tumor cells at different stages. In the early stage of tumorigenesis, TGF-β can promote tumor cell cycle arrest and cause apoptosis; whereas in the late stage of tumorigenesis, it can promote the migration and infiltration of tumor cells, thus promoting the progress and metastasis of cancer. TGF-β has three subtypes in mammals: TGF-β1, TGF-β2 and TGF-β3, which play a regulatory role by binding to cell surface receptors. TGF-βRII can directly bind to the free ligand TGF-β1 or TGF-β3, and initiate the whole signaling pathway. The TGF-βRII bound to the ligand connects and phosphorylates the type I receptor TGF-βRI, and further phosphorylates the downstream substrate Smad protein. Therefore, TGF-βRII plays a vital role in the above signaling pathway, and it is the central link for TGF-β to play its regulatory role.

TGF-β can up-regulate PD-L1 on antigen presenting cells (APC) which can release soluble PD-L1 into tumor microenvironment, and directly inhibit cytotoxic T lymphocytes or induce cytotoxic T lymphocytes to release inhibitory soluble molecules through the interaction between PD-L1 and its receptor. Therefore, on the basis of inhibiting PD-1/PD-L1 pathway, targeting and neutralizing TGF-β in tumor microenvironment can restore the activity of T cells, enhance immune response, and more effectively improve the effect of inhibiting tumor occurrence and development, thus becoming a new choice for tumor treatment.

##### Preclinical pharmacology

Biacore binding test has shown that SHR-1701 has strong affinity with human PD-L1 protein (10-10M) and with TGF-β1 of human and mice (10-12~10-13M), and shows good selectivity for three different subtypes of human TGF-β, comparable to the control antibody M7824. SHR-1701 can bind to human and monkey PD-L1 protein, but does not significantly bind to mouse PD-L1 and other human B7 family proteins, suggesting that the binding of SHR-1701 to human PD-L1 is species-selective and specific. Further in vitro studies have shown that SHR-1701 can bind to cells with high expression of human PD-L1 (MC-38/H-11 mouse colon cancer cells, with knockout of murine PD-L1 and stable transfection of human PD-L1), block PD-1/PD-L1 signaling pathway and inhibit the phosphorylation of smad3 downstream of TGF-β. SHR-1701 can also promote the proliferation of human PBMC and induce the release of IFN-γ. As an antibody of IgG4 subtype, SHR-1701 has no significant ADCC and CDC effects. The study on anti-tumor activity of SHR-1701 in vivo has shown that the injection of SHR-1701(3, 10, 30 mg/kg, t.i.w.×8) via tail vein significantly inhibited the growth of subcutaneous xenograft in MC38/H-11 mice, at an effective dose of 10 mg/kg. The inhibition effect is to a certain extent dose-dependent, but varies greatly among individuals. Tumor-bearing mice can well tolerate the test substance. At the same dose, the inhibitory effect of SHR-1701 on the subcutaneous xenograft in MC38/H-11 mice were superior to that of the control antibody SHR-1316 (P<0.05).

##### Safety pharmacology

Single intravenous administration of 30, 100 and 300 mg/kg of SHR-1701 had no significant effect on the central nervous system function of SD rats, and single intravenous infusion of 100 mg/kg of SHR-1701 had no significant effect on the cardiovascular and respiratory functions and body temperature of awake and unconstrained cynomolgus monkeys. The tissue cross-reaction test conducted with fresh frozen normal human tissues, cynomolgus monkey tissues and rat tissues showed that SHR-1701 had tissue cross-reaction with human placenta and tonsil, cynomolgus monkey tonsil, mammary gland, parotid gland, and striated muscle, as well as rat choroid plexus and parotid gland, but had no cross-reaction with other tissues of the three species.

##### Animal pharmacokinetics and metabolism

The pharmacokinetics of single and multiple doses were studied in SD rats and cynomolgus monkeys in non-clinical pharmacokinetic studies, and the immunogenicity was tested. In addition, in the study of pharmacokinetics in cynomolgus monkeys, the receptor occupancy (RO) on T cells was also studied. Tissue distribution and excretion of 125I labeled SHR-1701 were studied in C57 mice and SD rats, respectively. After a single intravenous injection of SHR-1701 at 3, 10 and 30 mg/kg in SD rats, there was no significant sex difference in the exposure of SHR-1701 in each dose group. The exposure of SHR-1701 in SD rats increased with dose, and C_max_ and AUC increased linearly with dose. SHR-1701 showed certain immunogenicity in rats, and the proportion of ADA positive animals in low, medium and high dose groups was 16.7% (1/6), 16.7% (1/6) and 66.7% (4/6) respectively. After a single intravenous injection of SHR-1701 at 3, 10 and 30 mg/kg in cynomolgus monkeys, there was no significant sex difference in the exposure of SHR-1701 in each group. The exposure of SHR-1701 in cynomolgus monkeys increased with dose, and C_max_ and AUC increased linearly with dose. After repeated intravenous injection of 10 mg/kg of SHR-1701 for injection in cynomolgus monkeys, once a week for four consecutive weeks, there was no significant in vivo accumulation of SHR-1701. SHR-1701 showed strong immunogenicity in cynomolgus monkeys, and all animals in each group were ADA positive after administration. After a single intravenous infusion of SHR-1701 in cynomolgus monkeys, it was observed that the receptor occupancy level on the surface of T cells was saturated quickly, and the receptor occupancy in the 3 and 10 mg/kg dose groups gradually decreased over time. After intravenous injection of 125I labeled SHR-1701 in tumor-bearing mice, the radioactivity was high in tissues and organs with rich blood perfusion, for example, for heart, lung and kidney, the radioactivity intensity was high in tumor tissues, suggesting that the drug distribution is tumor targeting; however, the radioactivity was less distributed in organs with poor blood perfusion. SHR-1701 was mainly excreted by urine, and the excreta in urine was small molecular degradation product, without any unchanged drug, and a small amount was excreted by feces. The excretion by urine and feces accounted for 56.21% ± 7.37% and 29.43% ± 5.57% of the injected radioactivity respectively.

##### Toxicological studies

All non-clinical toxicological studies followed the GLP specifications to ensure that the study results could truly and accurately reflect the toxicity of this product. Single intravenous administration of 30, 100 and 300 mg/kg of SHR-1701 had no significant effect on the central nervous system function of SD rats, and single intravenous infusion of 100 mg/kg of SHR-1701 had no significant effect on the cardiovascular and respiratory functions and body temperature of awake and unconstrained cynomolgus monkeys. The tissue cross-reaction test conducted with fresh frozen normal human tissues, cynomolgus monkey tissues and rat tissues showed that SHR-1701 had tissue cross-reaction with human placenta and tonsil, cynomolgus monkey tonsil, mammary gland, parotid gland, and striated muscle, as well as rat choroid plexus and parotid gland, but had no cross-reaction with other tissues of the three species. After a single intravenous infusion of 30 mg/kg and 750 mg/kg SHR-1701 in SD rats, no abnormal reaction was observed, and the maximum tolerated dose (MTD) was ≥ 750 mg/kg; after a single intravenous infusion of 150 mg/kg and 479 mg/kg SHR-1701 in cynomolgus monkeys, only one animal in the high-dose group was found to have a transient increase in WBC, Neut and Retic, which was considered to be related to the test article, and the MTD was ≥ 479 mg/kg. Repeated toxicity tests of this product were conducted in rats and cynomolgus monkeys to evaluate the toxicity of SHR-1701 after repeated administration. SHR-1701 was given to SD rats by repeated intravenous infusion at doses of 15, 50 and 150 mg/kg, once a week for 4 consecutive weeks, with a total of 5 doses. The decrease of TGF-β1 related to pharmacological action was observed in all dose groups; the weight of thymus decreased in each dose group, and histological examination showed minimal or mild decrease of cortical lymphocytes in thymus. SHR-1701 showed certain immunogenicity in rats, and the proportion of ADA positive animals in low, medium and high dose groups was 25% (2/8), 12.5% (1/8) and 12.5% (1/8) respectively. Under the condition of this study, the dose severely toxic to 10% of the animals (STD10) in SHR-1701 group was > 150 mg/kg. At this dose, the mean C_max_ and AUC_(0-144h)_ of male animals on D29 were 6465.397 μg/mL and 344536.8 μg*hr/mL, respectively, while those of the female animals on D29 were 6303.623 μg/mL and 432659.5 μg*hr/mL, respectively. SHR-1701 was given to cynomolgus monkeys by repeated intravenous infusion at doses of 30, 75 and 150 mg/kg, once a week for 4 consecutive weeks, with a total of 5 doses. The animals in each dose group showed a transient minimal decrease of HGB related to pharmacological action, and no other significantly abnormal changes were seen in the general state, weight, food consumption, body temperature, lead II electrocardiogram, blood pressure, respiratory frequency, ophthalmologic examination, hematology, blood chemistry, urinalysis, bone marrow examination, complement, circulating immune complex, lymphocyte subsets, cytokines, organ weight and coefficient, gross necropsy observation and histopathological examination of cynomolgus monkeys in each group. SHR-1701 showed strong immunogenicity in cynomolgus monkeys, and the proportion of ADA positive animals in low, medium and high dose groups was 100% (10/10), 80% (8/10) and 100% (10/10) respectively. Under the condition of this study, the no observed adverse effect level (NOAEL) was 150 mg/kg. At this dose, the mean C_max_ and AUC_(0-144h)_ of male animals on D29 were 6327.359 μg/mL and 498550.820 μg*hr/mL, respectively, while those of the female animals on D29 were 5502.563 μg/mL and 416606.480 μg*hr/mL, respectively. The in vitro hemolysis test and local irritation test (accompanying the long-term toxicity study) showed negative results, indicating that SHR-1701 had no hemolysis effect or irritation to the injection site.

##### Clinical studies of SHR-1701

As of the safety data cut-off date (June 6, 2020), 4 clinical studies of SHR-1701 (study SHR-1701-I-101, study SHR-1701-I-102, study SHR-1701-I-103 and study SHR-1701-001AUS) have been conducted, and 160 subjects have received treatment with SHR-1701.

SHR-1701 is still in the early stage of clinical development. Among the 160 subjects who have received the treatment with SHR-1701, the adverse events (AEs) related to SHR-1701 with an incidence of ≥5% include: aspartate aminotransferase increased (11.3%), fatigue, rash (9.4% each), alanine aminotransferase increased (8.8%), anemia (8.1%), hypothyroidism, hyperthyroidism, gingival bleeding (7.5% each), anorexia (6.9%), combined bilirubin increased, serum bilirubin increased (5.6% each), proteinuria and fever (5% each).

In the clinical studies of SHR-1701, serious adverse event (SAE) occurring in ≥2 subjects included progressive tumor (11 cases, 6.9%), death (7 cases, 4.4%), anemia (4 cases, 2.5%), vomiting, fever (3 cases each, 1.9%), liver failure, elevated serum creatinine, immune-mediated pneumonia, and gastrointestinal bleeding (2 cases each, 1.3%). Among them, drug-related (including undetermined) SAEs included fever, vomiting (3 cases each, 1.9%), immune-mediated pneumonia, and anemia (2 cases each, 1.3%), gastrointestinal bleeding, death, hypophysitis, fatigue, sudden death, decreased platelet count, hypokalemia, increased serum creatinine, hypoalbuminemia, increased alanine aminotransferase, and hemoptysis (1 case each, 0.6%).

Fatal AEs in clinical studies of SHR-1701 included progressive tumor (11 cases, 6.9%), death (7 cases, 4.4%), liver failure (2 cases, 1.3%), gastrointestinal bleeding, cerebral infarction, and sudden death (1 case each, 0.6%). Among them, there was 1 unexplained death in the study of SHR-1701-I-101, which may be related to liver failure (progressive disease) and the investigational drug in the opinion of the investigator. In addition, there was 1 case of sudden death in the study of SHR-1701-I-102. Since the subject died suddenly during the trip, the investigator could not determine the correlation with the investigational drug.

Other pharmacological studies, pharmacokinetic tests, toxicology tests, clinical study information and related charts are detailed in the Investigator's Brochure.

##### Progress of clinical studies of other similar drugs

A number of international pharmaceutical companies are developing humanized monoclonal antibodies against PD-L1, which can maximize the patients' immune system response against tumors by blocking the binding of PD-L1/PD-1, thus achieving the purpose of killing tumor cells. Atezolizumab (trade name: Tecentriq) manufactured by Roche is the first fully humanized monoclonal antibody against PD-L1 marketed globally, which has been approved by FDA for the treatment of advanced bladder cancer, non-small cell lung cancer and urothelial cancer. Bavencio (avelumab), a PD-L1 inhibitor jointly developed by Merck and Pfizer, was granted the accelerated approval by the US FDA in April 2017 for the treatment of metastatic Merkel cell carcinoma in adults and children over 12 years old, and it is also applicable to patients without previous chemotherapy. Bavencio is the first drug approved by FDA to treat Merkel cell carcinoma and the second PD-L1 inhibitor approved by FDA. In May 2017, the US FDA granted the accelerated approval for the PD-L1 antibody Durvalumab (trade name: Imfinzi) manufactured by AstraZeneca, for the treatment of advanced bladder cancer. Concurrently approved was a PD-L1 detection kit -SP263, which was used to detect the expression of PD-L1. So far, there are five kinds of PD-1/PD-L1 antibody drugs on the market, including PD-1 antibody Pembrolizumab manufactured by MSD, PD-1 antibody Nivolumab manufactured by BMS, PD-L1 antibody Atezolizumab manufactured by Roche, PD-L1 antibody Avelumab manufactured by Pfizer/Merck, PD-L1 antibody Durvalumab manufactured by AstraZeneca, PD-1 monoclonal antibody Toripalimab manufactured by TopAlliance, PD-1 monoclobal antibody Sintilimab manufactured by Innovent, PD-1 monoclonal antibody Tislelizumab manufactured by BeiGene, and PD-1 antibody Camrelizumab manufactured by Hengrui.

At present, there has been no TGF-β inhibitor on the market. In recent years, a series of methods to inhibit the expression of TGF-β and its receptor have been used in animal studies and Phase 1 clinical studies, including monoclonal antibody technology against TGF-β, small kinase inhibitors that inhibit TGF-β signaling pathway, antisense RNA or RNA interference strategy, tumor vaccine, etc. to achieve the purpose of treating tumors. Fresolimumab (GC1008) is a humanized IgG4 monoclonal antibody, which can neutralize TGF-β 1, 2, and 3 in tumor microenvironment. At present, it is developed to treat idiopathic pulmonary fibrosis, focal segmental glomerulosclerosis and malignant tumors. Galunisertib (LY2157299) is a small molecular inhibitor against TGFβR1 kinase developed by Lilly, which can selectively block TGF-β signal in vitro, indicated for glioma, liver cancer, pancreatic cancer and myelodysplastic syndrome, etc. Preliminary studies have shown that LY2157299 has anti-tumor activity in different hepatocellular carcinoma models, and it has been demonstrated that it has good toxicity profile in patients with glioma. Bristol-Myers Squibb and Eli Lilly reached an agreement in early 2015 for cooperation in clinical studies, and the immunotherapy Opdivo (nivolumab) of the former was combined with the galunisertib (LY2157299) of the latter. Their safety and efficacy in patients with advanced glioblastoma, hepatocellular carcinoma and non-small cell lung cancer were observed, and it was believed that the co-inhibition effect against negative signals of PD-1 and TGF-β could induce more effective anti-tumor immune response than single inhibition of either pathway.

MSB0011359C (M-7824) jointly developed by EMD Serono Inc and Merck KGaA is the only anti-PD-L1/TGF-βRII bifunctional fusion protein under clinical study globally, which is used to treat advanced solid tumors and other indications. An open-label, dose-escalating Phase 1 clinical study (NCT02517398) investigated the safety and preliminary efficacy of M7824 in patients with advanced solid tumors. From September 2015 to March 2017, a total of 19 subjects participated in this study. These subjects received M7824 at a dose of 0.3-20 mg/kg, including 3 subjects each at 0.3 mg/kg, 1 mg/kg, 3 mg/kg and 10 mg/kg, and 7 subjects at 20 mg/kg. The median duration of treatment was 11.9 weeks (4.0-41.9 weeks). 47% of the subjects reported ≥ Grade 1 drug-related adverse events. Four patients reported ≥ Grade 3 drug-related adverse events including skin infection secondary to local bullous pemphigoid (3 mg/kg), asymptomatic lipase increased (20 mg/kg), colitis with anemia (20 mg/kg), and gastroparesis with hypokalemia (10 mg/kg), in 1 subject each. Up to the maximum dose level of 20 mg/kg, the maximum tolerable toxicity has not been achieved. No subject died due to adverse events. The efficacy analysis of this study showed that anti-tumor effect was observed at all dose levels (Figure 1). Among them, 1 subject had complete response (cervical cancer), 2 subjects had continuous partial response (pancreatic cancer and anal cancer), and 1 subject with cervical cancer had nearly partial response, and 2 subjects had continuous stable disease (pancreatic cancer and bronchopulmonary carcinoid).

Figure 1 Assessment of efficacy of M7824 in Phase 1 clinical study

Overall, M7824 has controllable safety while demonstrating an encouraging anti-tumor effect.

#### BP102 study data

##### Drug name and physical and chemical properties of BP102

[Generic name]: Bevacizumab Injection

[English name]: Bevacizumab Injection

[Development code]: BP102

##### Pharmacology and mechanism of action

Bevacizumab is a synthesized recombinant humanized IgG1 monoclonal antibody against VEGF. Bevacizumab can inhibit the binding of vascular endothelial growth factor (VEGF) to its receptors (FLT-1 and KDR) on endothelial cells, so that VEGF cannot play its role in promoting the proliferation of vascular endothelial cells and angiogenesis in tumors, thus blocking the supply of blood, oxygen and other nutrients essential for tumor growth, preventing the tumors from growing and spreading in vivo, and enabling chemotherapy to play an effective role in tumors, so as to delay tumor growth and metastasis.

##### Pre-clinical study results of BP102

The pharmacodynamic studies of BP102 were classified into in vitro and in vivo studies to observe the selectivity, action intensity and mechanism of the drug to the target; the anti-tumor effect of the drug in vivo was observed through the efficacy test in nude mice with different human xenografts; In addition, in vitro studies were classified into affinity and activity tests to investigate the binding and inhibition effects of the drug on targets. In all studies, Avastin^®^ was used as the control to investigate the difference between BP102 and Avastin^®^. BP102 showed good affinity to targets in vitro, as well as good anti-tumor activity at cellular level and in vivo in animals. Its affinity and pharmacological activity were highly consistent with those of the original drug Avastin^®^.

The chronic toxicity, acute toxicity, general pharmacology, immunogenicity and special safety of BP102 were fully investigated. The results of pharmacokinetic study showed that the pharmacokinetic profile of BP102 in cynomolgus monkeys was consistent with that of the control drug Avastin^®^; the toxicological results showed that the toxic reactions and target organs of BP102 in animals were consistent with those of the control drug Avastin^®^.

Pharmaceutical, pharmacological and toxicological studies showed that BP102 was similar to the reference drug Avastin^®^, which supported the pharmacokinetic comparison test and clinical safety and efficacy comparison test based on the assumption of biosimilars.

##### Study on bioequivalence of BP102 to Avastin^®^

The comparative study on pharmacokinetics of Bevacizumab (BP102) injection in humans was carried out in the Phase 1 clinical trial laboratory of the First Hospital of Jilin University. This study was a single-center, randomized, double-blind, single-dose, parallel pharmacokinetic comparison study in fasting condition among healthy adult male subjects, and 72 subjects were enrolled to evaluate the similarity between BP102 injection and Avastin^®^ in pharmacokinetics, safety and immunogenicity in healthy male volunteers. In October 2017, this pharmacokinetic comparison study was completed, and all the subjects finished the study. At present, data are being collected for statistical analysis. There are no new reports of serious drug-related adverse reactions.

Preliminary analysis of the available PK data showed that the ratios of log-transformed geometric mean values of AUC_0-t_, AUC_0-∞_ and C_max_ between the test product and reference product Bevacizumab were 100.70%, 101.20% and 100.59%, respectively. The corresponding 90% confidence intervals of the ratios were: 93.57%~108.37% (AUC_0-t_), 93.65%~109.37% (AUC_0-∞_) and 95.80%~105.62% (C_max_). The inter-individual variability of C_max_, AUC_0-t_ and AUC_0-∞_ was 12.1%, 18.27% and 19.39% respectively. The power values were 0.975 (AUC_0-t_), 0.999 (AUC_0-∞_) and 1.000 (C_max_), respectively. According to the nonparametric test, there was no significant difference in T_max_ of Bevacizumab between the test product and reference product. According to the bioequivalence criteria, it could be considered that the PK profiles of BP102 and Avastin have biosimilarity.

##### Phase 1 safety data of BP102

As of June 2, 2018, all the subjects in the BP102 bioequivalence study have finished the study, with 72 subjects enrolled in total, 36 in BP102 group and 36 in Avastin group. At present, data are being collected for statistical analysis. The currently available data are preliminarily summarized as follows:

A total of 36 subjects were enrolled in BP102 group, among which 30 subjects experienced 98 episodes of adverse events (TEAEs), accounting for 83.3% (30/36); a total of 36 patients were enrolled in Avastin group, among which 28 subjects experienced 92 episodes of adverse events (TEAEs), accounting for 77.8% (28/36). In this study, there were no serious adverse events, no adverse events leading to withdrawal from the study, and no subject died due to adverse events.

As for the severity of adverse events, according to CTCAE 4.03 criteria, most of the adverse events (TEAEs) in BP102 group and Avastin group were of CTCAE Grade 1-2, and the incidences of adverse events (TEAE) above Grade 3 were 13.9% (5/36) and 2.8%(1/36) in BP102 group and Avastin group, respectively.

In BP102 group, the top 3 adverse events (TEAEs) occurring in SOC Investigations were as follows: 8 subjects had an increase in serum unconjugated bilirubin, 4 subjects had an increase in white blood cell count, and 4 subjects had an increase in blood bilirubin; adverse events (TEAEs) occurring in SOC Metabolism and nutrition disorders were as follows: 8 subjects experienced hypertriglyceridemia, and 4 subjects experienced hyperuricemia. In Avastin group, the top 3 adverse events (TEAEs) occurring in SOC Investigations were as follows: 7 subjects had an increase in alanine aminotransferase, 6 subjects had an increase in serum unconjugated bilirubin, and 6 subjects had an increase in blood bilirubin; adverse events (TEAEs) occurring in SOC Metabolism and nutrition disorders were as follows: 4 subjects experienced hypertriglyceridemia, and 2 subjects experienced hyperuricemia.

##### Phase 3 clinical study of BP102 in non-squamous non-small cell lung cancer patients

The Phase 3 clinical study of Bevacizumab (BP102) injection in non-squamous non-small cell lung cancer patients was conducted in more than 40 study sites in China, led by Professor Feng Jifeng from Jiangsu Cancer Hospital. This study was a randomized, double-blind and multi-center study, with a total of 512 subjects enrolled to investigate the similarity in efficacy, safety, immunogenicity and PK profile between BP102 combined with paclitaxel/carboplatin and Avastin® combined with paclitaxel/carboplatin in the first-line treatment of advanced or recurrent non-squamous non-small cell lung cancer. The enrollment of the study has been completed, and relevant data have been submitted for marketing application.

### Rationales for regimen design

BP102 is a biosimilar to Bevacizumab, a recombinant humanized monoclonal antibody, which is one of the most commonly used targeted therapies for the treatment of colorectal cancer. XELOX is widely used in clinical practice because of its easy administration and low toxicity. XELOX combined with Bevacizumab is the standard first-line treatment regimen recommended by both CSCO and NCCN guidelines.

SHR-1701 can block PD-1/PD-L1 pathway and neutralize TGF-β in tumor microenvironment. The co-inhibition of PD-1 and TGF-β signals can induce more effective anti-tumor immune response than single inhibition of either pathway, thus achieving the purpose of enhancing anti-tumor efficacy.

Based on the above theoretical basis, the use of Bevacizumab, an anti-angiogenic drug, can promote the infiltration of T cells and normalize tumor blood vessels. On the basis of blocking PD-1/PD-L1 signaling pathway, SHR-1701 can also remove the TGF-β in tumor microenvironment in a targeted manner, thus relieving the inhibition against immune cell activity in tumor microenvironment by immunosuppressive factors, enhancing immune response, and more effectively improving the inhibition of tumor occurrence and development. The combination of chemotherapy can further promote the exposure of tumor antigens and improve the efficacy of immunotherapy, which is an ideal combined strategy for the first-line treatment of colorectal cancer at present. The combined use of immune checkpoint inhibitors, chemotherapy and anti-angiogenic drugs may provide a new treatment regimen for the first-line treatment of advanced colorectal cancer.

### Rationale for dose selection

#### Rationale for dose selection of SHR1701

According to the pharmacokinetic data of clinical Phase 1 study, the clearance rate in 30 mg/kg dose group tended to decrease and the elimination half-life was prolonged. The exposure (AUC_inf_) in the 30 mg/kg q3w group was about 2.26 folds that in the 20mg/kg q3w group, showing a nonlinear increase trend, which may be due to the target-mediated drug disposal (TMDD) effect. When the receptor was saturated, the drug clearance rate decreased. The preliminary correlation analysis of dose, exposure (AUC_inf_, C_max_) and efficacy (ORR, DCR) showed that the efficacy (ORR and DCR) was positively correlated with the drug exposure level (AUC_inf_, C_max_) to a certain extent, and both ORR and DCR increased with the increase of exposure. The preliminary correlation analysis of dose, exposure (AUC_inf_, C_max_) and safety (≥Grade 3 TRAE) showed that the occurrence of ≥ Grade 3 TRAE was not significantly related to the drug exposure level (AUC_inf_ and C_max_). Furthermore, in the previous tolerance observation, no DLT event occurred in the 30 mg/kg q3w dose group, and the safety was good. Therefore, based on the tolerance, safety, PK/PD and preliminary E-R analysis, it was proposed to choose 30 mg/kg q3w as the recommended Phase 2 dose (RP2D) for the subsequent exploratory study, which was expected to further improve the efficacy.

#### Rationale for dose selection of BP102

BP102 is a biosimilar to Bevacizumab. With reference to the dosing regimen of Avastin^®^, the dose was set at 7.5mg/kg, with 21 days as a cycle.

#### Rationale for dose selection of chemotherapy

XELOX is a first-line chemotherapy regimen for colorectal cancer recommended by both CSCO and NCCN guidelines, which is commonly used in clinical practice. The dosage of XELOX in this study was selected mainly based on CSCO guidelines for diagnosis and treatment of colorectal cancer (2019 edition), specifically as follows:

Oxaliplatin, 130 mg/m^2^, intravenous infusion (at least 2 hours), D1, with 21 days as a cycle;

Capecitabine, 1000 mg/m^2^/time, oral administration, twice daily (within 30 min after meal) consecutively for two weeks in each cycle, followed by a week of rest (21 days as one cycle).

## Potential Risks and benefits to study population

### Known potential risks

SHR-1701 is still in the early stage of clinical development. Among the 160 subjects who have received the treatment with SHR-1701, the adverse events (AEs) related to SHR-1701 with an incidence of ≥5% include: aspartate aminotransferase increased, fatigue, rash, alanine aminotransferase increased, anemia, hypothyroidism, hyperthyroidism, gingival bleeding, anorexia, combined bilirubin increased, serum bilirubin increased, proteinuria and fever. Therefore, similar drug-related adverse events may occur in this study. In addition, any drug may induce unexpected or even serious adverse reactions, which pose potential risks.

SHR-1701 is an immune checkpoint inhibitor, and subjects may experience a temporary tumor outbreak (i.e. pseudoprogression) after taking such drugs. In this study, the subjects in the test group who meet the disease progression criteria of RECIST1.1 as demonstrated by imaging but are still clinically stable are allowed to receive further treatment of SHR-1701 combined with chemotherapy and BP102. Since the imaging detection cannot distinguish between pseudoprogression and real progression, subjects who have real disease progression may not be able to receive other anti-tumor therapies in a timely manner. Therefore, the investigator should fully inform the subjects of this risk, and decide whether subjects should continue the medication by comprehensively assessing the imaging results, aspiration biopsy results and clinical symptoms.

BP102 is a biosimilar to Bevacizumab, and its adverse reactions are similar to those of Avastin^®^. In previous studies, the incidence of BP102-related adverse events was 80.92%, and the TRAEs with an incidence of ≥ 5.0% included proteinuria, hypertension, decreased platelet count, decreased white blood cell count, anemia, decreased neutrophil count, fatigue and epistaxis, loss of appetite, nausea, increased blood pressure, hemoptysis, hypertriglyceridemia and vomiting, increased alanine aminotransferase and diarrhea, and increased aspartate aminotransferase.

XELOX is one of the most commonly used chemotherapy regimens for colorectal cancer. XELOX consists of capecitabine and oxaliplatin. The common capecitabine-related AEs include gastrointestinal reactions (such as diarrhea, nausea, vomiting, abdominal pain, stomatitis), hand-foot syndrome, fatigue, headache, etc; and the common oxaliplatin-related AEs include anemia, leukopenia, granulocytopenia, thrombocytopenia, nausea, vomiting, diarrhea, nervous system abnormalities (such as peripheral neuritis). Other AEs related to capecitabine and oxaliplatin are detailed in the instructions for use of marketed products.

BP102 and XELOX are the common first-line standard of care clinically, and their safety profiles are known. The safety of combination with SHR-1701 on this basis is unknown to some extent, but SHR-1701 is a macromolecular drug, and when it is combined with BP102 and XELOX, the possibility of toxicity superposition is low. However, the risk should be closely monitored throughout the clinical study and intervention measures should be taken in a timely manner.

### Known possible benefits

Immediate possible benefits: Bevacizumab combined with XELOX has demonstrated to be effective in patients with metastatic colorectal cancer, and has been recommended by NCCN and CSCO guidelines as the first-line treatment, Patients in both groups have received standard first-line treatment, which could benefit the patients. According to the currently available clinical study data, immunotherapy may further improve the efficacy of first-line treatment, thus SHR-1701 combined with BP102 and XELOX may benefit patients with advanced colorectal cancer. Meanwhile, the subjects in this study can benefit from better care and attention by receiving regular disease monitoring and treatment with study drug free of charge.

Long-term possible benefits: after new therapeutic drugs and new therapeutic methods are validated, more patients can benefit from the same treatment, which is conductive to the survival of patients with tumors.

### Potential benefit-risk assessments

Immunotherapy is a hot study topic in recent years, but the progress in the field of colorectal cancer treatment has been slow in recent years. In this study, the use of Bevacizumab, an anti-angiogenic drug, can promote the infiltration of T cells and normalize tumor blood vessels. On the basis of blocking PD-1/PD-L1 signaling pathway, SHR-1701 can also remove the TGF-β in tumor microenvironment in a targeted manner, thus relieving the inhibition against immune cell activity in tumor microenvironment by immunosuppressive factors, enhancing immune response, and more effectively improving the inhibition of tumor occurrence and development. The combination of chemotherapy can further promote the exposure of tumor antigens and improve the efficacy of immunotherapy, which is an ideal combined strategy for the first-line treatment of colorectal cancer at present. The objective of this clinical study is to explore the efficacy and safety of SHR-1701 combined with BP102 (a biosimilar of Bevacizumab) and XELOX vs. placebo combined with BP102 and XELOX in the first-line treatment of patients with unresectable recurrent or distantly metastatic colorectal cancer, which is expected to bring better survival benefits to majority of patients with colorectal cancer.

Any drug may induce unexpected or even serious adverse reactions. XELOX combined with BP102 is a common regimen clinically, but the safety of the combination of SHR-1701 with BP102 and XELOX is unknown to some extent. In this study, a detailed risk control plan will be established to minimize the medication risks to subjects.

# Study objectives and endpoints

## Objectives

### Phase 2 Study

**Primary objectives:**

- To evaluate the safety of SHR-1701 combined with BP102 and XELOX for the first-line treatment of patients with advanced colorectal cancer;
- To evaluate the objective response rate (ORR, evaluated by the investigator) achieved with SHR-1701 combined with BP102 and XELOX for the first-line treatment of patients with advanced colorectal cancer;

**Secondary objectives:**

- To evaluate the disease control rate (DCR), progression-free survival (PFS), duration of response (DoR) and overall survival (OS) achieved with SHR-1701 combined with BP102 and XELOX for the first-line treatment of patients with advanced colorectal cancer;

**Exploratory objective:**

- To explore the possible influence of immunogenicity of SHR-1701 on efficacy, safety and PK;

### Phase 3 Study

**Primary study objective**

- To evaluate the PFS (evaluated by IRC) achieved with SHR-1701 combined with BP102 and XELOX vs. placebo combined with BP102 and XELOX for the first-line treatment of patients with advanced colorectal cancer;

**Secondary study objectives**

- To evaluate the PFS (evaluated by the investigator), ORR, DoR, DCR and OS achieved with SHR-1701 combined with BP102 and XELOX vs. placebo combined with BP102 and XELOX for the first-line treatment of patients with advanced colorectal cancer;
- To evaluate the safety of SHR-1701 combined with BP102 and XELOX vs. placebo combined with BP102 and XELOX for the first-line treatment of patients with advanced colorectal cancer;

**Exploratory objective:**

- To explore the possible influence of immunogenicity of SHR-1701 on efficacy, safety and PK;

## Endpoints

### Phase 2 Study

**Primary endpoints:**

- Safety:
- ORR (RECIST 1.1 criteria, evaluated by the investigator);

**Secondary endpoints:**

- DCR (RECIST 1.1 criteria, evaluated by the investigator);
- DOR (RECIST 1.1 criteria, evaluated by the investigator);
- PFS (RECIST 1.1 criteria, evaluated by the investigator);
- OS

**Exploratory endpoints:**

- Immunogenicity indicators (ADA, NAb) of SHR-1701, and trough concentration of SHR-1701 (C_trough_);

### Phase 3 Study

**Primary endpoint**

- PFS (RECIST 1.1 criteria, evaluated by IRC);

**Secondary endpoints:**

- PFS (RECIST 1.1 criteria, evaluated by the investigator);
- ORR (RECIST 1.1 criteria);
- DoR (RECIST 1.1 criteria);
- DCR (RECIST 1.1 criteria);
- OS;
- Safety: AE, laboratory test measurements, etc.

**Exploratory endpoints:**

- Immunogenicity indicators (ADA, NAb) of SHR-1701, and trough concentration of SHR-1701 (C_trough_);

# Study design

This study is designed to be a Phase 2/3 study, with Phase 2 being a single-arm, multicenter study, aimed to assess the safety and efficacy of SHR-1701 combined with BP102 and XELOX for the first-line treatment of patients with unresectable recurrent or distantly metastatic advanced colorectal cancer; and Phase 3 being a randomized, double-blind, placebo-controlled, multicenter study, aimed to assess the efficacy and safety of SHR-1701 combined with BP102 and XELOX vs. placebo combined with BP102 and XELOX for the first-line treatment of patients with unresectable recurrent or distantly metastatic colorectal cancer.

**Phase 2 Study**

In Phase 2, it is planned to enroll 61 subjects who will receive SHR-1701 combined with BP102 and XELOX, with the administration route and imaging assessment consistent with those in the Phase 3 test group, with safety and investigator-assessed ORR as the primary study endpoints. Whether to start Phase 3 will be determined according to the safety and efficacy results of Phase 2, and these results will be used as the reference for hypothesis of efficacy in Phase 3.

**Phase 3 Study**

Phase 3 is a randomized, double-blind, placebo-controlled, multicenter study, and it is planned to enroll 378 subjects. The eligible subjects will be randomized to receive SHR-1701 combined with BP102 and XELOX (test group) or placebo combined with BP102 and XELOX (control group) for treatment at a ratio of 1:1, with stratification factors as follows: PD-L1 expression (<1% or ≥1%), location of the primary tumor lesion (left or right) and RAS gene type (mutant type or wild type). PFS assessed by the independent review committee (IRC) based on RECIST v1.1 criteria will be used as the primary study endpoint.

It is planned to conduct an interim analysis when 70% PFS events are collected in Phase 3, and the purpose of this interim analysis is to demonstrate that PFS in the test group is superior to that in the control group.

Subjects will enter the screening period after comprehensive understanding of the study and signing the informed consent form. The screening period of the study is 28 days, and after completion of screening inspection and assessment, subjects who pass the screening will be randomized to the test group or the control group for corresponding treatment.

**Test group:** SHR-1701, 30 mg/kg, intravenous infusion, D1; BP102, 7.5 mg/kg, intravenous infusion, D1: oxaliplatin, 130 mg/m^2^, intravenous infusion, D1; capecitabine, 1000 mg/m^2^/time, oral administration, twice daily for two weeks in each cycle, followed by a week of rest.

**Control group:** Placebo, intravenous infusion, D1; BP102, 7.5 mg/kg, intravenous infusion, D1: oxaliplatin, 130 mg/m^2^, intravenous infusion, D1; capecitabine, 1000 mg/m^2^/time, oral administration, twice daily for two weeks in each period, followed by a period of rest.

For both the test group and the control group, one treatment cycle lasts 21 days, and subjects who have completed up to 8 cycles of combination with oxaliplatin and have no progressive disease or who can benefit from continuing the maintenance treatment in the opinion of the investigator can enter the maintenance treatment stage, and receive treatment with SHR-1701/ placebo combined with BP102 and capecitabine until progressive disease (PD), toxicity intolerance, start of new anti-tumor therapy, subjects’ voluntary withdrawal from the study, or the investigator judges that the subjects need to be withdrawn from the study. The route and dosage of administration in the maintenance phase are the same as before. The maximum medication duration of SHR-1701/ placebo /BP102/ capecitabine is 2 years.

Subjects may experience pseudoprogression after receiving immunotherapy, when PD is determined for the first time according to the RECIST1.1 criteria, but the subjects are clinically stable and can benefit from continuing treatment in the opinion of the investigator, after discussion with the sponsor and obtaining the consent, the medication can be continued, and the subjects should receive imaging again at an interval of at least 4 weeks (±7 days). If the subsequent imaging confirms PD, the subjects should discontinue the treatment, unless the investigator judges that the subjects can continue to benefit from the medication clinically, in this case, the investigator should discuss with the sponsor again, and after obtaining the consent, the subjects should sign the informed consent form of continuing treatment after disease progression, and then they can continue to use the drug until the investigator judges that the subjects cannot benefit from the medication anymore.

The tumor imaging assessment begins at randomization (first administration in Phase 2), and the imaging examination will be performed every 6 weeks (±7 days) for the first 48 weeks and every 12 weeks (±7 days) thereafter to evaluate the efficacy. If clinically indicated, imaging examination and assessment may be conducted additionally at any time. All subjects will complete safety examination and imaging assessment at withdrawal visit. After that, they will enter the safety follow-up visit. The survival follow-up visit will be conducted once every two months starting from the last dose; For those subjects who discontinue the study not due to progressive disease confirmed by imaging assessment, they should continue to receive follow-up visits to monitor tumor progress, and continue to receive imaging assessment at the frequency specified in the protocol until progressive disease, start of new anti-tumor therapy, withdrawal of informed consent, loss to follow-up or death.

The study design diagrams are shown in Figure 2 and Figure 3.


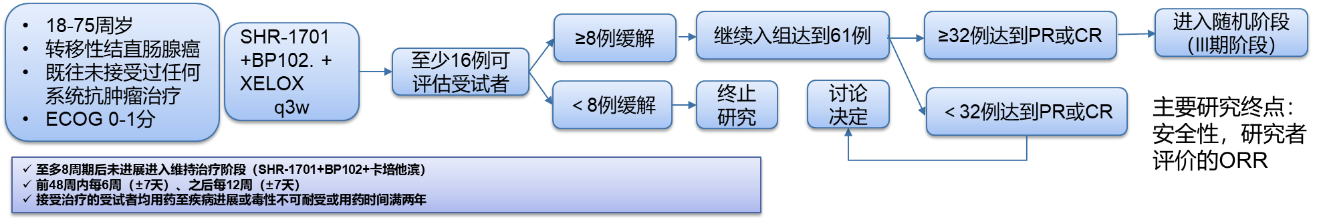


- 18-75 years old
- Metastatic colorectal cancer
- No systematic anti-tumor therapy previously
- ECOG 0-1

At least 16 evaluable subjects

≥8 patients achieve response

< 8 patients achieve response

Continue enrollment until there are 61 subjects

Study discontinuation

Discussion

≥32 subjects achieve PR or CR

< 32 subjects achieve PR or CR

Enter randomization

(Phase 3)

Primary endpoints:

Safety, ORR evaluated by the investigator

- Enter the maintenance treatment stage (SHR-1701+BP102+capecitabine) when there is no disease progression at most after 8 cycles
- Tumor imaging examination: every 6 weeks (±7 days) in the first 48 weeks and every 12 weeks (±7 days) thereafter.
- All subjects receiving treatment receive the drug until disease progression or intolerance of toxicity or expiration of two years of medication

Figure 2 Study design diagram of Phase 2


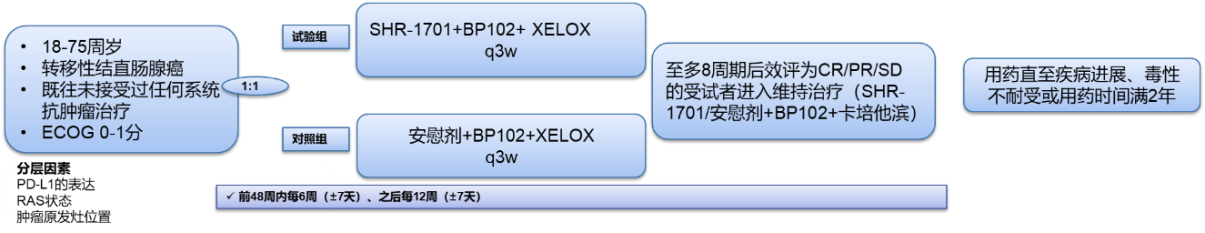


- 18-75 years old
- Metastatic colorectal cancer
- No systematic anti-tumor therapy previously
- ECOG 0-1

**Stratification factors**

Expression of PD-L1

RAS status

Location of tumor primary lesion

- Tumor imaging examination: every 6 weeks (±7 days) in the first 48 weeks and every 12 weeks (±7 days) thereafter.

Test group

Control group

Placebo+BP102+XELOX

q3w

Subjects evaluated as CR/PR/SD after at most 8 cycles enter the maintenance treatment (SHR-1701/placebo +BP102+ capecitabine).

Continue medication until disease progression or intolerance of toxicity or expiration of two years of medication

Figure 3 Study design diagram of Phase 3

# Study population

The inclusion of eligible subjects is an important task to guarantee that the objectives of this clinical study can be achieved. Subjects must meet the following criteria in order to be allowed to participate in the study. All medical or non-medical conditions of the subjects are considered to determine their eligibility.

Before a subject is included in the study, the investigator or his/her team members should review, confirm and record whether the subject is suitable for the study.

## Inclusion criteria

**Patients must meet all of the following criteria to be enrolled to this study.**

1. Patients participate in this study voluntarily and sign the informed consent form;
2. Age ≥ 18 years and ≤75 years, both males and females;
3. Patients with unresectable recurrent or metastatic colorectal adenocarcinoma diagnosed by histology;
4. The subjects are required to provide tissue samples for biomarker detection (such as RAS gene type and PD-L1 expression), the newly acquired tissues are preferred, and patients who cannot provide newly acquired tissues can provide 10-15 archived paraffin sections with a thickness of ≥3μM;
5. Patients who have not received any previous systemic anti-tumor therapy (including but not limited to systemic chemotherapy, molecular targeted therapy, immunotherapy, biological therapy or other study drugs); for subjects who have received previous neo-adjuvant or adjuvant therapy, the first discovery of recurrence or metastasis must be ≥12 months after the last administration of the neo-adjuvant or adjuvant therapy;
6. There is at least 1 measurable lesion according to RECIST v1.1 criteria (lesions which have received previous local treatment such as radiotherapy cannot be regarded as measurable lesions);
7. ECOG score 0-1;
8. Expected survival ≥ 3 months;
9. The functions of important organs meet the following requirements (no blood components, cell growth factors or related drugs are allowed to be used for correction within 2 weeks before the start of study treatment):
10. Absolute neutrophil count (ANC) ≥ 1.5 × 10^9^/L
11. Platelets ≥100×10^9^/L;
12. Hemoglobin ≥9 g/dL;
13. Serum albumin ≥3.0g/dL;
14. Total bilirubin ≤1.5 × ULN, ALT, AST and/or AKP ≤ 2.5× ULN; in case of liver metastasis, ALT and/or AST ≤5 × ULN, and total bilirubin ≤ 3× ULN; in case of liver metastasis or bone metastasis, AKP ≤5 × ULN
15. Creatinine clearance rate ≥50mL/min (calculated according to Cockcroft-Gault equation) or serum creatinine ≤ 1.5× ULN;
16. International standardized ratio (INR) ≤ 1.5×ULN, prothrombin time (PT) and activated partial thromboplastin time (APTT) ≤ 1.5×ULN;
17. Urinary protein < 2+ (if urinary protein ≥2+, 24-hour (h) urinary protein quantification can be performed, and subjects with 24 h urinary protein quantification < 2.0 g can be enrolled);
18. Women with childbearing potential must agree to abstain from sexual intercourse (avoid heterosexual intercourse) or use reliable and effective methods of contraception from the signing of informed consent form to at least 6 months after the last dose of the investigational drug. Furthermore, the serum HCG test must be negative within 3 days before the start of the study treatment, and the subjects must be in non-lactating period. A female patient is considered to have childbearing potential if she has menstruated, has not yet achieved the post-menopausal state (non-menstrual period ≥12 consecutive months, and no other causes have been found except menopause), and has not received sterilization surgery (such as hysterectomy, bilateral tubal ligation or bilateral oophorectomy);
19. Male patients whose partners are women with childbearing potential must agree to abstain from sexual intercourse or use reliable and effective methods of contraception from the signing of informed consent form to at least 6 months after the last dose of the investigational drug. Male patients must also agree not to donate sperm during the same period. Male subjects whose partners are pregnant must use condoms, and other contraceptive methods are unnecessary.

## Exclusion criteria

**Patients are not permitted to enter the study if he/she meets any of the following criteria:**

1. Patients with recurrent or metastatic lesions that can be treated by radical surgery;
2. Patients with central nervous system or meningeal metastasis;
3. The subjects’ tumor type is known to be mis-match repair deficiency/microsatellite instability-high (dMMR/MSI-H);
4. Patients with bleeding tendency, high bleeding risk, coagulation dysfunction or thrombosis tendency:
5. Patients with a history of thrombotic disease within 6 months and/or hemoptysis within 3 months (coughing up at least 1/2 teaspoon of blood (about 2.5 mL) at a time);
6. Patients who have received full-dose oral or injectable anticoagulants or thrombolytic drugs for treatment purposes within 7 days before the start of the study treatment, however, preventive anticoagulation treatment for open intravenous infusion system and preventive use of low molecular weight heparin (enoxaparin ≤40 mg/ day) are allowed;
7. Patients who have used aspirin (> 325 mg/ day), dipyridamole, ticlopidine, clopidogrel, cilostazol or other drugs that inhibit platelet function within 7 days before the start of study treatment;
8. Patients whose CR/MRI suggests that tumor surrounds or invades large blood vessels (such as pulmonary artery or superior vena cava);
9. Patients with moderate or severe ascites with clinical symptoms (that is, those who need therapeutic puncture and drainage within 2 weeks before starting the study treatment, while those who only show a small amount of ascites on imaging without clinical symptoms can be enrolled); uncontrolled or moderate or severer pleural effusion and pericardial effusion;
10. Subjects whose toxicity from previous anti-tumor therapies has not recovered to ≤ CTCAE grade 1 (except for alopecia, achieving the numerical requirements in the inclusion criteria, or other AEs which will not affect the treatment with investigational drug in the opinion of the investigator);
11. Subjects with poorly controlled hypertension (systolic blood pressure ≥140 mmHg and/or diastolic blood pressure ≥90 mmHg under regular antihypertensive treatment), and subjects with previous history of hypertensive crisis or hypertensive encephalopathy;
12. Patients with severe cardiovascular and cerebrovascular diseases, including cerebrovascular accident (CVA), transient ischemic attack (TIA), myocardial infarction and significant vascular diseases (including but not limited to aortic aneurysm requiring surgical repair or recent arterial thrombosis) within 6 months prior to enrollment; patients with poorly controlled clinical symptoms or diseases of the heart, such as unstable angina pectoris, heart failure of NYHA (New York Heart Association ) Grade II or above, left ventricular ejection fraction < 50% by color Doppler echocardiography, or serious arrhythmia that cannot be controlled by medication;
13. Patients who have received major surgery within 4 weeks before the start of the study treatment (major surgery is defined as surgery under general anesthesia that requires at least 3 weeks of recovery time before receiving the study drug treatment); patients with incurable wounds (severe, unhealed or open), active peptic ulcers or untreated fractures; patients with gastrointestinal bleeding (including melena, hematochezia, etc., if the bleeding is confirmed as hemorrhoid bleeding or only manifested as occult blood in stool, the patient can be enrolled), tracheoesophageal fistula, gastrointestinal perforation or gastrointestinal fistula, or abdominal abscess within 6 months before the start of the study treatment; patients with extragastrointestinal bleeding (such as abnormal vaginal bleeding, hematemesis) with CT CAE Grade 3 or above within 6 months or with CTCAE Grade 2 or above within 3 months before the start of the study treatment;
14. Patients with intestinal obstruction or symptoms and signs of intestinal obstruction within 6 months before the start of treatment, but subjects who have received surgical treatment and whose obstruction is completely relieved can be screened; patients who have received previous intestinal stent implantation and whose intestinal stent has not been removed until the screening period;
15. Patients who was found to have active tuberculosis infection by medical history or CT examination, or who have had a medical history of active tuberculosis infection within 1 year prior to enrollment, or who have had active tuberculosis infection before more than 1 year but have not received regular treatment;
16. Patients with active autoimmune disease or history of autoimmune disease (including but not limited to: autoimmune hepatitis, interstitial pneumonia, uveitis, enteritis, hypophysitis, vasculitis, nephritis, hyperthyroidism and hypothyroidism [subjects whose disease can be controlled just by thyroid hormone replacement therapy can be included]); subjects with skin diseases that do not need systematic treatment (such as vitiligo, psoriasis, alopecia), controlled type I diabetes treated with insulin, or asthma that has been completely relieved in childhood and does not need any intervention in adulthood, can be included (patients with asthma who need medical intervention with bronchodilators cannot be included);
17. Subjects with severe infection (CTCAE ≥Grade 3) within 4 weeks before the start of study and treatment, including but not limited to bacteremia, severe pneumonia or other serious infection complications requiring hospitalization; patients with active infection or unexplained fever > 38.5℃ within 2 weeks before the first dose (subjects with fever due to tumor (as judged by the investigator) can be included); patients who have received oral or intravenous administration of therapeutic antibiotics within 2 weeks before the start of the study (patients who use preventive antibiotics can participate in this study);
18. Subjects who are currently accompanied by interstitial pneumonia or interstitial lung disease, or have a history of previous interstitial pneumonia or interstitial lung disease requiring hormone therapy; or subjects with pulmonary fibrosis, organized pneumonia (for example, bronchiolitis obliterans), pneumoconiosis, drug-related pneumonia, or idiopathic pneumonia that may interfere with the judgment and treatment of immune related pulmonary toxicities; or subjects with evidence of active pneumonia or severe impairment of lung function according to the chest computed tomography (CT) images during the screening period;
19. Patients with congenital or acquired immunodeficiency (such as HIV infection); a history of organ transplantation or allogeneic bone marrow transplantation; active hepatitis B (HbcAb and/or HbsAg positive, and HBV-DNA ≥ 2500 copies /mL or 500 IU/ml) or hepatitis C (HCV antibody positive, and HCV-RNA higher than the limit of detection of the analysis method); complicated with hepatitis B and hepatitis C infection (HbsAg or HbcAb positive and HCV antibody positive);
20. Patients who have been diagnosed with other malignant tumors within 5 years before the first use of the study drug, however, patients with effectively treated skin basal cell carcinoma, skin squamous cell carcinoma and/or effectively resected in situ cervical cancer and/or breast cancer may be allowed to be included after assessment;
21. Patients with known allergic reaction, hypersensitivity or contraindication to SHR-1701/ placebo, BP102, oxaliplatin, capecitabine or any component used in these products;
22. Patients who have received treatment with immunosuppressants or corticosteroids (prednisone at a dose of > 10 mg/day or equivalent doses of other hormones) within 14 days before the start of the study treatment for the purpose of immunosuppression; in the absence of active autoimmune diseases, steroid hormones are allowed to be inhaled or used locally, and hormone replacement therapy with prednisone at a dose of ≤ 10mg/ day or equivalent doses of other hormones is allowed;
23. Patients who have participated in other clinical studies within 4 weeks prior to the use of investigational drug in this study (for the subjects who have entered the follow-up visit period, the duration is calculated based on the time of the last use of investigational drug or device) or are participating in other clinical studies.
24. Patients who have previously received treatment with targeted T cell costimulatory molecules or immune checkpoint inhibitors (including but not limited to PD-1/PD-L1 inhibitors, CTLA-4 inhibitors, etc.); patients who have previously received anti-epidermal growth factor receptor therapy (including but not limited to cetuximab, panizumab, etc.) or any anti-angiogenic drug therapy (including but not limited to bevacizumab or its biosimilars, regorafenib, fruquintinib, etc.);
25. Patients who have received live attenuated vaccine treatment within 28 days before the start of the study treatment, or who are expected to need such vaccine during the treatment period or within 60 days after the last dose;
26. Patients who have received radiotherapy for the primary lesions within 6 months before the start of the study treatment; palliative treatment for non-target lesions is allowed to control symptoms, such treatment must be completed 4 weeks before the start of study treatment; patients who have used traditional Chinese medicine or Chinese patent medicine with anti-tumor effect within 2 weeks before the start of the study treatment;
27. Pregnant or lactating women;
28. Other factors of the subjects that may affect the study results or lead to forced discontinuance of the study (as judged by the investigator), such as alcohol abuse, drug abuse, other serious diseases (including mental diseases) requiring combined treatment, seriously abnormal laboratory test values, family or social factors, and other conditions that may affect the safety of the subjects or the collection of study data.

## Randomization criteria

Subjects who meet the eligibility criteria will be randomized to the test group or the control group at a ratio of 1:1 through the randomization system. The randomization will be stratified according to the expression of PD-L1 (< 1% or ≥1%), location of primary tumor lesion (left or right) and RAS gene type (mutant type or wild type).

## Requirements on life style

### Contraception

Male subjects with azoospermia (caused by vasectomy or other underlying diseases) must use condoms and their partners are not required to take contraceptive measures.

Female subjects are considered to be of no childbearing potential in case of the following, and do not need to take contraceptive measures:

1. Postmenopausal state is defined as natural menopause for at least 12 months without other medical causes. The level of follicle stimulating hormone (FSH) reaching the postmenopausal range can be used to confirm the postmenopausal status, if the female subject receives no hormonal contraception or hormone replacement therapy. However, if menopause is less than 12 months, it is insufficient to confirm the postmenopausal status merely based on the FSH results reaching postmenopausal level;

Or

1. Hysterectomy and/or bilateral oophorectomy, bilateral salpingectomy or bilateral tubal ligation/occlusion;

Or

1. Presence of congenital or acquired conditions causing infertility.

Female subjects of childbearing potential and male subjects with female partners of childbearing potential must agree to take contraceptive measures, and must comply with one of the following requirements from signing the informed consent to 6 months after the last dose of the investigational drug:

1. Abstinence (avoiding heterosexual intercourse) †.

Or

1. During heterosexual intercourse, women (subjects or partners) should use highly effective methods of contraception and male subjects must use condoms at the same time.

Highly effective methods of contraception are defined as those which result in less than 1% failure rate of contraception per year if used consistently and correctly, including the following:

- Contraceptives proven to be effective (containing estrogen/progestogen or progestogen), administered orally, by injection, intravaginally, as an implant, or transdermally;
- An intrauterine device (IUD) or an effective intrauterine hormone-releasing system (IUS) is correctly placed;
- Male partner has been vasectomized and sterilized and has been confirmed by medical assessment. For female subject, the male partner should be her only sexual partner.
- Bilateral tubal ligation/bilateral salpingectomy or bilateral tubal occlusion surgery (occlusion surgery has been proven to be effective by relevant instruments).

† The reliability of sexual abstinence needs to be assessed based on the duration of the clinical study and the subject's personal preferences and lifestyle, and abstinence (avoiding heterosexual intercourse) alone is sufficient for contraception in subjects who have always chosen to live a completely asexual life. Periodic abstinence (e.g., based on ovulation, sympto-thermal method), spermicide use only, amenorrhea during lactation, and voitus interruptus are not acceptable methods of contraception.

In addition, the subjects should be aware that once they stop the chosen contraceptive method, or once the subjects or their partners have suspected or confirmed pregnancy, they need to notify the investigator immediately.

## Screening failure

Screening failure is defined as subjects who agree to participate in the clinical trial, but are not randomized to receive medication or fail to participate in the study. It is necessary to record the information of screening failure to ensure the transparency of report, meet the requirements of Consolidated Standards of Reporting Trials (CONSORT) and respond to the query of regulatory authorities. Such information should at least include demographics, detailed information of screening failure, inclusion and exclusion criteria and any serious adverse event.

Subjects who fail the screening may be screened again, at re-screening, the subjects must give informed consent again, and receive a new subject number, and one subject can be re-screened only once.

# Study intervention

For this study, according to the definition of investigational drug in the guidelines (ICH E6 1.33) of International Council for Harmonization of Technical Requirements for Pharmaceuticals for Human Use (ICH) (in clinical study, a drug active ingredient, placebo or drug product of reference drug evaluated, including the new usage of the approved drug, new dosage form or new indications, or more information used to obtain the approved usage), the investigational drugs in this study include SHR-1701/placebo, BP102, oxaliplatin and capecitabine.

## Drug Assignment

The management, dispensing and recovery of clinical medications in this study are in the charge of a designated person. The investigator must ensure that all investigational medications are only used for the subjects participating in this clinical trial, and their dosage and administration should follow the study protocol. The remaining drugs will be returned to the sponsor, and the investigational drugs in this study are not allowed for the treatment not included in this study.

The investigational drug should be stored under the storage conditions specified in the study protocol. When drugs are dispensed to the study site, a drug receipt form should be signed by two persons in two copies, with the clinical study site and sponsor each holding one copy. The remaining drugs and empty boxes be taken back at the end of the study and that both parties sign a drug recovery form. The dispersing and recovery of each drug should be recorded on a special sheet in a timely manner.

The monitor should be responsible for monitoring the supply, use and storage of the clinical investigational medications and the handling process of the remaining drugs.

## Supply of investigational drug

### 5.2.1 Dosage form and packaging

The investigational drugs are provided by the sponsor, uniformly packaged and qualified after inspection (see the corresponding certificate of analysis).

Relevant information of SHR-1701 injection/placebo, BP102 injection, oxaliplatin for injection and capecitabine tablets is as follows:

#### SHR-1701 injection/ placebo

[Manufacturer]: Suzhou Suncadia Biopharmaceuticals Co., Ltd.

[Dosage form]: Injection.

[Strength]: According to the need of clinical study, the strength of this product is temporarily determined to be 6 mL: 0.3 g.

[Batch number]: See the certificate of analysis.

[Route of administration]: intravenous infusion

[Storage condition]: This product should be protected from light, and stored and transported in original package at 2℃-8℃. Do not freeze or shake.

#### BP102 injection

[Manufacturer]: Suzhou Suncadia Biopharmaceuticals Co., Ltd.

[Dosage form]: Injection.

[Strength]: 100 mg, packaged in 4 mL vials.

[Batch number]: See the certificate of analysis.

[Route of administration]: intravenous infusion

[Storage condition]: Protected from light. This product should be stored in a 2-8℃ medical refrigerator, and should not be frozen.

#### Oxaliplatin for injection

[Manufacturer] Jiangsu Hengrui Pharmaceutical Co., Ltd.

[Dosage form]: Lyophilized powder

[Strength]: 50 mg, packaged in vials.

[Batch number]: See the certificate of analysis.

[Route of administration]: intravenous infusion

[Storage condition]: Sealed and stored below 25℃

#### Capecitabine tablets

[Manufacturer] Jiangsu Hengrui Pharmaceutical Co., Ltd.

[Dosage form]: Tablet

[Strength]: 500 mg/tablet; 150 mg/tablet

[Batch number]: See the certificate of analysis.

[Route of administration]: Oral

[Storage condition]: Sealed and stored below 25℃

### Preparation and dispensing

The administration modes of drugs used in this study include intravenous administration and oral administration, and the drugs should be prepared and dispensed by qualified and experienced study staff allowed by the guidelines of the local testing facility, such as doctors, nurses, medical assistants and pharmacists.

The investigational drugs SHR-1701/placebo, BP102 and oxaliplatin should be prepared in a sterile environment. The preparation of SHR-1701/placebo and BP102 is detailed in the drug manual.

Capecitabine is an oral drug, and the investigational drug should be dispensed at each visit according to the study schedule. The qualified staff of study team will dispense the investigational drugs packed in aluminum plastic plate according to the quantity specified in the process each time. During the whole study, subjects should store the drugs in aluminum plastic plate as required in the instructions, and return the aluminum plastic plate at the next visit.

All chemotherapy drugs used in this study are marketed, and the drugs are prepared according to the instructions for use.

## Dose and administration

### Administration

Phase 2 of this study is a single-arm, open-label, multicenter study, with administration route and imaging assessment the same as those in Phase 3 test group. Both oxaliplatin and capecitabine are marketed, and the drug should be administered with reference to the drug instructions for use or clinical routine practice.

Phase 3 is a randomized, double-blind, placebo-controlled, multicenter study. In this process, the drug preparation process is blind, and the dispensing nurse should operate in a separate treatment room to prevent unblinding. The drugs should be consistent in appearance, packaging, labels and other features, and the dispensing nurses should not participate in the administration operation of subjects as far as possible, but other study nurses are responsible for administration.

The administration route of drugs for intravenous injection is shown in Table 2. Capecitabine is an oral drug, at a dosage of 1000 mg/m^2^/time (for the actual dosage each time, refer to the standard dose of capecitabine calculated according to the body surface area in the instructions for use), taken orally twice a day (within 30 min after meal, equivalent to daily total dose 2000 mg/m^2^), the subjects swallow the whole capecitabine tablet without staying or chewing before swallowing. The drug is administered for 2 weeks every cycle, followed by a week of rest, with 21 days as a treatment cycle. The subjects take drugs on the evening of Day 1 (after completion of intravenous drug), until the morning of Day 15, and the specific administration route is shown in Figure 4. If the subjects have discontinued all intravenous drugs, they may start to take capecitabine orally until the evening of Day 14. If the subjects miss capecitabine on the same day, they just need to take the next dose as scheduled, without doubling or supplementation. A subject who takes excessive capecitabine should contact the investigator immediately.

Table 2 administration route of investigational drug for injection

|  | SHR-1701/placebo ^1^ | BP102^2^ | Oxaliplatin ^3^ |
| --- | --- | --- | --- |
| Dose and route of administration | 30mg/kg, IV | 7.5mg/kg, IV | 130mg/m^2^ , IV |
| Infusion speed | 30-60 min, no more than 2 hours | Above 90 min for the first intravenous infusion, and if the first intravenous infusion is well tolerated, the second infusion can last for above 60 min, and if the 60 min of infusion is also well tolerated, the subsequent infusions can be completed within 30 min | At least 2 hours |
| Pre-treatment before administration | No pretreatment required | No pretreatment is required generally, and if necessary, pretreatment can be given according to the clinical practice of the study site | Pretreatment before administration according to the clinical practice of the study site |
| Administration duration | D1 | D1 | D1 |
| Sequence of administration at the stage of combination of four drugs | SHR-1701/placebo, BP102 and oxaliplatin are given in sequence on Day 1 | | |
| Frequency of administration at the stage of combination of four drugs | Every 3 weeks as a treatment cycle (Oxaliplatin: up to 8 cycles) | | |
| Administration frequency at maintenance stage | Every 3 cycles as a treatment period | | |
| Total administration duration | Up to 2 years | | |

1. The dosage of SHR-1701 each time is calculated according to the actual body weight
2. The dose of BP102 is converted according to the weight of the subjects at baseline and remained unchanged throughout the study (unless the weight of the subject changes by ≥10% from the baseline weight).
3. If the body weight of a subject fluctuates by less than 10% from baseline (the day of the first dose) body weight, the baseline body weight is used to calculate the body surface area, and the dosage of chemotherapy drugs is calculated on this basis. Otherwise, the dosage of chemotherapy drugs is calculated according to the actual body weight on the day of planned administration.

For convenience of administration, a deviation of ±5% in the total infusion dosage calculated is allowed in the protocol.

|  |  | **1** | **2** | **3** | **4** | **5** | **6** | **7** |
| --- | --- | --- | --- | --- | --- | --- | --- | --- |
| Week 1 | Morning | Intravenous drug | 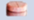 | 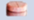 | 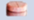 | 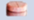 | 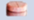 | 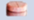 |
|  | Evening | 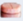 | 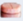 | 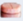 | 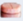 | 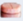 | 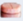 | 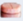 |
| Week 2 | Morning | 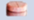 | 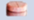 | 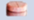 | 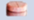 | 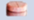 | 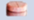 | 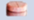 |
|  | Evening | 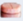 | 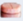 | 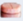 | 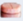 | 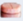 | 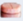 | 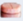 |
| Week 3 | Morning | 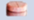 |  |  |  |  |  |  |
|  | Evening |  |  |  |  |  |  |  |

Figure 4 Schematic diagram of administration of capecitabine

The maximum administration duration of oxaliplatin is 8 cycles (no less than 4 weeks in principle, unless the subject has to terminate the treatment due to drug-related toxicity). If the use of SHR-1701/ placebo, BP102, oxaliplatin or capecitabine has been suspended or terminated due to toxicity, other investigational drugs can be continued in the remaining cycles until the criteria for withdrawal from the study are met (up to 8 cycles for combination with oxaliplatin). After completing up to 8 cycles of combined oxaliplatin treatment, maintenance treatment with SHR-1701/ placebo, BP102 and capecitabine will be continued until the criteria for withdrawal from study treatment are met.

XELOX is a commonly used clinical treatment regimen. For the expected adverse reactions of chemotherapy drugs, the investigator can give preventive drugs according to the medication habits at the study site and try to avoid using hormones as preventive drugs.

The administration window period is planned administration date +3 days, and the subsequent administration date is recalculated according to the last actual administration date; exceeding the administration window period will be regarded as delayed administration. If one of the study treatment drugs (SHR-1701/ placebo or BP102 or chemotherapy drugs) is suspended due to its toxic reaction, other drugs should continue to be used as specified before; for example, if the delay is expected to exceed 2 weeks due to the toxicity of chemotherapy/BP102, only SHR-1701/ placebo will be given until the toxicity is restored to the standard of chemotherapy administration, and then the combined administration will be resumed; chemotherapy /BP102 is allowed to be suspended for up to 6 weeks (calculated from the expected time of chemotherapy), and after 6 weeks, chemotherapy will be terminated; if the delay is expected to exceed 2 weeks due to the toxicity of SHR-1701/ placebo, only chemotherapy/BP102 will be given until the toxicity returns to the SHR-1701/ placebo administration standard, and then the combined administration will be resumed. In principle, SHR-1701/placebo is allowed to be suspended for up to 12 weeks, and after 12 weeks, SHR-1701/placebo will be discontinued. If the subjects can continue to benefit from SHR-1701/placebo after suspension for 12 consecutive months in the opinion of the investigator, SHR-1701/placebo can be continued to be used upon communication with the sponsor; in case of the administration should be delayed due to toxicity (relation to which drug is unclear), all drugs need to be delayed simultaneously if it is expected that they can return to the re-administration standard within 2 weeks.

Subjects who have completed up to 8 cycles of combination with oxaliplatin and have no progressive disease or who can benefit from continuing treatment in the opinion of the investigator can enter the maintenance treatment stage, and receive treatment with SHR-1701/placebo combined with BP102 and capecitabine at the same administration route and dosage as before.

SHR-1701 is an immune checkpoint inhibitor, according to the relevant experience with similar drugs, some subjects may have a temporary tumor outbreak in the first few months after starting immunotherapy, and then they will have a disease response, so they are allowed to continue medication after the first occurrence of PD (based on RECIST1.1 criteria).

Tumor outbreaks include any of the following situations:

- Worsening of the original target lesions;
- Worsening of the original non-target lesions;
- New lesions occur.

The investigator may decide whether the subjects should continue the study treatment according to the subjects’ overall clinical condition, including physical performance, clinical symptoms and laboratory test values. If the subjects are clinically stable and can continue to benefit clinically in the opinion of the investigator, upon discussion with the sponsor and after obtaining the consent, the subjects can continue the to receive treatment, and receive tumor assessment again at an interval of at least 4 weeks (±7 days), and iRECIST and RECIST1.1 criteria can be referred to comprehensively. If the subjects are confirmed to have unconfirmed PD, they should continue the study treatment, and if the are confirmed to have confirmed PD, the treatment should be terminated. Unless subjects can continue to benefit clinically in the opinion of the investigator, the subjects should discuss with the sponsor again, and after obtaining the consent, the subjects should sign the informed consent form of continuing treatment again after disease progression, and then they can continue to use the drug until the subjects cannot continue to benefit clinically in the opinion of the investigator. For clinically unstable subjects, the treatment should be terminated after the first evaluation of PD, and there is no need to repeat imaging examination to confirm PD.

Definition of clinical stability:

- There is no significant decrease in subjects’ performance status, and no significant worsening of tumor related symptoms;
- There is no rapid disease progression;
- There are no progressive tumors (such as spinal cord compression) at important anatomical sites that require other emergency medical interventions.

For confirmed PD, refer to the criteria listed in Table 3.

Table 3 Criteria for confirmed PD

|  | **Situations in which PD can be confirmed (any of the situations below)** | **Situations in which PD cannot be confirmed (meeting all situations below)** |
| --- | --- | --- |
| Target lesion | Increase of absolute value of tumor load by ≥ 5 mm compared with the first progression. | Increase of absolute value of tumor load by < 5mm compared with the first progression. |
| Non-target lesion | Compared with the first progression, the non-target lesion continues to progress definitely (qualitative). | Compared with the first progression, there is no definite progression (qualitative). |
| New lesions | 1. New lesions occur compared with the first progression; 2. If a new lesion has occurred before, the new lesion is enlarged, or other new lesions occur. | 1. There are no other new lesions compared with the first progression; 2. If a new lesion has occurred before, the new lesions are stable or shrink. |

For subjects with PD evaluated for the first time, regardless of whether they continue the study treatment after the progression, the initial progress date evaluated by the investigator will be used for all statistical analysis containing progression information.

### Dose interruption

#### Dose adjustment

##### Overall principle

During the study, the investigator is allowed to modify the drug dosage according to the subject's status and clinical experience.

- Every effort should be made to enable each subject to use the investigational drug according to the requirements of the protocol;
- The reasons for dose adjustment or delayed modification, measures taken and results should be recorded in the patients’ medical record and eCRF;
- Both oxaliplatin and capecitabine are marketed, and BP102 is a biosimilar of Bevacizumab (Avastin), both of which are commonly used clinically. Doses can be modified based on the instructions for use and investigator’s clinical experience; in the process of combined treatment, the dose of each drug will be adjusted according to its own situation; if some drugs cannot be continued to be administered because of the related toxicity, other investigational drugs can be used for further treatment in the benefit of subjects in the opinion of the investigator.
- For the delay in treatment due to adverse events, SHR-1701/placebo cannot be delayed for more than 12 weeks, and BP102 and chemotherapy cannot be delayed for more than 6 weeks (calculated from the date of planned medication), and if subjects still can benefit from the treatment after the time of discontinuance above in the opinion of the investigator, the investigator can communicate with the sponsor for further medication.
- In case of several toxic reactions with different grades or severities, the dose will be modified according to the maximum grade observed;
- If one of the investigational drugs (SHR-1701/ placebo or BP102 or chemotherapy drugs) is suspended due to its toxic reaction, other drugs should continue to be used as specified before.

##### Dose adjustment of SHR-1701

In principle, it is not allowed to increase or decrease the dosage of SHR-1701. If the treatment with SHR-1701 is suspended due to toxicity, the subjects can continue to receive treatment at the original dose when the toxicity is improved.

Delayed medication is recommended if a subject experiences the following during treatment:

- Grade 3 drug related skin reaction;

- Grade 3 drug related laboratory abnormalities (excluding lymphocytosis, asymptomatic abnormality of amylase or lipase)

- Grade 3 lymphopenia does not require delayed administration;

- Grade 3 amylase or lipase is abnormal, but there is no need to delay the administration when there are no symptoms or manifestations related to pancreatitis.

When the drug related toxicity recovers to ≤ Grade 1 or baseline level, the study treatment can be resumed. Subjects with drug discontinued should receive relevant safety assessment/inspection once a week or more frequently until the study treatment is resumed. If the toxicity cannot return to the baseline level or Grade 0-1 12 weeks after discontinuance, permanent termination of treatment should be considered.

After 12 weeks of interruption of administration, a subject who is recovering from toxic effects may consider resuming administration, provided that the subject is considered to benefit from resuming administration in the professional opinion of the investigator, but the decision to resume administration should be made through discussion between the investigator and the sponsor.

For the same adverse event, up to two dose interruptions are allowed in the study, unless special circumstances are discussed with the sponsor in advance.

For subjects who can benefit from the study treatment, if they meet the above criteria for terminating SHR-1701 administration, the investigator and the sponsor can discuss and decide whether the subjects can continue the study treatment to ensure their maximum benefit.

##### Dose adjustment of BP102

BP102 is a biosimilar of Bevacizumab, and the dose of BP102 will not be modified during the study unless the weight of the subject changes by ≥ 10% from the baseline. If the treatment is suspended due to BP102-related toxicity, the subject can continue to receive treatment at the original dose after the toxic reaction returns to the baseline level or at least the symptoms are alleviated to CTCAE Grade 1 or below.

In case of Grade 3 or Grade 4 BP102 related toxicity reactions, whether the subjects should continue or terminate the use of BP102 with reference to the following criteria.

1. **First occurrence:**

- Suspend BP102 until the toxic reaction symptoms return to the baseline level or at least the symptoms are alleviated to CTCAE Grade 1 or below (except for the special circumstances listed later);

It should be noted that in case of febrile neutropenia Grade 4 and/or thrombocytopenia Grade 4, BP102 should be suspended until it recovers or at least reaches CTCAE Grade 1 or below.

1. **Re-occurrence after resumption of medication:**

- If the Grade 3 toxic reaction related to the use of BP102 occurs again, the investigator should evaluate the risk/benefit of the patient's continued use of the investigational drug. If this toxic reaction occurs again after the reuse of BP102, the use of BP 102 should be permanently terminated;
- If the Grade 4 toxic reaction related to the use of BP102 occurs again, the use of BP102 should be permanently terminated.

1. Measures to be taken for the investigational drug under the following special circumstances (CTCAE version 5.0 is used for the following grading):

**Bleeding**

Patients with Grade 3 or Grade 4 bleeding event should receive the corresponding treatment, and BP102 treatment should be terminated permanently;

**Thrombosis/embolism**

1. Patients with any grade of arterial thrombosis should permanently terminate treatment with BP102;
2. Patients with Grade 4 venous thrombosis should permanently terminate treatment with BP102;
3. Patients with Grade 3 venous thrombosis should suspend treatment with BP102; If the planned treatment dose of anticoagulant therapy is less than 2 weeks, the treatment with investigational drugs should be suspended until the end of anticoagulant therapy. If the planned treatment dose of anticoagulant therapy is > 2 weeks, the treatment with BP102 should be suspended for 2 weeks, and the treatment with investigational drug can be resumed during the anticoagulant therapy until the following criteria are met:
   1. Before treatment with investigaitonal drugs again, the INR should be within the target (generally 2-3);
   2. Patients should have no Grade 3 or 4 bleeding events after entering this study;
   3. No evidence of tumor invasion or adjacency to large vessels has been found in the previous tumor evaluation.

Note: The treatment dose of anticoagulation therapy is defined as the gradual increase of dose of warfarin or other anticoagulants, to a dose level that maintains INR of no less than 1.5 (generally between 2 and 3). The dose of warfarin should be recorded in eCRF, and INR should be monitored throughout the treatment period for patients receiving anticoagulant therapy.

**Hypertension**

Patients should regularly measure their blood pressure in order to monitor the occurrence and worsening of hypertension. During measurement of blood pressure, patients should be at rest for more than 5 minutes before measuring the blood pressure.

- Grade 1: No intervention is required.
- Grade 2: BP102 is suspended, and once the blood pressure is controlled to be <140/90 mmHg, patients can continue to receive the investigational drug.
- Grade 3: For persistent or symptomatic hypertension, treatment with investigational drugs should be suspended; if the hypertension cannot be controlled, treatment with investigational drugs should be permanently terminated.
- Grade 4: Life-threatening (malignant hypertension or persistent nerve injury, hypertensive crisis, including hypertensive encephalopathy). In case of Grade 4 hypertension, treatment with investigaitonal drugs should be discontinued permanently.

The dosage of antihypertensive drugs used should be recorded at each visit.

Patients should record the dosage of antihypertensive drugs used at each visit. If hypertension still exists when the treatment is terminated, the blood pressure and the use of antihypertensive drugs should be monitored every month until the blood pressure returns to normal, is reasonably explained or the study is ended.

**Reversible posterior leukoencephalopathy syndrome (RPLS)**

There are rare reports that patients treated with the investigational drugs have symptoms and signs consistent with RPLS. This is a rare neurological disease, and its symptoms and signs are: epilepsy, headache, mental state change, visual impairment, or cortical blindness, with or without hypertension. Patients with RPLS should permanently terminate treatment with BP102;

**Proteinuria**

First occurrence of proteinuria:

- <2+: Continue administration of investigational drug as scheduled, without additional examination.
- ≥2+: Continue administration of investigational drug as scheduled, and test the 24 h proteinuria within 3 days before the next treatment cycle:

1. 24 h proteinuria ≤ 2g: Continue administration of investigational drug as scheduled. Receive urinalysis before each planned administration of investigational drugs.
2. 24 h proteinuria >2 g: suspend the current planned administration of investigational drugs, and detect the 24 h urinary protein within 3 days before the next planned administration. Postpone the administration of investigational drugs, until the 24 h urine protein is ≤2 g. Detect the 24 h urinary protein before each administration of investigational drug subsequently, until it is decreased to be ≤1g /24 h. Suspend the administration of investigational drug only when it is >2 g.

Re-occurrence of proteinuria after the second and subsequent administrations

- <3+: Continue administration of investigational drug as scheduled, without additional examination.
- ≥3+: Continue administration of investigational drug as scheduled, and test the 24 h proteinuria within 3 days before the next treatment cycle:

1. 24 h proteinuria ≤ 2g: Continue administration of investigational drug as scheduled.
2. 24 h proteinuria >2 g: suspend the current planned administration of investigational drugs, and detect the 24 h urinary protein within 3 days before the next planned administration. Postpone the administration of investigational drugs, until the 24 h urine protein is ≤2 g. Detect the 24 h urinary protein before each administration of investigational drug subsequently, until it is decreased to be ≤1g /24 h. Suspend the administration of investigational drug only when it is >2 g.

- Nephrotic syndrome (Grade 4): Discontinue the treatment with investigational drug permanently.

**Heart failure**

In case of ≥ Grade 3 left ventricular systolic dysfunction, BP102 treatment should be permanently terminated.

**Gastrointestinal perforation**

In case of gastrointestinal perforation, corresponding treatment measures should be taken and treatment with BP102 should be permanently terminated.

**Wound healing complication**

Drug treatment cannot be started within 28 days after major surgery, or before the surgical wound is completely healed. If a patient has wound healing complications during the study drug treatment, treatment with BP102 should be suspended until the wound is completely healed. If any elective surgery is needed, BP102 treatment should be suspended first.

**Abdominal abscess or fistula**

A patient who has abdominal abscess or sinus should terminate the treatment with BP102. However, if the patient's above problems are solved, the investigator may judge whether to continue to use BP102.

**Infusion reactions and allergic reactions**

- In case of mild infusion reaction during the 60-min infusion, the infusion time of the next administration will remain unchanged; if there are infusion-related adverse events during the 60-min infusion, the subsequent administration should be completed in more than 90 min.
- Similarly, if there are mild infusion-related adverse events during the 30-min infusion, the subsequent administration should be completed in more than 60 min. Patients with grade 3 infusion-related reactions should suspend BP102, and the drug administration should not be restarted on the same day.
- Serious allergic reaction is defined as shock (systolic blood pressure less than 90mmHg and fluid replacement ineffective) within 30 min of BP102 infusion caused by allergy, with or without respiratory distress. The skin symptoms include itching, urticaria and angioedema. Patients with BP102 allergic reaction will terminate treatment with BP102.

##### Modification of XELOX dose

The initial dose of oxaliplatin is 130 mg/m², and that of capecitabine is 1000mg/m²/time. Because XELOX is a standard treatment regimen in guidelines, and it is a commonly used chemotherapy regimen clinically, the dose adjustment and treatment/re-treatment standard of oxaliplatin and capecitabine can be determined by the investigator according to clinical routine practice. The dose can be reduced by 25% and 50% according to the toxicity, and after decrease, the dose cannot be increased again. The dose adjustment of oxaliplatin and capecitabine can be found in Table 4 and Table 10.

Table 4 Dose adjustment for non-hematological toxicities of oxaliplatin

| Adverse event/CTCAE grade | Grade 3 | Grade 4 |
| --- | --- | --- |
| Diarrhea | Dose reduction by 25% | |
| Nausea and/or vomiting | Effective preventive antiemetic treatment has been conducted, and the dose has been decreased by 25%. | Dose reduction by 25% |
| Skin toxicity | It is unnecessary reduce the dose to extend the rest period of chemotherapy until it recovers to ≤Grade 1 | |
| Stomatitis | It is unnecessary to reduce the dose | Dose reduction by 25% |

Table 5 Dose adjustment for neurological toxicities of oxaliplatin

| Neurological toxicity grade | Neurological toxicity reaction | Duration of toxic reaction | | |
| --- | --- | --- | --- | --- |
|  |  | 1-7 day | >7 days | Ongoing during the chemotherapy |
| 1 | Paresthesia/sensory disorder, but it does not affect the function. | Maintain original dose | Maintain original dose | Maintain original dose |
| 2 | Paresthesia/sensory disorder, it affects the function, but does not affect daily activities | Maintain original dose | Maintain original dose | Dose reduction by 25% |
| 3 | Persistent paresthesia/sensory disturbance, with pain and functional injury, and affecting daily activities. | Maintain original dose | Dose reduction by 25% | Discontinue treatment permanently |
| 4 | Persistent paresthesia/sensory disturbance, leading to loss of functions or threatening life. | Discontinue treatment permanently | Discontinue treatment permanently | Discontinue treatment permanently |

Table 6 Dose adjustments for nonhematologic toxicities of capecitabine

| Adverse event/CTCAE grade | Grade 2 | Grade 3 | Grade 4 |
| --- | --- | --- | --- |
| First occurrence | Suspend treatment  Until Grade 0-1  Original dose | Suspend treatment  Until Grade 0-1  Dose reduction by 25% | Stop treatment or treat until Grade 0-1 for the best benefit of patients  50% of the original dose |
| Second occurrence | Suspend treatment  Until Grade 0-1  Dose reduction by 25% | Suspend treatment  Until Grade 0-1  50% of the original dose | Discontinue administration |
| Third occurrence | Suspend treatment  Until Grade 0-1  50% of the original dose | Discontinue administration |  |
| Fourth occurrence | Discontinue administration |  |  |

Table 7 Dose adjustments for hematologic toxicities of XELOX - neutropenia (ANC)

| Adverse event/CTCAE grade | Grade 2  1.0≤ANC≤1.5×10^9^/L | Grade 3  0.5≤ANC≤1.0×10^9^/L | Grade 4  ANC＜0.5×10^9^/L |
| --- | --- | --- | --- |
| First occurrence | No modification is required | Reduce the dose of capecitabine by 25%  Reduce the dose of oxaliplatin by 25% | Reduce the dose of capecitabine by 25%  Reduce the dose of oxaliplatin by 50% |
| Second occurrence | No modification is required | Reduce the dose of capecitabine by 25%  Reduce the dose of oxaliplatin by 50% | Discontinue administration |
| Third occurrence | No modification is required | Discontinue administration  Unless the subjects can benefit from further treatment, capecitabine monotherapy can be continued, with dose reduced by 25%. | NA |

Table 8 Dose adjustments for hematologic toxicities of XELOX - neutropenic fever (ANC)

| Adverse event/CTCAE grade | Grade 3  ANC<1.0×10^9^/L, with fever ≥38.5℃ | Grade 4  ANC<1.0×10^9^/L, with fever ≥38.5℃ and life-threatening septicopyemia |
| --- | --- | --- |
| First occurrence | Reduce the dose of capecitabine by 25%  Reduce the dose of oxaliplatin by 50% | Discontinue administration  Unless the subjects can benefit from further treatment, capecitabine monotherapy can be continued, with dose reduced by 50%. |
| Second occurrence | Discontinue administration  Unless the subjects can benefit from further treatment, capecitabine monotherapy can be continued, with dose reduced by 50%. | Discontinue administration |

Table 9 Dose adjustments for hematologic toxicities of XELOX -thrombocytopenia

| Adverse event/CTCAE grade | Grade 2  50-75×10^9^/L | Grade 3  25-50-×10^9^/L | Grade 4  ＜25×10^9^/L |
| --- | --- | --- | --- |
| First occurrence | No dose modification is required | Reduce the dose of capecitabine by 25%  Reduce the dose of oxaliplatin by 25% | Reduce the dose of capecitabine by 50%  Reduce the dose of oxaliplatin by 50% |
| Second occurrence | No dose modification is required | Reduce the dose of capecitabine by 25%  Reduce the dose of oxaliplatin by 50% | Discontinue administration  Unless the subjects can benefit from further treatment, capecitabine monotherapy can be continued, with dose reduced by 50%. |
| Third occurrence | No dose modification is required | Reduce the dose of capecitabine by 50%  Reduce the dose of oxaliplatin by 50% | Discontinue administration |

Table 10 Criteria for administration of treatment/re-treatment with XELOX

| Laboratory tests and clinical symptoms | Starting standard |
| --- | --- |
| Absolute neutrophil | ≥1500/mm^3^(1.5×10^9^/l) |
| Platelet count | ≥75×10^9^/l |
| Diarrhea | ≤Grade 1 |
| Non-hematological toxic reaction | ≤Grade 1 |

### Treatment time

**Phase 2 Study**

All subjects will receive treatment with SHR-1701 combined with BP102

Subjects who have completed up to 8 cycles of combination with oxaliplatin (no less than 4 cycles in principle, unless the subjects have to terminate the treatment due to drug-related toxicity) without progressive disease or who can benefit from continuing treatment in the opinion of the investigator can enter the maintenance treatment stage, and receive treatment with SHR-1701 combined with BP102 and capecitabine until progressive disease (PD), toxicity intolerance, start of new anti-tumor treatment, subjects’ voluntary withdrawal from the study, and the subjects should withdraw from the study in the opinion of the investigator. The route and dosage of administration in the maintenance phase are the same as before. The maximum medication duration of SHR-1701/ placebo /BP102/ capecitabine is 2 years.

**Phase 3 Study**

Subjects in the test group and control group will receive treatment with SHR-1701 or placebo combined with BP102 and XELOX Subjects who have completed up to 8 cycles of combination with oxaliplatin (no less than 4 cycles in principle, unless the subjects have to terminate the treatment due to drug-related toxicity) without progressive disease or who can benefit from continuing treatment in the opinion of the investigator can enter the maintenance treatment stage, and receive treatment with SHR-1701/placebo combined with BP102 and capecitabine until progressive disease (PD), toxicity intolerance, start of new anti-tumor treatment, subjects’ voluntary withdrawal from the study, and the subjects should withdraw from the study in the opinion of the investigator. The route and dosage of administration in the maintenance phase are the same as before. The maximum medication duration of SHR-1701/ placebo /BP102/ capecitabine is 2 years.

### Subject compliance

Capecitabine is an oral drug, and the subjects will take the drug at home and will be required to return all capecitabine not taken in the previous cycle before the start of the next cycle. The number of tablets returned by the subjects will be counted, recorded and archived. Subjects should fill in the subject log as required, and clearly record the time of medication, meal time, dosage and other information.

Other drugs are for intravenous infusion, and the drug preparation will be completed and recorded by the site in accordance with the investigational drug manual. The documentation system of the study site should record all relevant information about dose preparation and administration.

## Storage of investigational drug

The investigator, or his/her authorized representative (e.g., pharmacist) will ensure that all the investigational drugs are stored in a secure, controlled access area under storage conditions and are stored in accordance with applicable regulatory requirements.

The investigational drugs should be stored according to the storage conditions listed in 5.2.1, and in case of inconsistency between the protocol and other data, SHR‑1701/placebo and BP102 should be stored according to the storage conditions on the labels, and capecitabine and oxaliplatin should be stored according to the storage conditions in the marketing instructions.

The investigational drug should be stored in its original container and consistent with the drug label. Once the product is prepared or diluted, refer to the manual or instructions for use of investigational drug for storage conditions after preparation.

The site must be able to record the maximum and minimum temperature of all investigational drug storage locations (e.g., frozen, refrigerated, or at room temperature) for each working day. The recording period should begin with the receipt of the drug until all remaining investigational drugs have been recovered. Temperature monitoring devices and storage devices (e.g., refrigerators) should be checked periodically by the site to ensure proper functioning.

Any deviation from the product labeling conditions should be promptly reported upon discovery. The site should take active measures to place the product under the labeled storage conditions as soon as possible, and at the same time, report temperature excursions and actions taken to the sponsor.

Investigational drugs affected by temperature excursion should be temporarily quarantined in an environment consistent with drug storage and should not be used until permission is received from the sponsor. If the sponsor allows the further use of investigational drug after temperature deviation, the deviation should not be recorded as protocol deviation. However, further use of the investigational drug before the sponsor permits is considered as protocol deviation. The sponsor will provide the site with specific steps to report temperature excursions.

Because subjects are allowed to take capecitabine home for use, staff at the study site will instruct the subjects the method of correct storage of drugs.

## Record of investigational drugs

The site must maintain the records of the supply of investigational drugs, including receipt, use, loss or other destinations. All investigational drugs will be recorded in drug record form.

For capecitabine, the subjects will be required to return all the unused investigational drugs and packaging in the previous cycle before the start of the next cycle, and the subjects' log cards will be recovered. The number of capecitabine tablets returned by the subjects will be counted, recorded and archived.

### Destruction of investigational drug

The sponsor or its authorized personnel will provide the study site guidance on how to destroy the investigational drugs. If the site is authorized to destroy the investigational drug, the investigator must ensure that the destruction of the investigational drug complies with applicable environmental regulations, institutional policies and other applicable provisions, and relevant processes for destruction are provided. All destructions should be recorded.

## Prior and concomitant medication

### Prior medication

In this study, all previous medications within 30 days before signing of informed consent form should be collected.

In this study, subjects who have not received any previous systemic anti-tumor treatment for metastatic colorectal cancer, including but not limited to systemic chemotherapy, molecular target drug therapy, immunotherapy, biological therapy and other study treatment drugs, will be included. For the subjects who have received previous postoperative adjuvant chemotherapy, the first discovery of recurrence or metastasis must be ≥12 months after the last dose of adjuvant chemotherapy. Meanwhile, patients who have not previously received treatment with targeted T cell costimulatory molecules and immune checkpoint inhibitors (including but not limited to PD-1/PD-L1 inhibitors, CTLA-4 inhibitors, etc.); patients who have not received previous anti-epidermal growth factor receptor therapy (including but not limited to cetuximab, panizumab, etc.) or any anti-angiogenic drug therapy (including but not limited to bevacizumab or its biosimilars, regorafenib, fruquintinib, etc.);

Subjects must not receive oral or intravenous therapeutic antibiotics (patients receiving preventive antibiotics are eligible to participate in the study) or immunosuppressant or systemic hormone therapy for immunosuppression (> 10mg/ day prednisone or other therapeutic hormones) within 2 weeks before starting the study treatment.

The subjects must have not received live attenuated vaccine treatment within 28 days before the start of the study treatment.

Radiotherapy for primary lesions should be completed within 6 months; palliative radiotherapy for non-target lesions allowed to control symptoms must be completed at least 4 weeks before the start of the study treatment, and the adverse events caused by radiotherapy should be recovered to ≤ CTCAE Grade 1.

### Concomitant medications or concomitant therapies

Concomitant medications are the therapies administered at the investigator's discretion and in consideration of the subject's interest.

All concomitant medications, blood products and non-drug interference (such as puncture) received by subjects from the screening period to the end of safety follow-up period or before start new anti-tumor treatment (whichever occurs first and then only the concomitant medications for the treatment of adverse events related to the study drug will be recorded) will be recorded in CRF.

Drugs or vaccines specifically prohibited in the EXCLUSION CRITERIA are not allowed throughout the study. If a subject develops a comorbidity that necessitates the use of prohibited medications, and may require discontinuance of study drug treatment and receipt of prohibited medications, the investigator will need to discuss with the sponsor, and the decision for the subject to continue study treatment or receive prohibited medications is ultimately at the discretion of the investigator, the sponsor, and the subject.

Other drugs that should be prohibited for oxaliplatin and capecitabine are detailed in the marketing instructions.

#### Other anti-tumor/anti-cancer therapies or investigational drugs

When receiving the study treatment, subjects will not be allowed to receive other anti-tumor treatments not specified in this study protocol, including modern Chinese medicine preparations marketed for anti-tumor treatment (see Attachment 4-Prohibited traditional Chinese medicine during study for details) and immunomodulators (including but not limited to interferon, interleukin -2, thymosin, lentinan, etc.).

Subjects are not allowed to participate in other drug/device clinical trials.

Subjects are not allowed to receive other anti-tumor treatments, such as chemotherapy, molecular targeted therapy, hormone therapy, immunotherapy, biotherapy and radiotherapy (except for local treatment as specified below).

Patients can receive bisphosphonates to treat bone metastases. If the painful bone metastasis cannot be effectively controlled by systemic therapy or local analgesia, palliative radiotherapy is allowed for a small area (the radiotherapy area must be < 5% of the bone marrow area, and it cannot be targeted at the target lesion. For the bone marrow content of human body, refer to Attachment 3-Percentage of human bone marrow content).

Palliative treatment is allowed for local lesions that cause significant symptoms, such as bone pain lesions, and local radiotherapy or surgery can be considered, but the following conditions must be met. Also, it is recommended to discuss with the sponsor before starting palliative local treatment.

1. For the subjects who need local treatment due to the aggravation of symptoms during the study, the investigator must determine whether there is a disease progression;
2. Subjects with disease progression must meet the criteria for continuing treatment after disease progression;
3. The locally treated lesion cannot be a target lesion.

#### Supportive treatment

Palliative and supportive treatment for disease-related symptoms will depend on the investigator's judgment and relevant guidelines, such as Guidelines of American Society of Clinical Oncology.

#### Vaccines

It is not allowed to receive live vaccines within 28 days before the first use of the investigational drug and during the participation in the study, including but not limited to measles, epidemic mumps, rubella, chickenpox, yellow fever, rabies, BCG and typhoid vaccines. Inactivated virus vaccines for seasonal influenza by injection are allowed, but attenuated live influenza vaccine for intranasal administration is not allowed.

#### Hematopoietic growth factors and blood transfusion

No blood components, cell growth factors and hematopoietic growth factors are allowed to be used 2 weeks before the start of study treatment.

Hematopoietic growth factors including G-CSF can be used depending on the actual clinical judgment during treatment.

#### Treatment of diarrhea and vomiting

The selection of preventive drugs should be judged and decided by the investigator, provided that the preventive drugs are not listed as prohibited drugs in the previous chapter, and there is known or foreseeable drug interaction during treatment.

#### Anti-inflammatory/analgesic therapy

If there is no known or foreseeable drug interaction, and it is not prohibited in the protocol, anti-inflammatory or narcotic analgesic can be given.

#### Immune inhibitors and corticosteroids

Immunosuppressant therapy is not allowed (if it is necessary to use it because of drug-related adverse events, it must be discussed with decided by the sponsor).

Long-term, systemic use of corticosteroids is not permitted. Cumulative use of corticosteroids according to the instructions for no more than 1 week as prophylactic medication for contrast media allergy is allowed. Systemic treatment with corticosteroids to individual subjects after discussion with the sponsor is permitted. Short-term (no more than 3 weeks) and low dosage (≤10mg prednisone equivalent dosage) use of corticosteroids for the treatment of non-autoimmune diseases (e.g., delayed hypersensitivity reactions due to contact allergens) is permitted.

However, emergency use, topical application, inhalation by spray, eye drops or local injection of corticosteroids are permitted. Systemic use of corticosteroids (≤ 10 mg/day prednisone or equivalent) at physiologic replacement doses (e.g., adrenal replacement steroid dose) is permitted.

#### Surgery

Any surgery performed during the study should have its theoretical basis and necessity, and any surgery should be decided upon joint discussion with the sponsor. The interval between surgery and administration of BP102 must not affect the recovery of wound and the locating of unexplained bleeding as far as possible. It is suggested that BP102 and SHR-1701/ placebo should be discontinued 6 weeks before surgery, and chemotherapy drugs can continue to be used until 3 weeks before surgery.

### Supportive care

Palliative and supportive care for disease-related symptoms will depend on the investigator's judgment and relevant guidelines, such as Guidelines of American Society of Clinical Oncology.

During treatment, subjects should be given the best supportive treatment.

Subjects with uncontrolled tumor-related pain are not recommended to be enrolled. Subjects who require analgesic treatment must have a stable analgesic treatment regimen before enrollment; symptomatic lesions suitable for palliative radiotherapy (such as bone metastasis or metastasis invading nerves) should be treated at least 4 weeks before enrollment; for asymptomatic metastatic lesions, if their further growth may lead to dysfunction or intractable pain (if there is no epidural metastasis showing spinal cord compression), if appropriate, local/regional treatment should be considered before starting study treatment.

The original hormone replacement therapy is permitted. For example, Subjects with a history of autoimmune-mediated hypothyroidism who are receiving a stable dose of thyroid replacement hormone can be enrolled in this study. Subjects with type 1 diabetes who are receiving a stable insulin regimen and whose blood glucose is controlled can participate in this study.

#### Suggestions for symptomatic treatment of common adverse reactions with immunotherapy antibody drugs

##### Safety management rules for immune oncology drugs

Adverse events caused by immune-oncology (I-O) drugs are different from other types of anti-tumor drugs, and their severity and duration are special. SHR-1701 is such a kind of drug, so it is necessary to early identify and deal with the adverse events caused by it to reduce the occurrence of serious toxic events. Reference can be made to relevant guidelines such as Toxicity Management of Immunotherapy: Guidelines for Clinical Time for Diagnosis, Treatment and Follow-up Visit of ESMO and Guidelines for Toxicity Management of CSCO Immune Checkpoint Inhibitors to assist the investigator in assessing and dealing with the following systemic adverse events: gastrointestinal tract, kidney, lung, liver, endocrine, skin and nerve. The following are suggestions for the treatment of immune-related toxicity for reference.

- **Immune-related skin toxicity**
- For patients with Grade 1-2 skin adverse events, the study and treatment can be continued (at least 1 week). In case of skin itching, skin cream, oral antihistamine and/or low-dose glucocorticoid ointment should be used for external treatment. The study treatment will be continued again when the skin adverse events are reduced to below Grade 1.
- For patients with grade 3 skin adverse events, the study treatment should be interrupted, and external use of moisturizer, oral antihistamine and high-dose glucocorticoid ointment should be started immediately.
- For patients with Grade 4 skin adverse events, the study treatment should be discontinued permanently, and the patients should be hospitalized immediately and consulted by dermatologists. Glucocorticoid (methylprednisolone 1-2 mg/kg) should be started as soon as possible, and gradually reduced according to the changes of adverse events.
- **Immune-related pneumonia**
- In the clinical study of SHR-1701, the signs and symptoms of patients with immune-related pneumonia, such as cough and chest discomfort, will be monitored.
- For patients with Grade 2 pneumonia, the study treatment should be interrupted, and oral treatment with methylprednisolone 1-2 mg/kg should be started after infectious pneumonia is ruled out.
- For patients with Grade 3-4 pneumonia, the study treatment should be discontinued permanently. Also they should receive high-dose methylprednisolone 2-4 mg/kg intravenous injection immediately. If the condition worsens during hormone therapy, infliximab, mycophenolate mofetil or cyclophosphamide can be added and reduced after 4-6 weeks.
- **Immune related gastrointestinal toxicity**
- In the clinical study of SHR-1701, the signs and symptoms of patients with immune-related enteritis, such as abdominal pain, diarrhea and hematochezia, will be monitored.
- For patients without severe diarrhea (Grade 1), the study treatment can be continued, and antidiarrheal drugs should be given (such as ropivacrol).
- Patients with Grade 2 diarrhea should interrupt the study treatment and start glucocorticoid therapy (budesonide or oral glucocorticoid 1 mg/kg) according to the severity of diarrhea and other symptoms. If there is no improvement after 3-5 days of treatment, colonoscopy should be performed.
- Patients with serious diarrhea (Grade 3-4) should discontinue the study treatment permanently and receive intravenous treatment with methylprednisolone 2 mg/kg.
- **Immune-related liver toxicity**
- In the clinical study of SHR-1701, the signs and symptoms of patients with immune-related hepatitis, such as liver discomfort and abnormal increase of transaminase, will be monitored. Patients above degree 2 liver toxicity should receive high-dose hormone therapy. Patients with degree 2 immune-related hepatitis may suspend SHR-1701 and receive treatment, and patients with degree 3 or 4 immune-related hepatitis will discontinue SHR-1701.
- Patients with Grade 2 hepatitis should suspend the drug and have their AST and ALT levels monitored closely (test 1-2 times every week). If there is no improvement in level of transaminase for more than 1 week after drug withdrawal, start methylprednisolone (0.5-1 mg/kg) treatment, and gradually reduce the reduce by closely monitoring the levels of AST, ALT and bilirubin after several weeks.
- Patients with Grade 3 hepatitis should interrupt study treatment, and receive methylprednisolone (1-2 mg/kg) treatment immediately, and if there is no improvement 2-3 days after treatment, add mycophenolate mofetil.
- Patients with Grade 4 hepatitis should discontinue the study treatment permanently and are recommended to be hospitalized and receive intravenous treatment with methylprednisolone 2 mg/kg. If there is no improvement 2-3 days after treatment, add mycophenolate mofetil. If there is still no efficacy after treatment with two immunosuppressive drugs, please consult a hepatologist.
- **Immune-related abnormal thyroid function**
- Abnormal thyroid function may occur at any time of the study, so in SHR-1701 study, the thyroid function of patients will be tested regularly, and attention will be paid to the clinical symptoms of thyroid dysfunction. Patients with immune-related hyperthyroidism will be treated with high-dose cortisone/prednisone. Hormone replacement therapy will be used in case of hypothyroidism, but this does not apply to glucocorticoid.
- In the clinical study of SHR-1701, the signs and symptoms of patients with immune-related abnormal thyroid function will be monitored. Patients above degree 3 should receive high-dose hormone therapy, and those with degree 4 should discontinue SHR-1701 permanently.
- **Immune-related nephritis and renal failure**
- For patients with nephritis, renal failure caused by other reasons should be ruled out first. Then interruption or permanent discontinuance of investigational drug will be decided according to the severity of renal insufficiency, and other nephrotoxic drugs will be discontinued to start the treatment with methylprednisolone at 1-2 mg/kg.
- **Rheumatology toxicity**
- Patients with mild arthralgia may be treated with non-steroidal anti-inflammatory drugs, and if there is no improvement, treatment with low dose hormones (10-20 mg prednisone) can be considered. Patients with serious polyarthritis are recommended to receive treatment in the rheumatology department or consult a rheumatologist, and start the treatment with 1 mg/kg prednisone. Sometimes it is necessary to treat arthritis with infliximab or other anti-TNFα drugs.
- **Immune-related cardiotoxicity**
- In the clinical study of SHR-1701, the signs and symptoms of patients with immune-related myocarditis, such as dyspnea, palpitation, chest pain and precordial discomfort, will be monitored. Electrocardiogram, myocardial zymogram and echocardiography and other related examinations will be performed in the opinion of the investigator, and once the diagnosis of immune-related myocarditis is confirmed, the patient should receive treatment with high-dose methylprednisolone (1-2 mg/kg) and permanently discontinue the treatment with investigational drug.
- **Principle for treatment of other immune-mediated adverse reactions**
- In principle, depending on the severity of adverse reactions, SHR-1701 should be suspended, and when the severity of adverse events returns to ≤1 degree or the baseline level, reuse of SHR-1701 can be considered, in case of some serious degree 3 or life-threatening degree 4 adverse reactions, SHR-1701 should be discontinued permanently.
- For patients requiring the use of glucocorticoids, in this study, it is recommended to closely observe the adverse reaction caused by glucocorticoids (the severity is in direct proportion to dosage and time of medication), and preventive and treatment measures should be provided according to the medical practice and guidelines of testing facility.Table 11 The suggestions listed are for reference only (including but not limited to the following).

Table 11 Adverse reactions caused by glucocorticoids and suggestions for their prevention and treatment

| **Adverse reactions caused glucocorticoids** | **Preventive and treatment measures** |
| --- | --- |
| Gastrointestinal ulcer and/or bleeding | Use gastric mucosal protective agents, H2 receptor antagonists, proton pump inhibitors and other drugs. |
| Electrolyte disorder (for example, hypokalemia, hypernatremia) | Monitor electrolytes and actively correct electrolyte disorders (for example, low sodium and high potassium diet, oral or intravenous potassium supplementation if necessary) |
| Hyperglycemia, hypertension, hyperlipidemia, etc | Monitor blood glucose, blood pressure and blood lipid, and give hypoglycemic, antihypertensive and lipid-lowering treatments if necessary. |
| Osteoporosis, spontaneous fracture or osteonecrosis | Supplement active vitamin D3 and calcium, and treat osteoporosis if necessary. |
| Infections with bacteria, viruses, fungi, etc. | Give anti-infective therapy actively |
| Water and sodium retention such as congestive heart failure | Give appropriate diuretics to minimize the influence on electrolytes |
| Mental symptoms such as anxiety, excitement, euphoria or depression, insomnia | Give corresponding anti-anxiety, anti-depression and sleep-helping drugs with the guidance of psychiatrists |
| Muscle weakness, muscle atrophy, and slow wound healing. | Give high-protein diet, etc. |

##### Safety management rules for combination of BP102 with XELOX

For combination of BP102 with XELOX supportive treatment and care, refer to the instructions for use or clinical practice of the study site.

### Rescue treatment

NA

## Method to minimize bias

### Procedures of enrollment/randomization/blinding

Phase 3 of this study is designed to be a double-blind, placebo-controlled study, and subjects who have signed the informed consent form and meet the inclusion criteria are randomized to the test group and control group at a random ratio of 1:1. The stratification factors include PD-L1 expression (< 1% or ≥1%), location of primary tumor (left or right) and status of RAS gene (mutant or wild type).

In this study, subjects will be randomized through the randomization system, and eligible subjects will be randomized to the test group or control group at a ratio of 1:1, and the corresponding drug numbers will be obtained through the randomization system before drug dispensing. As far as possible, patients should receive the first dose of study treatment on the day of randomization.

SHR-1701 and placebo will be packaged in the same way so as to remain blind. SHR-1701 and placebo will be prepared by a specific study nurse, and during the study, the dispensing nurse should not participate in the administration to subjects as far as possible, and the drug should be administered by other study nurses. Subjects, investigator, and the staff of the sponsor who participate in the treatment or clinical evaluation of subjects or their designated personnel do not know the grouping.

### Evaluation of blindness

Sponsor and agent (except IDMC members), staff of study site and patients will remain blind to the drug dispensing information of the investigational drugs during the study.

In order to reduce the deviation, in the Phase 3 study, the imaging evaluation frequency of the test group and the control group will remain the same, and the imaging of tumor lesions will be evaluated based on the RECIST1.1 standard, and the primary efficacy endpoint (PFS) will be evaluated by the Independent Review Committee (IRC) in a blind state. The final analysis strategy, including definition of data censoring rules in advance, will be determined before locking of the database of primary efficacy endpoint analysis. The efficacy will be analyzed only at the time point specified in the protocol.

### Unblinding

This study remains blind to subjects, investigator or authorized persons, study site and sponsor, until the primary endpoint of the study is achieved. In order to minimize the possibility of deviation, the randomized treatment information will be kept confidential throughout the study and will not be disclosed to the blind team until the study database is locked.

Treatment randomization information will be stored in the random system of the third party, and the login permission will be strictly controlled.

After it is considered that the established database is correct and free of error upon blind review, a blind review report will be issued and the database will be locked. The data should be kept properly for filing after locking of database, and the blind code and database will be submitted to the statistical experts for statistical analysis.

The identification information of treatment will be unblinded only when necessary for the benefit of the subjects. Unless it is necessary, the subjects should not be unblinded as far as possible.

When the investigator should determine the drugs used by the subjects and the dosage in emergency, the responsible investigator of the study site will make an application, and the sponsor’s medical director and the principal investigator will jointly decide whether unblinding is necessary. The investigator will unblind the subjects, and report the unblinding to the sponsor. Before unblinding, the investigator must enter the toxicity grade of the observed adverse events, the relationship with the investigational drugs and the reasons in the medical records and other documents.

Subjects whose treatment assignment is unblinded by the investigator and/or non-study treatment physicians must discontinue the investigational drugs, but should continue to be monitored during the study.

Once in case of unblinding, the situation at the time of unblinding (such as date, reason and those who are responsible for unblinding) must be recorded immediately, and notified to the CRA of the sponsor as soon as possible.

The study will be unblinded overall when the primary endpoint is achieved. The blinding code will be informed by the sponsor to the investigator, who will inform it to the subjects. Subjects in the test group will continue to receive treatment with SHR-1701 and capecitabine and BP102, and subjects in the control group will receive capecitabine and BP102, until disease progression, toxicity intolerance, start of new anti-tumor treatment, withdrawal of informed consent or the subject should withdraw from the study treatment in the opinion of the investigator. The maximum time of administration is 2 years. Subjects in the control group will no longer have ADA and PK blood samples collected.

# Study procedures

The tests and steps required by the protocol should be ensured with the best possible effort as planned. However, unscheduled events can occur from time to time that are beyond the investigator's control, making the test difficult to perform. In these cases, the investigator should take all necessary measures to protect the safety and interests of the subjects. When a protocol-required test can not be performed, the investigator should record the reason. In addition, the investigator should inform the study team of unexpected situations in a timely manner.

## Screening

Subjects who have failed previous screening and are not randomized will be allowed to be re-screened in this study, and at re-screening, the subjects must re-sign the ICF and re-register to obtain a new subject number. Each subject can be re-screened only once (if the examination in the screening period is not completed within 28 days, re-screening is required).

Unless otherwise specified, the following screening procedures must be completed within 28 days prior to the first dose:

- Obtaining written informed consent signed by the subject or guardian/notary witness;
- Collection of medical history and demographic data (such as sex, date of birth and ethnicity);
- Collection of tumor medical history
- Tumor history: pathological diagnosis, previous imaging, clinical diagnosis, staging, recurrence or metastasis date, genetic test results, etc.;
- Tumor surgical history: surgery name, site, date, method of surgery, etc.;
- Radiotherapy history: radiotherapy site, dosage, start and end dates, etc.
- Tumor medication history: protocol, start and end dates, number of cycles, drug name, dose, etc.
- Other tumor treatment histories: treatment with traditional Chinese medicine, local ablation, interfering treatment and all other tumor related treatment histories;
- Collection of other past medical histories: including history of drug allergy, history of diagnosis and treatment of other concomitant diseases, and history of tumors other than the disease under study;
- Collection of past medication history; collection of all previous medications within 30 days before signing of informed consent form;
- Virological examination before collection of blood samples (within 14 days before the first dose);
- Necessary imaging: Tumor imaging examination: CT or MRI of chest and abdomen (including pelvic cavity) (both enhanced,and plain scanning can be used instead except for the prohibition of contract agent), MRI of brain is required when in case of suspicion or diagnosis of brain metastasis (CT can be used instead except for prohibition of MRI, and both are enhanced, and plain can be used instead except for the prohibition of contract agent), and bone scanning is only performed when clinically indicated; in the screening period, the imaging results obtained before signing informed consent form can be used for tumor baseline assessment as long as they meet the requirements of RECIST1.1, the baseline imaging can be extended to within 4 weeks prior to the first dose, and the time of bone scanning examination can be relaxed to within 42 days before the first dose;
- Evaluation of current concomitant medications and therapies;
- Evaluation of current symptoms/adverse events;
- Collection of subjects’ tumor tissue samples: the newly acquired tissues are preferred, or the archived tumor tissues will be collected
- Test of biomarkers: The biomarkers (RAS gene type, PD-L1 expression, etc.) in Phase 2 will be tested by the study site or using the previous test reports of subjects; RAS gene type and PD-L1 expression should be tested in the central laboratory before Phase 3 randomization.
- Initial verification of inclusion and exclusion criteria;

Except imaging, written informed consent must be obtained before any medical operation specified in the study, but the CT/MRI scan results obtained before signing the informed consent to participate in this study can be used for tumor evaluation in the screening period if they meet the evaluation criteria (the test time must be within 28 days before the start of study treatment).

The following screening procedures must be completed within 7 days prior to the first dose, and pregnancy study should be completed within 3 days prior to study medication:

- Careful and comprehensive physical examination: height, weight, organs
- ECOG score (Attachment 2 )
- Vital signs: respiration, blood pressure, pulse, body temperature, etc.
- Necessary laboratory tests:
- Hematology test: including red blood cell count, hemoglobin, platelet count, white blood cell count, neutrophil count and lymphocyte count;
- Urinalysis: urine white blood cells, red blood cells and urine protein.
- Fecal occult blood;
- Blood chemistry: ALT, AST, GGT, total bilirubin, direct bilirubin, AKP, blood urea nitrogen or urea (preferably blood urea nitrogen), total protein, albumin, creatinine, blood sugar, lactate dehydrogenase, K+, Na+, Ca2+, Mg2+, Cl-
- Coagulation function: APTT, PT, FIB and INR
- CEA
- Thyroid function test: TSH, FT3, FT4;
- 12-lead ECG: heart rate, PR interval, QT interval, QTcF
- Echocardiography
- Blood HCG test: within 3 days prior to the first dose
- Adverse event
- Concomitant medications
- Verification of inclusion and exclusion criteria

## Treatment period

The treatment period will start from randomization of subjects (from enrollment for Phase 2), and the first administration should start within 2 days after randomization.

All tests and assessments (except imaging) should be completed within 3 days before administration. The following assessments should be completed before enrollment every cycle, if the screening phase is completed within 7 days before the first dose, no retest is required at the first dose.

**The following tests should be completed before medication at each cycle:**

- ECOG score;
- Targeted physical examination
- Vital signs: respiration, blood pressure, pulse, body temperature
- Necessary laboratory tests:
- Hematology test: including red blood cell count, hemoglobin, platelet count, white blood cell count, neutrophil count and lymphocyte count;
- Blood chemistry: ALT, AST, GGT, total bilirubin, direct bilirubin, AKP, blood urea nitrogen or urea (preferably blood urea nitrogen), total protein, albumin, creatinine, blood sugar, lactate dehydrogenase, K+, Na+, Ca2+, Mg2+, Cl-
- Urinalysis: urine white blood cells, red blood cells and urine protein.
- Fecal occult blood;
- Thyroid function test: TSH, FT3, FT4;
- 12-lead ECG: heart rate, PR interval, QT interval, QTcF
- Record the AEs reported by the subjects or investigator
- Concomitant medications
- Record subject compliance
- Distribute, verify and recover the subject log on Day 1 of medication every cycle (only distribute the subject log before the first dose);

**In addition, the following tests should be completed before administration every two cycles:**

- CEA

**Imaging requirements during the treatment period:**

During the treatment period, imaging will be performed every 6 weeks (±7 days) in the first 48 weeks and every 12 weeks (±7 days) thereafter. In case of suspicion of disease progression, unscheduled imaging can be performed. For bone metastases, bone scanning is only needed when the evaluation results of other lesions are CR and it is necessary to confirm whether all bone metastases have disappeared or clinically indicated. The allowable window period of imaging examination is ±7 days; the imaging examination conditions should be the same with those at the baseline (including scanning thickness, contract agent, etc.) The imaging time will not be adjusted due to the delay in the start of administration cycle.

**PK and immunogenicity blood collection**

All subjects should undergo PK and immunogenicity blood sample collection. The time points of blood collection are as follows: On C1D1, C2D1, C4D1, C7D1 and Day 1 of every 6 cycles thereafter, blood samples should be collected within 0.5 hour before the administration of SHR-1701/ placebo; in case of suspension of the administration of SHR-1701/ placebo, the scheduled pre-dose PK and immunogenic blood samples should still be collected as much as possible, and for subjects who cannot pay a return visit, the blood samples should be collected at the next visit. In case of permanent discontinuation of SHR-1701/ placebo (regardless of whether other drugs are discontinued) and it has been more than 28 days since the last dose, subjects should pay a return visit as soon as possible and have blood samples collected. Subjects may select to have blood samples collected either at the end-of-(SHR-1701/placebo) treatment visit or 30 days (±7 days) after the last dose of SHR-1701/placebo, and then have blood samples collected 60 days (±7 days, if applicable) and 90 days (±7 days, if applicable) after the last dose of SHR-1701/placebo, respectively. At each time point, about 6 mL of venous blood will be collected for PK and immunogenicity tests of SHR-1701. PK and immunogenicity blood samples should be collected as scheduled, but for the need of PK and immunogenicity assay, unscheduled blood samples may be collected.

## End-of-treatment/study treatment withdrawal visit

The visit will be completed when it is confirmed that the subjects should terminate the study treatment, and if the following assessments and tests are not performed within 7 days before withdrawing from the study treatment (except for imaging), it should be completed at the time of withdrawing from the study treatment visit.

- ECOG score;
- Vital signs: pulse, respiratory rate, body temperature and blood pressure.
- Careful and comprehensive physical examination: ECOG PS score, body weight and organs;
- Necessary laboratory tests:
- Hematology test: including red blood cell count, hemoglobin, platelet count, white blood cell count, neutrophil count and lymphocyte count;
- Urinalysis: urine white blood cells, red blood cells and urine protein.
- Fecal occult blood;
- Blood chemistry: ALT, AST, GGT, total bilirubin, direct bilirubin, AKP, blood urea nitrogen or urea (preferably blood urea nitrogen), total protein, albumin, creatinine, blood sugar, lactate dehydrogenase, K+, Na+, Ca2+, Mg2+, Cl-
- Coagulation function: APTT, PT, FIB and INR
- CEA
- Thyroid function test: TSH, FT3, FT4;
- 12-lead ECG: heart rate, PR interval, QT interval, QTcF
- Echocardiography
- Blood HCG test
- PK and immunogenicity blood collection (at the end of SHR-1701/placebo treatment visit and at 30 days (±7 days) after the last dose of SHR-1701/placebo, a visit can be selected for PK and immunogenicity blood collection)
- Imaging: If no imaging has been performed within 4 weeks before withdrawing from the study treatment visit, an imaging examination should be performed at the time of withdrawing from the study treatment visit.
- Record the AEs reported by the subjects or investigator
- Concomitant medications
- Recovery and verification of subject log

## Follow-up period

### Safety follow-up visit

Following the end-of-treatment visit (including subjects who completed the end-of-treatment/withdrawal visit), subjects will enter the follow-up period. 30 days after the last dose of study drug, regardless of whether or not the subject starts a new anti-tumor therapy, the subject needs to return to the study site for safety follow-up and complete the following safety assessment indicators. If a subject is unable to resume study medication due to AE or other reasons, the date of the last medication is determined as the date of the last dose. If this date is more than 30 days from the date when it is judged that the subject needs to withdraw from study treatment, there is no need to record as protocol deviation, but it is recommended that the subject return to the study site as soon as possible for a safety visit. 60 days (±7 days) and 90 days (±7 days) after the last study dose, the patient should pay a safety follow-up visit to the study site as far as possible, and have PK/ immunogenicity blood samples taken (if applicable); a patient who indeed cannot go to the study site should conduct a telephone visit to collect survival information, subsequent anti-tumor treatment, concomitant medication/treatment, AE/SAE; if it is less than 45 days from the completion of the first safety visit to the end of the safety follow-up visit, the safety visit can be conducted only within ±7 days of the last day of the safety follow-up period.

- Hematology test: red blood cell count, hemoglobin, platelet count, white blood cell count, neutrophil count and lymphocyte count;
- Blood chemistry: ALT, AST, GGT, total bilirubin, direct bilirubin, AKP, blood urea nitrogen or urea (preferably blood urea nitrogen), total protein, albumin, creatinine, blood sugar, lactate dehydrogenase, K+, Na+, Ca2+, Mg2+, Cl-;
- Thyroid functions: TSH, FT3, FT4;
- PK and immunogenicity blood collection (at the end of SHR-1701 treatment visit and at 30 days (±7 days) after the last dose of SHR-1701, a visit can be selected for PK and immunogenicity blood collection; with the consent of subjects, it is suggested that subjects should have PK and immunogenicity blood samples collected at the study site 60 (±7 days) and 90 (±7 days) after the last dose of SHR-1701);
- Record the AEs reported by the subjects or investigator;
- Concomitant medications;

### Survival follow-up

The survival follow-up visit will be conducted after completion of the safety follow-up period. The investigator must pay a survival follow-up visit to the subject every two months, until subject death, loss to follow-up, study termination by the sponsor, or other criteria for end of study are met (whichever occurs first). The investigator can inquire the subject himself/herself, his/her family members or a local physician via mail or telephone, and collect the survival status (date of death and cause of death), subsequent anti-tumor treatment information (including systemic anti-tumor drug therapy, surgery, radiotherapy), and disease progression information after next-line treatment of the subject, etc. Each survival follow-up needs to be recorded in the original medical record.

In addition to safety follow-up and survival follow-up, in the absence of radiologically confirmed disease progression, tumor assessments should continue to be performed regardless of whether the subject has discontinued study treatment, until radiologically confirmed disease progression is obtained, or until subject death, loss to follow-up, withdrawal of informed consent, or study termination by the sponsor, whichever occurs first.,

All treatment-related toxicities must be followed until resolution, return to baseline, or deemed irreversible. AEs should be reported and recorded as required during safety follow-up.

## Unscheduled visits

If the subjects required an unscheduled follow-up visit due to AEs during the study, the following items should be recorded:

- Record concomitant medications;
- Record AEs;
- Record relevant examinations performed (including imaging examinations, if any).

## Subject's withdrawal from the study or permanent discontinuation of study treatment

### Withdraw from the study

Subjects can voluntarily withdraw from the study at any time, or are required to withdraw from the study by the investigator or sponsor for safety or behavior reasons or because they cannot comply with the time or steps required in the study protocol at the study site where they are located.

If the subjects fail to return to the site for follow-up as agreed, every effort should be made to contact them. In any case, if possible, every effort should be made to record the subjects’ outcome. The investigator should consult the subjects for the reason for withdrawal, and require the subjects to return all investigational drugs not used, require the subjects to the site for final visit, and follow up any unsolved adverse event (AE).

If the subjects refuse to pay a further visit at the study site, their survival should still be followed up and collected, unless the subjects withdraw the consent of disclosing further information or being further contacted. In this case, no study evaluation should be performed, nor should any data be collected.

For subjects who are lost to follow-up or die, the procedure to withdraw from the study will no longer apply.

### Permanent discontinuation of study treatment

Discontinuance of study treatment does not mean withdrawal from the study. Subjects who discontinue study treatment must continue to complete the remaining study visits as required by the protocol. Possible reasons for discontinuance of study treatment include:

- The subjects request to discontinue treatment with investigational drugs;
- Disease progression which requires discontinuance
- Pregnancy of subjects during the study;
- Major non-compliance with study treatment;
- Any clinical adverse events, abnormal laboratory tests or other medical conditions which cause that subjects cannot benefit from further medication;
- General deterioration of health status that prevents continued participation in the study;
- Major protocol deviation, for example, the subjects no longer meet the condition of further participation in the study after enrollment;
- Loss to follow-up;
- Death of subjects;
- Other reasons that the study treatment cannot be continued in the opinion of the investigator;
- Expiration of 2 years of use of SHR-1701/ placebo /BP102/ capecitabine;
- Study discontinuance of study by the sponsor.

Every effort must be made to complete the protocol-specified efficacy and safety examination at the time of discontinuance of study treatment, complete safety follow-up, and record adverse events (AEs) and outcomes in full. The investigator can recommend or provide new or alternative treatments to the subject based on his/her actual condition.

For subjects who are lost to follow-up or die, the procedure to discontinue study treatment will no longer apply.

### Loss to follow-up

Subjects will be considered lost to follow-up if they fail to return to the site for scheduled visits for more than 3 times and the site is unable to reach them.

If the subject fails to return to the site for necessary study visits, the following measures must be taken:

- The site attempts to contact the subject and reschedule the missed visit as soon as possible and informs the subject of the importance of having the visit performed on time, and determines if the subject is willing to and/or should continue the study.
- Before a subject is deemed lost to follow-up, the investigator or his/her designee must make every effort to contact the subject (for example, contact by telephone for 3 times, email if necessary and social medial tools). All attempts to make contact should be documented in medical files.
- If a subject remains unreachable, he/she will be considered withdrawn from the study, with the primary reason for withdrawal being loss to follow-up.

## Premature termination or suspension of a study

The study may be terminated early or suspended if there are sufficient reasons. This may be due to decisions from the regulatory authority, opinion changes by the ethics committee, recommendations of DMC, efficacy or safety issues of the investigational drug, or based on the sponsor's judgment. In addition, Jiangsu Hengrui Pharmaceutical Co., Ltd. (hereinafter referred to as"Hengrui") reserves the right to stop the research and development of SHR-1701 at any time.

The party who decides to suspend/terminate the study will give a written notice documenting the reason for study termination or suspension to the investigator, the sponsor, and the regulatory authority. If the study is terminated early or suspended, the investigator should immediately inform the ethics committee and the sponsor and provide relevant reasons.

Reasons for premature termination or suspension of the study may include:

- Identified unexpected, significant, or unacceptable risk to subjects.
- Available efficacy results support early termination of the study.
- The compliance with protocol requirements is low.

After the study is terminated early or suspended, the investigator should contact all participating subjects and the hospital pharmacy immediately as requested by the sponsor, all study materials must be collected and all eCRFs should be completed to the maximum extent possible.

The study can continue once the safety of the drug causing suspension, protocol compliance and other issues mentioned above have been resolved and agreed by the sponsor, ethics committee or regulatory authority.

## Definition of End of Study

The end of study is defined as follows:

Expiration of 2 years after the first dose of the last subject.

Besides, the sponsor may decide to terminate the study at any time.

## Further medication at the end of study

NA

# Evaluation

## Efficacy evaluation

Progression-free survival (PFS): the time from the start of randomization to the date of first record of objective tumor progression or the time of death for any reason, whichever occurs first. The independent review committee (IRC) will conduct independent imaging assessment of the primary endpoint. For details, refer to the IRC Charter. The secondary endpoints are subject to the assessment by the investigator. If PFS has not been obtained as of the deadline of analysis, the data will be censored. For the censoring rules, see the statistical analysis plan (SAP).

Overall survival (OS): the time from the start of randomization dose to the death of subjects for various reasons. If OS has not been obtained as of the deadline of analysis, the data will be censored. For the censoring rules, see the SAP.

Objective response rate (ORR): defined as the proportion of subjects with CR and PR in the number of subjects who take the drug at least once in each treatment group. Best overall response (BoR): defined as best response indicator between the randomization date and the objectively recorded progression date or the subsequent anti-tumor treatment date (whichever comes first). For the subjects who have no recorded progression or receive subsequent anti-tumor treatment, BOR will be determined according to all relief evaluation results.

Duration of response (DoR): the time from the first PR or CR to the first PD or death. If PD or death has not been obtained as of the deadline of analysis, the data will be censored. For the censoring rules, see the SAP.

Disease control rate (DCR): the proportion of subjects with CR, PR and SD in the number of subjects who take the drug at least once in each treatment group. DCR is defined as best response indicator between the randomization date and the objectively recorded progression date or the subsequent anti-tumor treatment date (whichever comes first). For the subjects who have no recorded progression or receive subsequent anti-tumor treatment, DCR will be determined according to all relief evaluation results.

Analysis of the relationship between colorectal cancer RAS mutation and efficacy by biomarkers.

The reference standard for imaging assessment in this study is RECIST1.1 criteria (Attachment 1 ). Refer to the study flowchart for the imaging requirements and frequency.

All subjects' documentation and radiographic can be used for validation and future peer review.

### Blind independent review committee (BIRC)

In this study, the BIRC will review all tumor images independently, and determine the tumor response and disease progression according to RECIST v1.1 criteria. The tumor assessment results of BIRC will be used for the analysis and report of study results. All decisions made by the investigator during the study will be based on the assessment of imaging examination by the investigator, the clinical condition of the subjects and the comprehensive assessment of related examinations. Each testing facility will submit specific imaging image files to the independent review committee during the study or at the request of the sponsor. Detailed rules and guidelines for tumor imaging evaluation by BIRC will be described in a separate independent reading charter and image acquisition guidelines.

## Safety evaluation

Safety evaluations include the collection of AEs, serious adverse events (SAEs), vital signs and physical examinations, 12-lead ECG, and laboratory tests, including pregnancy test and confirmation of combined treatment.

### Pregnancy test

Female subjects with childbearing potential will receive serum pregnancy test within 3 days prior to the first dose. Subjects with negative test should take appropriate contraceptive measures. If a subject has positive test, the subject fails to be screened. Enrolled subjects should receive serum pregnancy test again when withdrawing from the study treatment visit.

### Adverse event

The evaluation of adverse events (AEs) includes type, occurrence, severity (graded according to NCI-CTCAE version 5.0), occurrence and end time, whether they are serious adverse events, relationship with the investigational drug, and outcome.

AEs occurring during the study, including signs and symptoms during the screening phase, will be recorded in the AE page of CRF.

### Laboratory safety evaluation

Refer to the study flowchart for details.

### Vital signs and physical examination

Refer to the study flowchart for details.

### 12 lead ECG

Refer to the study flowchart for details.

## Pharmacokinetic evaluation

### Blood sample collection for SHR-1701 PK analysis

Collect the blood samples as shown in the test flow chart into appropriately marked test tubes for PK analysis.

Every effort should be made to obtain pharmacokinetic samples at the scheduled and relative time of administration. The exact sampling time will be fully noted in the CRF. If, for any reason, the sampling cannot be completed at the scheduled time, the missed sampling may be collected at other times after agreement between the investigator, the subject, and the sponsor.

The PK sample of SHR-1701 will be detected by the validated enzyme-linked immunosorbent assay (ELISA). The PK samples will be analyzed according to the SOP of central laboratory. The laboratory manual will provide information on the collection, processing, storage, and transport of blood samples.

## Immunogenicity

Serum samples will be collected as shown in the study schedule for immunogenicity analysis of SHR-1701.

The anti-SHR-1701 antibody (ADA) is detected by the validated electrochemiluminescence method based on agarose affinity purification, the anti-SHR-1701 neutralizing antibody (NAb) is detected by the validated electrochemiluminescence method based on competitive ligand binding, and the samples are analyzed according to the SOP of the central laboratory. The laboratory manual will provide information on the collection, processing, storage, and transport of blood samples.

The study site must handle and transport the immunogenic samples according to the corresponding regulations to ensure the integrity of samples. Any deviation related to the handling procedure of immunogenic samples (for example, sample collection and handling procedures, temporary storage or transportation conditions), including any measures taken, must be recorded and reported to the sponsor. The sponsor my determine whether the integrity of the sample is damaged depending on the specific circumstances. Any deviation from the handling procedure that damages the sample integrity will be considered as a protocol deviation.

As part of understanding the immunogenicity of the investigational drug, the data will be used for internal exploration objectives and will not be included in CSR.

# Adverse event reporting

## Adverse event (AE)

### Definition of AE

AE refers to all adverse medical events that occur after the subject receives the investigational drug, which can be manifested as symptoms, signs, diseases, or abnormal laboratory tests, but does not necessarily have a causal relationship with the investigational drug. The information of AEs will be collected from the signing of informed consent form by subjects. The collection time will start from the signing of the informed consent form until 90 days after the last dose of SHR-1701/ placebo or 30 days after the last dose of chemotherapy/BP102 therapy (whichever is longer); if a patient starts a new anti-tumor treatment during the AE collection period, only the AE related to the investigational drug will be collected after the new anti-tumor treatment; AE includes but not limited to:

1. Worsening of pre-existing (before entering the clinical study) medical conditions/diseases (including worsening of symptoms, signs and laboratory abnormalities);
2. Any new AE: any new adverse medical condition (including symptoms, signs and newly diagnosed diseases);
3. Abnormal clinically significant laboratory results.

The investigator should record in detail any AE that occurs in the subjects, including: the name of AE, description of all related symptoms, time of occurrence, severity, relationship with the test drug, duration, measures taken for the investigational drug, and final results and outcomes.

### Criteria for judging the severity of AEs

Refer to the grading criteria of drug AE in NCI-CTC version AE 5.0. If an AE not listed in NCI-CTC AE version <5.0> occurs, the following criteria can be applied:

Table 12 Criteria for judging the severity of adverse events

| **Grade** | **Clinical description of severity** |
| --- | --- |
| 1 | Mild; asymptomatic or minimal symptoms; clinical or laboratory abnormalities only; intervention not indicated |
| 2 | Moderate; minimal, local or noninvasive intervention indicated; limited age-appropriate instrumental activities of daily living (ADL); ADL refer to preparing meals, shopping, using the telephone, counting money, etc. |
| 3 | Severe or medically significant but not immediately life-threatening; hospitalization or prolonged hospitalization indicated; disabling; limiting self-care ADL. Self-care refers to bathing, dressing, undressing, eating, using toilet, taking medications, etc., not being bedridden |
| 4 | Life-threatening; emergency treatment indicated |
| 5 | Any AE leading to death |

Abbreviations: AE = adverse event; ADL=activities of daily living.

### Judgment of relationship between AE and investigational product

Causality judgment between an AE and the investigational drug refers to whether there is a reasonable possibility that investigational drug causes or contributes to the AE as determined by a comprehensive assessment; judgment factors include whether there is a reasonable temporal sequence between the occurrence of the AE and the administration of investigational drug, the characteristics of investigational drug, the toxicological and pharmacological effects of investigational drug, the use of concomitant medications, the subject's underlying diseases, medical history, family history, and dechallenge and rechallenge reactions. Generally, facts (evidence) or arguments to determine causality should be provided.

Causal relationship between AE and any investigational drug is evaluated by the investigator as "definitely related", "possible", "uncertain", "unlikely", and "not related".

## SAEs

### Definition of SAE

SAE refers to any of the following adverse medical events occurring in subjects after receiving any dose of the investigational drug:

- An event leading to death;
- A life-threatening event (defined as an event that places the subject at risk of death at the time of occurrence; however, this does not include an event that, had it occurred in a more severe form, might have caused death);
- An event requiring hospitalization or prolonged hospitalization;
- An event leading to permanent or significant disability/loss of function;
- Congenital anomaly or birth defect;
- Other important medical events.

Medical and scientific judgment must be used to determine whether other situations should be considered as SAEs, such as important medical events that may not be immediately life-threatening or result in death or hospitalization but may jeopardize the patient or may require intervention to prevent one of the outcomes listed in the definition above. For example, allergic bronchospasm treated in the emergency room or at home, cachexia or convulsion that does not result in hospitalization, and development of drug dependence or drug abuse.

### Hospitalization

AEs leading to hospitalization (even if less than 24 hours) or prolonged hospitalization in the clinical study should be considered SAEs. Hospitalization does not include the following:

- Rehabilitation facility;
- Sanatorium;
- Admission to conventional emergency room (less than 24 hours);
- Day surgery (e.g. outpatient/day/ambulatory surgery).

Hospitalization or prolonged hospitalization not associated with worsening of AE is not an SAE. For example, the following are not considered a SAE:

- Admission to the hospital for treatment of pre-existing diseases, which is not related to a new AE and does not involve an exacerbation of pre-existing diseases (e.g., for laboratory abnormalities that have already existed before the deagnostic test and persist to date);
- Hospitalization for management reason (for example, annual routine physical examination);
- Hospitalization specified in the study protocol during the clinical study (for example, operation as required in the study protocol);
- Elective hospitalization not related to an AE (e.g., elective cosmetic surgery);
- Pre-planned treatment or surgical procedure (should be recorded in the whole study protocol and/or the baseline data of individual subject);
- Hospitalization solely for use of blood products.

Diagnostic or therapeutic invasive (e.g. surgery), non-invasive procedures should not be reported as an AE. However, when the disease condition leading to these procedures meets the definition of AE, it should be reported. For example, acute appendicitis occurring during the reporting of AE should be reported as an AE, and appendectomy should be recorded as the treatment method of this AE.

### Disease progression and death

Disease progression is defined as the deterioration of the subject's condition caused by the indications of the study, including radiographic progression and progression of clinical symptoms and signs. New metastases from the primary tumor, or progression of existing metastases, are considered as disease progression. Events that are life-threatening, require hospitalization or prolonged hospitalization, or result in permanent or significant disability/loss of function due to signs and symptoms of disease progression are not reported as SAEs. Radical surgical treatment due to successful transformation is not reported as an SAE; if there is any uncertainty as to whether an SAE is due to disease progression, it should be reported as an SAE.

Any subject who dies during the safety follow-up period in the clinical study must be reported as an SAE, regardless of whether the investigator assesses it as possibly related to disease progression, or whether the subject has received other anti-tumor therapies. The term "death" should not be used as an SAE term, but as an outcome of an event, and an event leading to death should be recorded as an SAE. A medical conditions/disease causing or leading to death (including worsening of symptoms and signs) will be recorded as an SAE term in the eCRF and reported as an SAE. If the cause of death can not be determined at the time of reporting, the SAE term will be recorded as "unexplained death".

If a subject dies due to disease progression as evaluated by the investigator, Grade 5 events due to disease progression should be recorded in the eCRF and reported as an SAE; if death due to disease progression cannot be attributed to a specific medical event, "tumor progression" graded as Grade 5 should be recorded in the eCRF and reported as an SAE, and the investigator should provide evidence of death due to disease progression (e.g., imaging changes suggestive of tumor development or progression, clinical deterioration related to the disease process).

### Other anti-tumor therapies

If a subject starts other anti-tumor therapies before the end of the safety follow-up period, for a non-fatal SAE, unless it is suspected to be related to the investigational drug, the reporting period ends until the start of new anti-tumor therapy. If the death occurs during the safety follow-up period, it must be reported as an SAE regardless of whether the subject receives other treatments.

### Reporting system for SAEs

In case of an SAE, whether it is an initial report or a follow-up report, the investigator must immediately complete Hengrui 's Clinical Study Serious Adverse Event/Adverse Event of Special Interest Report Form, sign and date, immediately notify the sponsor within 24 hours after the investigator' s awareness, and report to relevant organizations in a timely manner according to local regulations.

SAEs occurring after the safety follow-up period should be collected for those suspected to be related to the investigational drug. For an SAE, the symptoms, severity, relationship with the investigational drug, occurrence time, treatment time, measures taken, follow-up time and method, and outcome should be recorded in detail. If the investigator considers an SAE not related to the investigational drug but potentially related to study conditions (such as termination of original treatment, or comorbidities during the study), this relationship should be described in detail in the narrative section of the SAE report form. If the intensity of an ongoing SAE or its relationship with the test drug changes, a follow-up report should be submitted immediately. Any misinformation of the previously SAEs in the opinion of the investigator may be corrected, revoked or downgraded in the follow-up report and reported according to the SAE reporting procedure.

The sponsor's email address of this project for receiving SAEs (as well as SIEs and pregnancy) reports is: hengrui_drug_safety@hengrui.com.

## AE of special interest (SIE)

For the reporting of AEs of special interest (SIEs) as defined in the clinical study protocol, Hengrui's Clinical Study Serious Adverse Event/Adverse Event of Special Interest Report Form should be completed within 24 hours of the investigator's awareness and reported to the sponsor. If it is also an SAE, it should be reported to relevant organizations following the SAE reporting process.

SIEs in this study include:

- Abnormal liver function test (potential drug-induced liver injury)
- ≥ Grade 3 infusion reaction;
- Other ≥ Grade 3 immune-mediated adverse events;

### Abnormal liver test

Abnormal AST and/or ALT levels concurrent with an abnormal elevation in total bilirubin level that meet the (1) (2) (3) conditions in Table 13 with no other causes leading to abnormality, should always be reported as an SIE; if it conforms to the SAE definition, it should also be reported following the SAE reporting process.

Table 13 Judgment criteria for abnormal liver function tests

| **Conditions met** | **Judgment criteria** |
| --- | --- |
| 1. Abnormal ALT or AST | Normal at baseline: ALT or AST ≥ 3 × ULN during the treatment period;  Abnormal at baseline: ALT or AST ≥ 2 × baseline level and value ≥ 3 × ULN during the treatment period; or value ≥ 8 × ULN. |
| 1. Abnormal TBIL | Normal at baseline: TBIL > 2 × ULN during the treatment period;  Abnormal at baseline: TBIL increase > 1 × ULN **or** its value > 3 × ULN during the treatment period. |
| 1. No hemolysis and alkaline phosphatase < 2 × ULN (or no information available) | |

Abbreviations: ALT = alanine aminotransferase, AST = aspartate aminotransferase, TBIL = total bilirubin, ULN = upper limit of normal.

If a subject has abnormal AST and/or ALT levels concurrent with an abnormal elevation in total bilirubin level during the treatment or follow-up period, the subject should return to the site for examination and assessment as soon as possible (preferably within 48 hours) after learning of the abnormal results. Relevant liver function laboratory tests, detailed medical history and physical examination should be included, and the possibility of liver tumor (primary or secondary) should be considered.

Liver function laboratory tests may include albumin, creatine kinase, total bilirubin, direct and indirect bilirubin, gamma-glutamyltransferase, prothrombin time (PT)/international normalized ratio (INR), and alkaline phosphatase, in addition to repeat testing of AST and ALT. Detailed medical history collection recommendations include: history of alcohol consumption, acetaminophen, soft drugs, various supplements, family medical history, occupational exposure, sexual behavior history, travel history, history of contact with jaundiced patients, surgery, blood transfusion, history of liver disease or allergic diseases. Further tests may also include the testing of acute infection with hepatitis A, B, C, D, and E and liver imaging examination (e.g., biliary tract).

## Pregnancy

If a female subject becomes pregnant during the clinical study, the subject must discontinue study treatment and withdraw from the study; if the partner of a male subject becomes pregnant during the clinical study, the subject can continue the clinical study. The investigator should complete the Hengrui's Clinical Study Pregnancy Report/Follow-up Form and report it to the sponsor within 24 hours after learning of the pregnancy event.

The investigator should follow up the pregnancy until the final outcome (including any premature termination of pregnancy or delivery), and the delivery should be followed up until 1 month after the labor, and the pregnancy outcome should be reported to the sponsor. If the pregnancy outcome meets the criteria of SAE (e.g., ectopic pregnancy, spontaneous abortion, intrauterine fetal death, neonatal death, or congenital anomaly), it needs to be reported following the SAE procedures.

If a subject experiences a concurrent SAE during pregnancy, the SAE reporting procedure must also be followed.

## Follow-up visit of AEs/SAEs

The information of AEs will be collected from the signing of informed consent form by subjects. The collection time will start from the signing of the informed consent form until 90 days after the last dose of SHR-1701/ placebo or 30 days after the last dose of chemotherapy/BP102 therapy (whichever is longer); if a patient starts a new anti-tumor treatment during the AE collection period, only the AE related to the investigational drug will be collected after the new anti-tumor treatment (Table 14);

At each study visit, the investigator should assess whether the subject experiences an adverse event. All adverse events should be followed up until resolution of symptoms, or clinically relevant changes in laboratory values return to baseline and/or ≤ Grade 1, or are reasonably explained (e.g., loss to follow-up, death), or the event is conclusively confirmed as unrelated to the investigational drug or the study procedure by the end of the safety follow-up period. Every effort should be made to ensure that the subject achieve the best outcome and obtain a definitive causality assessment.

All AEs should be documented in detail on the AE page of the eCRF, including: event term, start and end dates, severity, seriousness, causal relationship between the event and the investigational drug, actions taken on the investigational drug, and final outcome.

Table 14 Collection of AEs/SAEs/SIEs/pregnancy events

| **Term** | **Collection requirements** |
| --- | --- |
| From the time the subject signs the ICF until the end of the safety follow-up period | All AEs/SAEs/SIEs/pregnancy events  * If new anti-tumor therapy is started before the end of safety follow-up visit, non-serious adverse events unrelated to the investigational drug will not be collected.  Pregnancy event: collected after the first dose of the investigational drug |
| After the term above | SAEs/SIEs related to the investigational drug |

Abbreviations: AE=adverse event; SAE=serious adverse event; SIE=adverse event of special interest

*Safety follow-up visit is the period from the signing of informed consent form by the subjects to Day 90 after the last dose; if a patient starts a new anti-tumor treatment during the AE collection period, only the AE related to the investigational drug will be collected after the new anti-tumor treatment;

# Clinical monitoring

The monitor must follow the Good Clinical Practice (GCP) and Standard Operating Procedure (SOP), visit the study site regularly or according to the actual situation, supervise the conduct and progress of clinical study, check and confirm that all data records and reports and case report forms are correct and complete, and are consistent with the original data, so as to ensure that clinical study is carried out according to the clinical protocol. The investigator should actively cooperate with the monitor. The specific contents of the monitor include:

1. Before the study, verify that the study undertaker has appropriate conditions, including staffing and training, well-equipped laboratory, good operation, and various inspection conditions related to the study, sufficient number of subjects and familiarity of study participants with the requirements in the study protocol;
2. Monitor the investigator's implementation of the study protocol during the study, verify that informed consent of all subjects is obtained before the study, understand the enrollment rate of subjects and the progress of the study, and verify that the enrolled subjects are qualified;
3. Verify that the record and report of all data are correct and complete, all individual report forms are entered correctly, and that they are consistent with the original data. All errors and omissions should have been corrected or indicated, signed and dated by the investigator. The dose changes, treatment changes, combined medication, intermittent diseases, lost to follow-up, and test omissions of each subject should be verified and recorded. Verify that the withdrawal and lost to follow-up of enrolled subjects has been indicated in the individual report form.
4. Verify that all AEs are documented, and that SAEs are reported within the specified time and documented; verify that the investigational drug is supplied, stored, distributed and recovered according to relevant regulations and recorded accordingly.
5. Record the visit, study and examination that the investigator has failed to do, and whether to correct the mistakes and omissions clearly and truthfully;
6. Complete a written monitoring report after each visit, and the report should state the date and time of monitoring, name of monitor, monitoring findings, etc.

The quality assurance department of the sponsor may audit the study at the clinical testing facility. The audit contents include: drug supply, study documents required, record of informed consent process, and consistency between case report form and original documents. The audit contents and scope also can be added as needed. The investigator agrees to participate at appropriate time and in an appropriate manner.

# Statistical analysis

## Sample size

**Phase 2 Study**

Phase 2 is a single-arm study, with Simon two-stage design, in which Optimal method is used to calculate the sample size based on ORR (the primary endpoint) evaluated by the investigator. That is, in the case of ineffective treatment, the sample size should be minimized, so that as few subjects as possible are exposed to the ineffective treatment in Phase 2.

The α level is controlled at 0.025, the power is 80%. Assuming that the unacceptable response rate is ORR=40%, and the expected target response rate is ORR=60%, at least 16 subjects will be enrolled first. If ≥8 of the first 16 subjects who have obtained post-dose efficacy assessment have achieved response (CR/PR), enrollment will be further performed until 61 subjects, and if < 8 subjects have achieved response, the study will be terminated. After 61 subjects are enrolled, if ≥32 subjects achieve response, it is suggested that further development can be carried out; if < 32 subjects, the investigator and the sponsor will discuss whether to carry out follow-up research and development.

**Phase 3 Study**

Phase 3 is a randomized controlled study. Assuming that the median PFS of the control group is 10 months, the estimated hazard ratio (HR) of PFS (test group/control group) is 0.68, that is, the median PFS of the test group is 14.7 months. If the overall α level is controlled at one-sided 0.025, and the original hypothesis that the distribution of PFS is consistent between the two groups is tested by log-rank method, collecting 245 PFS events can provide a power of 85%. Assuming that the enrollment time is 12 months and the whole study duration is 26 months, and considering that 5% of the subjects drop every year, it is estimated that a total of 378 subjects are needed.

Collect the PFS data of subjects in Phase 2 before completion of enrollment in Phase 3. If the data in Phase 2 show that the median PFS in SHR-1701 combined with BP102 and XELOX group is equal to or higher than the preset median PFS in Phase 3, the sample size of number of events in Phase 3 remains unchanged. If the data in Phase 2 show that the median PFS in SHR-1701 combined with BP102 and XELOX group is lower than the preset median PFS in Phase 3, it is possible to update the efficacy hypothesis, increase the sample size of Phase 3 or the expected number of events to achieve sufficient power. Since the data results of Phase 3 are not used in adjustment of the sample size, α consumption is not involved.

It is planned to conduct an interim analysis when 70% PFS events (172 events) are collected in Phase 3, and the purpose of this interim analysis is to demonstrate that PFS in the test group is superior to that in the control group.

## Statistical analysis plan

The detailed statistical analysis of this study will be recorded in the statistical analysis plan (SAP), stored by the sponsor, and corrected before data unblinding. Appropriate modifications can be made in the SAP for plans identified in the protocol. However, any significant amendment to the definition and analysis of primary endpoints will need to be reflected in the protocol amendment.

## Statistical hypothesis

This study is designed to be a single-arm exploratory design, with does not involve hypothesis test.

The primary objective of Phase 3 of this study is to evaluate the PFS (evaluated by IRC) with SHR-1701 combined with BP102 and XELOX vs. placebo combined with BP102 and XELOX for the first-line treatment of patients with advanced colorectal cancer (evaluated by IRC), and the following hypotheses will be tested:

H_0_: The PFS in SHR-1701 combined with BP102 and XELOX group (test group) is ≤ that in placebo combined with BP102 and XELOX treatment group (control group)

H_1_: The PFS in SHR-1701 combined with BP102 and XELOX group (test group) is < that in placebo combined with BP102 and XELOX treatment group (control group)

In this study, Kaplan-Meier method will be used to estimate PFS curve, and stratified Log-rank method will be used as the main statistical method for hypothesis test (one-sided α=0.025).

In this study, an interim analysis will be conducted when about 70% PFS events are collected, and when the one-sided p value obtained by Log-rank test in interim analysis is less than or equal to 0.007 and the median PFS in the test group estimated by Kaplan-Meier method is greater than that in the control group, it can be determined that the efficacy in the test group is better than that in the control group. Alternatively, when the one-sided p value in the final analysis is less than 0.023 and the median PFS in the test group estimated by Kaplan-Meier method is greater than that in the control group, it suggests that the efficacy in the test group is better than that in the control group.

## Analysis population

**Phase 2 Study**

Full analysis set (FAS-S1): subjects who receive the investigational drug at least once after enrollment in Phase 2.

Evaluable set (ES-S1): a subset of FAS-S1, including subjects who are enrolled in Phase 2 and receive the investigational drug at least once, and receive at least tumor assessment after receiving the investigational drug;

ADA analysis set (ADAS-S1): All subjects enrolled in the Phase 2 study who have received the investigational medication at least once, with baseline and at least one post-baseline ADA evaluation data.

**Phase 3 Study**

Full analysis set (FAS-S2): subjects who receive the investigational drug at least once after randomization in Phase 3 according to the ITT principle.

Per protocol set (PPS-S2), A subset of FAS-S2 that excludes subjects with major protocol deviations that are judged to have a major impact on the results.

Safety set (SS-S2): subjects who receive the investigational drug at least once in Phase 3 (regardless whether they are randomized);

ADA analysis set (ADAS-S1): All subjects enrolled in the Phase 2 study who have received the investigational medication at least once, with baseline and at least one post-baseline ADA evaluation data.

## Statistical methods

### Basic method

Phase 2 of this study is designed to be a single-arm study, and Phase 3 is designed to be a parallel controlled study. Unless otherwise specified, all data will be analyzed by treatment stage and treatment group and using the corresponding statistics according to the data type: the measurement data are expressed as mean, standard deviation, median, minimum and maximum, the counting data are expressed as frequency and percentage, and for time-to-event data, the median survival time of each group will be estimated by Kaplan-Meier (KM) product limit method. The survival curve will be plotted and the 95% confidence interval of the median time will be estimated if necessary.

### Primary efficacy endpoint analysis

The efficacy analysis in Phase 2 will be conducted for the primary efficacy endpoint, the ORR evaluated by the investigator. The descriptive statistical analysis of ORR will be made based on the full analysis set (FAS-S1) of Phase 2, and the 95% confidence interval will be estimated using Wilson (Score) method.

The primary efficacy analysis in Phase 3 will be conducted for the primary efficacy endpoint, the PFS evaluated by IRC. Based on the Phase 3 full analysis set (FAS-S2), the end point of PFS will be tested by stratified log-rank test based on stratified factors to compare the inter-group difference. The median time and 12-month PFS rate will be calculated using Kaplan-Meier method, and a survival curve will be plotted. The Greenwood's method will be used to calculate the standard error, and the Brookmeyer-Crowley method based on log-log transformation will be used to estimate the two-sided 95% confidence interval for median PFS.

The supporting analysis of the primary endpoint is only for Phase 3:

- PFS will be evaluated based on the IRC of per-protocol set (PPS-S2) in Phase 3 for analysis, with analysis method the same as that of analysis of primary efficacy in Phase 3.
- In Phase 3, the stratified Cox proportional hazard model will be used to estimate the hazard ratio and 95% confidence interval of PFS in the test group compared with the control group. In addition, in order to explore the influence of other factors on efficacy, in addition to the treatment group, other factors may also be added to the model as covariates to be involved in the model fitting.

### Secondary efficacy endpoint analysis

The secondary efficacy analysis in Phase 2 in this study will be based on the full analysis set (FAS-S2) in Phase 2, and will be aimed at the DCR, PFS, DOR and OS evaluated by the investigator.

- In Phase 2, the median time and 12-month survival rate will be calculated using Kaplan-Meier method, and a survival curve will be plotted;
- For the PFS evaluated by the investigator in Phase 2, the median time and 12-month PFS rate will be calculated using Kaplan-Meier method, and a survival curve will be plotted;
- The DoR evaluated by the investigator in Phase 2 will be tested using the same method as PFS for subjects with response after treatment. The censoring rules for DoR are the same as that for PFS, and the date of end of response must be the same as the disease progression or death date of PFS; descriptive statistical analysis will be performed for the ORR and DCR evaluated by the investigator in Phase 3, and the 95% confidence interval will be estimated by Wilson (Score) method.

The secondary efficacy analysis in Phase 3 will be based on the full analysis set (FAS-S2) in Phase 3, and will be aimed at the PFS, DOR, ORR, DCR and OS evaluated by the investigator.

- For the PFS evaluated by the investigator in Phase 3, the differences between groups will be compared by the same stratified Log-rank method as the PFS analysis method evaluated by IRC in Phase 3, and HR will be estimated based on Cox proportional hazard model.
- In Phase 3, OS will be analyzed using the same method as the PFS analysis method evaluated by IRC in Phase 3.
- The analysis of DOR evaluated by the investigator in Phase 3 is limited to the subjects who achieve response, and the same analysis method is used as the PFS in Phase 3 evaluated by IRC. The censoring rules for DoR are the same as that for PFS, and the date of end of response must be the same as the disease progression or death date of PFS.
- Descriptive statistical analysis will be performed for the ORR evaluated by the investigator in Phase 3, and the 95% confidence interval of single group will be estimated by Wilson (Score) method. 95% CI of the rate difference between the two groups will be estimated by Newcombe method.
- Descriptive statistical analysis will be performed for the DCR evaluated by the investigator in Phase 3, and the 95% confidence interval of single group will be estimated by Wilson (Score) method. 95% CI of the rate difference between the two groups will be estimated by Newcombe method.

### Safety analysis

Safety analysis will be based on Phase 2 FAS-S1 and Phase 3 safety set (SS-S2) (actual medication group). Safety analysis is limited to descriptive statistical summary, including but not limited to the following aspects:

- Summary of treatment-emergent adverse events (all-cause and treatment-related);
- Incidence and severity of treatment-emergent adverse events (all-cause and treatment-related);
- Summary of details of treatment-emergent serious adverse events;
- Analysis of correlation of treatment-emergent adverse events;
- Incidence of dose suspension, dose reduction and dose termination due to drug-related toxicity during the study;
- Descriptive analysis of laboratory indicators, vital signs and ECG data;

In this study, AEs will be coded using Medical Dictionary for Regulatory Activities (MedDRA). In this study, all treatment-emergent AE, drug-related AEs, SAEs and drug-related SAEs will be tabulated according to the NCI CT CAE version 5.0 criteria and the worst grade. The laboratory test parameters during the study period will be summarized by using the worst grade according to the NCI CTCAE version 5.0 criteria.

### Interim analysis

In the Phase 3 randomized controlled study, an interim analysis is planned for the primary efficacy endpoint, the progression free survival (PFS) evaluated by IRC, and it is planned to conduct this analysis when about 172 events are collected. The objectives of the interim analysis include:

1. Terminate the study early because of superior effect;
2. Continue the study as scheduled;

Judgment rule for study termination at interim analysis due to superior effect: determine the statistically significant analysis boundary according to Lan-DeMets α consumption function and O'Brien-Fleming boundary. During the interim analysis, 172 PFS events occur (estimated to be about 17 months after enrollment of the first patient), accounting for 70% of the total estimated 245 events, with α consumption of about 0.007. The superiority margin is that when the single-sided *p* value is less than 0.007, it suggests that the efficacy in the test group is better than that in the control group. The α consumption can be adjusted according to the actual number of PFS events in the interim analysis. If the interim analysis does not terminate the study due to superior effect, the final analysis will be performed when about 245 PFS events are collected (estimated to be 26 months after enrollment of the first patient). According to Lan-DeMets α consumption function, when the single-sided *p* value is less than 0.023, it suggests that the efficacy in the test group is superior to that in the control group.

### Subgroup analysis

The primary efficacy endpoint, the PFS evaluated by IRC in Phase 3 will be subject to subgroup analysis according to the following factors (including but not limited to):

PD-L1 expression (<1% or ≥1%);

Position of tumor primary lesion (left and right)

RAS gene status (mutant type and wild type).

### Multiple comparisons/multiplicity

In Phase 3, an interim analysis will be performed when 70% PFS events are collected, and the multiplicity will be controlled using Lan-DeMets α consumption function and O’Brien-Fleming margin.

### Exploratory analysis

The immunogenicity of SHR-1701 during treatment will be evaluated by comparing the baseline conditions of subjects, including the positive rate of anti-SHR-1701 antibody (ADA), proportion of neutralizing activity (NAb) of anti-SHR-1701 antibody, and the correlation between the immunogenicity of SHR-1701 and the trough concentration (C_trough_), safety and efficacy of SHR-1701 will be summarized and analyzed.

# Data monitoring committee

## Data monitoring committee (DMC)

In this study, an independent data monitoring committee (IDMC) will be established to assess the safety and efficacy data. The efficacy and safety will be evaluated during the mid-term analysis in the Phase 3 study, and the subsequent study will be suggested according to the results of the interim analysis to protect the safety of the subjects and meet the ethical requirements for further study.

The DMC will be composed of clinical experts and statistical experts who are not employed by Hengrui and have no major conflicts of interest. The review meeting will be held according to the DMC charter and the study will be continued during the meeting. After data review, DMC will provide the suggestions for early termination, further study or protocol amendment, and whether the suggestions should be adopted will be decided by Jiangsu Hengrui Pharmaceutical Co., Ltd.

For details, refer to the charter of the independent data monitoring committee.

# Data management method

The sponsor is responsible for the clinical trial data management of this study, and uses an electronic data capture (EDC) system to collect and manage the study data.

## Data collection

### Completion of Electronic Case Report Form (eCRF)

The eCRF is completed by the investigator or data entry personnel via the EDC system. The ECRF should be completed in a timely manner and ensure that the completed data are traceable from the original records. When data modification is performed in EDC, it is required to fill in the reason for data modification according to the system prompts. The modification history and reasons for modification will be documented in the audit trail of the EDC system. The investigator or authorized personnel needs to confirm the authenticity, completeness, and timeliness of the eCRF data and electronically sign in the EDC system.

### Use of Electronic Data Capture (EDC) system

The data manager builds the eCRF and logic verification program in the EDC system according to the study protocol, and completes the user acceptance test and go-live use before the first subject is enrolled. All EDC users are required to complete the relevant training and archive the training records in order to gain access to the study eCRF. When the user implements an electronic signature on the eCRF, the statement of use of the electronic signature needs to be confirmed and agreed. The account is limited to the user's own use, and the password needs to be kept properly and changed regularly. When there is a change of personnel in the study team, the authority needs to be canceled in time.

## Data Management

### ECRF data review

The logic verification program of EDC system will check the integrity and logic of the entered data and raise queries for data that may have problems. The investigator or data entry personnel may correct the data or interpret and confirm it by answering queries. The monitors, the data manager, and the medical reviewer will also review the eCRF data and raise queries for questionable data if necessary. The investigator should answer queries from the system and the data reviewer in a timely manner, and may raise queries several times until the data issue is resolved if necessary.

### Data review meeting and database lock

Prior to database lock, the study team needs to complete data cleaning, summarize all protocol deviation events that occur during the study, and hold a data review meeting to determine the analysis population. Decisions made during the data review meeting will be documented. After the data review meeting is approved, the study team confirms to lock the study database in the EDC system, and no changes can be made to the data after locking.

### Data archiving

After the study is completed, the EDC system needs to generate the eCRF of subjects in PDF format, which will be stored on CD-ROMs and archived by the sponsor and each institution for audit. The study data should be stored and managed according to GCP requirements, and the investigator should inform the sponsor in advance when destroying any documents or records related to the study. The sponsor should keep the clinical study data for at least 15 years after the investigational drug is approved for marketing, or even if the clinical study is terminated early.

# Original Data and Original documents

In accordance with ICH E6, relevant regulations, and the research institution's requirements for the protection of the subject's personal information, each site must properly maintain records related to this study. As part of Hengrui's sponsorship or participation in the study, each site shall allow authorized representatives of Hengrui and the regulatory authority to inspect (and, if permitted by law, copy) clinical records for quality review, audit, and evaluation of safety, study progress, and data validity.

Original data are all information necessary for reconstruction and evaluation of the clinical study and are the original records of clinical findings, observations, or other activities. Examples of these original documents and data records include, but are not limited to: hospital records, laboratory records, memos, subject’s diary card, pharmacy dispensing records, recordings of consultation meetings, recorded data from automated instruments, copies or transcriptions verified to be accurate and complete, microfiches, photographic negatives, microfilm or disks, radiographs, and documents and records of subjects kept in participating pharmacies, laboratories, and medico-technical departments.

# Quality Assurance and Quality Control

In order to ensure the quality of the study, the sponsor and the investigator will jointly discuss and develop the clinical study plan before the study officially begins, and will confirm whether the relevant study personnel participating in the study have received appropriate GCP training.

Study medications must be managed by each site in accordance with SOPs, including receipt, storage, dispensing, recovery, and destruction.

According to GCP guidelines, necessary steps should be taken during the design and conduct of the study to ensure that the data collected are accurate, consistent, complete and reliable. All observed results and abnormal findings in the clinical study should be timely verified and recorded to ensure the reliability of data. The instruments, equipment, reagents and standards used for various inspection items in the clinical study should have strict quality standards and ensure that they can work in normal state.

The investigator enters the information required by the protocol into the eCRF, and the monitor verifies whether it is completed completely and accurately, and guides the staff of the site to make necessary corrections and additions.

The drug regulatory authority, the ethics committee, the monitor and/or the auditor of the sponsor may conduct a systematic inspection on study-related activities and documents to evaluate whether the study is conducted in accordance with the study protocol, SOPs, and relevant regulatory requirements, and whether the study data are recorded in a timely, true, accurate, and complete manner. Audits should be performed by personnel not directly involved in the clinical study.

# Ethics

## Ethical Norms

This clinical study must comply with the Council for International Organizations of Medical Sciences (CIOMS, 2002), current ICH-GCP, the Declaration of Helsinki, and applicable regulations. The study protocol, protocol amendment, informed consent form and other relevant documents, such as recruitment advertisement, should be provided to the ethics committee before the start of the study. This study can only be conducted after being approved by relevant regulatory authorities and hospital ethics committee in advance.

Without the consent of both the sponsor and the investigator, neither party shall unilaterally modify the protocol of this study. The revised protocol may not be implemented until approved by the ethics committee. If the investigator has to enter the protocol deviation process in order to eliminate apparent direct hazards to the subjects, the investigator must inform the ethics committee and the sponsor in writing immediately after the deviation has been implemented, and explain and record all protocol deviations made.

During the clinical study, any modification to this study protocol should be submitted to the ethics committee, and other study documents should be modified accordingly if necessary, and submitted and/or reviewed in accordance with the requirements of the ethics committee. The investigator should be responsible for submitting the interim report regularly in accordance with the relevant requirements of the ethics committee, and should notify the ethics committee that the study has ended after the end of the study.

## Independent ethics committee

The protocol, informed consent form, recruitment materials and materials of all subjects will be submitted to the ethics committee for review and approval. Subjects may not be enrolled until the protocol and the informed consent form are approved. Any amendment to the protocol must be reviewed and approved by the ethics committee before implementation. All amendments to the informed consent form must also be approved by the ethics committee, and the ethics committee will decide whether the new version needs to be signed again for subjects who have signed the previous version of the informed consent form.

## Informed Consent

### Informed consent form and other written information required by subjects

The informed consent form describes the study medication and study process in detail, and fully explains the risks of the study to the subjects. Written documentation of informed consent must be obtained before the subjects perform any study-related procedures.

The informed consent form must meet the requirements of ICH GCP and local laws and regulations, including applicable privacy laws.

### Informed consent process and records

Informed consent begins before the subjects agree to participate in the clinical study and continues throughout the clinical study. The investigator will discuss the risks and possible benefits of participating in the study in detail and fully with the subject or his/her legal representative. Subjects will be required to review the informed consent form approved by the ethics committee. The investigator will explain the clinical study to the subject and answer any questions that the subject may raise. Subjects may not start participating in the study until informed consent is obtained. Subjects can withdraw consent at any time during the course of the clinical study. A copy of the informed consent form will be retained by the subject. Even if the patient consulted refuses to participate in the study, his/her rights and interests will be fully protected and the quality of his/her medical care will not be affected in any way.

## Confidentiality of Subject Information

Confidentiality of subject information shall be strictly enforced by the investigator, personnel participating in the study, the sponsor and its representative. Confidentiality also covers biological samples and genetic testing in addition to the clinical information of subjects. Therefore, the study protocol, documents, data and all other information generated therefrom will be kept strictly confidential. All relevant study or data information may not be disclosed to any unauthorized third party without the prior written approval from the sponsor.

Other authorized representatives of the sponsor, the ethics committee, and regulatory authorities can inspect all documents and records required to be maintained by the investigator, including, but not limited to, medical records and medication records of subjects. The site should allow access to these records.

Subject contact information will be kept securely at each site and will be used only internally during the study. At the end of the study, all records will continue to be kept in a safe place according to the timelines specified by the local ethics committee and regulations.

For the statistical analysis and scientific report of subjects’ study data collected, this should not include the subject's contact or identifying information. Instead, individual subjects and their study data will have a separate study identification number. The study data entry and study management systems used by study personnel at each clinical study site are kept confidential and password-protected. At the end of the study, all identifying information from the study database will be eliminated and archived at each clinical study site.

The sponsor will follow applicable privacy laws, and the subjects’ personal data will be kept confidential at a high standard.

## Future Use of Preserved Specimens

This study will not use the preserved specimens for purposes other than those specified in the protocol.

# Publication of Study Results

The study results remain the property of Hengrui. The investigator who plans to publish any study related data and information should get consent of Hengrui, and should provide Hengrui with the original manuscript, abstract or full text of the planned publication (posters, invited presentations or guest lectures) at least 30 days prior to submission for publication or other form of publication.

# Finance and Insurance

The sponsor will purchase commercial insurance for all subjects.

# Reference

1. Zheng Rongshou, Sun Kexin, Zhang Siwei, et al., 2015, Epidemiological Analysis of Malignant Rumors in China [J]. Chinese Journal of Oncology, 2019, 41(1):19-28.
2. Wang Xishan, Standardized Implementation of Comprehensive Treatment of Colorectal Cancer [J]. Chinese Journal of Practical Surgery, 2010 (4):260-264.
3. Wen Lei, Zhang Hongmei, Xu Li, Application of Bevacizumab in Resisting Colorectal Cancer [J]. China Cancer, 2016(7):534-541.
4. Karuna, Ganesh, Zsofia, et al. Immunotherapy in colorectal cancer: rationale, chall-enges and potential [J]. Nature Reviews Gastroenterology & Hepatology, 2019.
5. Overman M J , Lonardi S , Wong K Y M , et al. Durable clinical benefit with n-ivolumab plus ipilimumab in DNA mismatch repair-deficient/microsatellite instability-high metastatic colorectal cancer[J]. Journal of Clinical Oncology Official Journal of the American Society of Clinical Oncology, 2018, 36(8):773.
6. Shahda S, Noonan A M, Bekaii-Saab T S, et al. A phase II study of pembrolizu-mab in combination with mFOLFOX6 for patients with advanced colorectal cancer[J]. 2017.
7. Ghiringhelli F, Chibaudel B, Taieb J, et al. Durvalumab and tremelimumab in combination with FOLFOX in patients with RAS-mutated, microsatellite-stable, previously untreated metastatic colorectal cancer (MCRC): Results of the first intermediate analysis of the phase Ib/II MEDETREME trial[J]. 2020.
8. Hu Mengxue, Xu Bin, Yu Jinming, et al., Study Progress in PD-L1/TGF-β Dual-function Inhibitor Fusion Protein M7824 [J]. International Journal of Oncology, 2019, 46(5):281-284.
9. Kopetz S, Spira A I, Wertheim M, et al. M7824 (MSB0011359C), a bifunctional fusion protein targeting PD-L1 and TGF-β, in patients with heavily pretreated CRC: Preliminary results from a phase I trial[J]. 2018.
10. Zhang Baihong, Yue Hongyun, New Tumor Treatment Pattern: Chemotherapy Combined with Immunotherapy [J]. Journal of Modern Oncology, 2018, v.26; No.250(16):165-167.
11. Fukumura D, Kloepper J, Amoozgar Z, Duda DG, Jain RK: Enhancing cancer immunotherapy using antiangiogenics: opportunities and challenges[J]. *Nature reviews Clinical oncology* 2018, 15(5):325-340.

Attachment 1 Response Evaluation Criteria in Solid Tumors version 1.1 (RECIST v1.1)

1. **Measurability of tumor at baseline**
   1. **Definitions**

At baseline, tumor lesions/lymph nodes will be categorized measurable or non-measurable as follows:

**Measurable lesions**

Tumor lesions: must be accurately measured in at least one dimension (longest diameter in the plane of measurement is to be recorded) with a minimum size of:

- CT scanning 10 mm (CT scanning slice thickness no more than 5 mm)
- 10 mm caliper measurement by clinical exam (lesions which cannot be accurately measured with calipers should be recorded as non-measurable)
- Chest X-ray 20 mm
- Malignant lymph nodes: To be considered pathologically enlarged and measurable, a lymph node must be ≥ 15mm in short axis when evaluated by CT scan (CT scan slice thickness recommended to be no greater than 5 mm). At baseline and follow-up, only the short axis will be measured and followed.

**Unmeasurable lesions**

All other lesions, including small lesions (longest diameter < 10 mm or pathological lymph node with ≥ 10 mm to < 15 mm short axis) and non-measurable lesions. Lesions considered truly non-measurable include: leptomeningeal disease, ascites, pleural or pericardial effusion, inflammatory breast disease, lymphangitis carcinomatosa of the skin/lung, abdominal mass not confirmed and followed by imaging, and cystic lesions.

**Special considerations regarding lesion measurement**

Bone lesions, cystic lesions and lesions previously treated with local therapy should be particularly noted:

Bone lesions:

- Bone scans, PET scans, or photographs are not suitable for measuring bone lesions, but can be used to confirm the presence or absence of bone lesions;
- Lytic lesions or mixed lytic/osteoblastic lesions, with identifiable soft tissue components, that can be evaluated by cross-sectional imaging techniques such as CT or MRI can be considered as measurable lesions if the soft tissue component meets the definition of measurability described above;
- Blastic bone lesions are non-measurable.
- Cystic lesions:
- A lesion that meets the definition of a simple cyst in radiography should not be considered a malignant lesion because it is a simple cyst in the definition, and it is neither a measurable lesion nor an unmeasurable lesion;
- If it is a cystic metastatic lesion and meets the above definition of measurability, it can be treated as a measurable lesion. However, if there are non-cystic lesions in the same patient, non-cystic lesions should be preferred as target lesions.

Locally treated lesions;

- Lesions situated in a previously irradiated area, or in an area subjected to other topical therapy, are usually considered non-measurable unless it has demonstrated clear progression in the lesion. The study protocol should detail the conditions under which such lesions can be considered measurable.
  1. **Description of measurement method**

**Lesion measurements**

During clinical evaluation, all tumor measurements shall be recorded in metric system. All baseline assessments of tumor lesion size should be completed before the start of treatment, and must be completed within 28 days (4 weeks) before the start of treatment.

**Evaluation method**

The same techniques and methods should be used for baseline assessment and subsequent measurement of lesions. Except for lesions that cannot be evaluated by imaging but can only be evaluated by clinical examination, all lesions must be evaluated by imaging.

Clinical lesions: Clinical lesions will only be considered measurable when they are superficial and ≥ 10mm in diameter as evaluated using calipers (e.g., skin nodules). For patients with skin lesions, documentation by color photography including a ruler to estimate the size of the lesion is suggested. When lesions can be evaluated by both clinical exam and imaging, imaging evaluation should be undertaken since it is more objective and may also be reviewed at the end of the study.

Chest X-ray: Chest CT is preferred over chest X-ray, particularly when progression is an important endpoint, since CT is more sensitive than X-ray, particularly in identifying new lesions. Chest X-ray detection is only applicable when the boundary of the measured lesion is clear and the lungs are well ventilated.

CT, MRI: CT is the best currently available and reproducible method to measure lesions selected for response assessment. This guideline has defined measurability of lesions on CT scan based on the assumption that CT slice thickness is 5mm or less. When CT scans have slice thickness greater than 5 mm, the minimum size for a measurable lesion should be twice the slice thickness. MRI is also acceptable in certain situations (e.g. for body scans).

Ultrasound: Ultrasound should not be used as a method to measure lesion size. Ultrasound inspection is not repeatable after the measurement due to its operational dependence, and cannot guarantee the sameness of technique and measurement between different measurements. If new lesions are identified by ultrasound in the course of the study, confirmation by CT or MRI is advised. If there is concern about radiation exposure at CT, MRI may be used instead of CT.

Endoscopy, laparoscopy: The utilization of these techniques for objective tumor evaluation is not advised. However, they can be useful to confirm CR when biopsies are obtained or to determine relapse in trials where relapse following CR or surgical resection is an endpoint.

Tumor markers: Tumor markers alone cannot be used to assess objective tumor response. However, if markers are present at baseline above the upper limit of normal, they must be normalized for a subject to be considered in complete response. Because tumor markers are disease specific, instructions for their measurement should be incorporated into protocols on a disease specific basis. Specific criteria for both CA-125 response (in recurrent ovarian cancer) and PSA response (in recurrent prostate cancer) have been published. In addition, the Gynecologic Cancer Intergroup has developed CA125 progression criteria which are to be integrated with objective tumor assessment for use in first-line trials in ovarian cancer.

Cytological/histological techniques: These techniques can be used to differentiate between PR and CR in certain situations if required by protocol (e.g. residual benign tumor tissue in lesions of germ cell tumors). When effusions are known to be a potential adverse effect of treatment (e.g. with certain taxane compounds or angiogenesis inhibitors), the cytological confirmation of the neoplastic origin of any effusion that appears or worsens during treatment can be considered if the measurable tumor has met criteria for response or stable disease in order to differentiate between response (or stable disease) and progressive disease.

1. **Assessment of tumor response**
   1. **Target lesion assessment**

Complete Response (CR): Disappearance of all target lesions. All pathological lymph nodes (including target and non-target nodules) must have reduction in short axis to < 10 mm.

Partial Response (PR): The sum of the diameters of the target lesions is reduced by at least 30% from the baseline level.

Progressive Disease (PD): Taking the minimum value of the sum of the diameters of all target lesions measured on study as the reference, at least a 20% increase in the sum of the diameters of target lesions (if the baseline measurement value is the smallest, use the baseline value as the reference); in addition, the sum must also demonstrate an absolute increase of at least 5 mm (the appearance of one or more new lesions is also regarded as disease progression).

Stable Disease (SD): Neither sufficient shrinkage to qualify for PR nor sufficient increase to qualify for PD, taking as reference the smallest sum diameters while on study.

- 1. **Special notes on target lesion assessment**

Lymph nodes: Even if the lymph nodes identified as target lesions are reduced to less than 10 mm, the actual short axis value corresponding to the baseline must be recorded for each measurement (consistent with the anatomical plane of the baseline measurement). This means that when lymph nodes are included as target lesions, the 'sum' of lesions may not be zero even if complete response criteria are met, since a normal lymph node is defined as having a short axis of < 10 mm. Electronic case report forms or other data collection methods may therefore be designed to have target nodal lesions recorded in a separate section where, in order to qualify for CR, each node must achieve a short axis < 10 mm.; for PR, SD and PD, the actual short axis measurement of the nodes is to be included in the sum of target lesions.

Target lesions that are too small to measure: While on study, all lesions (nodal and non-nodal) recorded at baseline should have their actual measurements recorded at each subsequent evaluation, even when very small (e.g. 2 mm). However, sometimes lesions or lymph nodes which are recorded as target lesions at baseline become so faint on CT scan that the radiologist may feel difficult to assign an exact measure and may report them as being 'too small to measure'. When this occurs, it is important to record a value on the eCRF. If it is the opinion of the radiologist that the lesion has likely disappeared, the measurement should also be recorded as 0 mm. If the lesion does exist but is rather vague, and an accurate measurement value cannot be given, the default value is 5 mm. (Note: Lymph nodes are unlikely to have this condition, because they generally have a measurable size under normal conditions, or are often surrounded by fatty tissue as they are in the retroperitoneal cavity; however, if this kind of measurement value cannot be given, the default value is 5 mm). The default value of 5 mm is derived from the cutting thickness of the CT scan (this value does not change due to different cutting thickness values of CT). Since there is little chance that the same measurement value will recur, providing this default value will reduce the risk of erroneous evaluation. To reiterate, however, if the radiologist is able to provide an exact measure of lesion size, the actual measure must be recorded, even if the lesion is smaller than 5 mm in diameter.

Separate or combined lesions: When non-nodular lesions are divided into fragments, add the longest diameters of the separated parts to calculate the sum of the diameters of the lesions. Similarly, for combined lesions, the planes between the combined parts can be distinguished, and then the maximum diameter of each can be calculated. However, if the combination is inseparable, the longest diameter should be the longest diameter of the entire fusion lesion.

- 1. **Assessment of non-Target lesions**

This section defines the criteria for tumor response in non-target lesions. While some non-target lesions may actually be measurable, they need not be measured and only qualitatively evaluated at the time points specified in the protocol.

Complete Response (CR): Disappearance of all non-target lesions and normalization of tumor marker level. All lymph nodes must be non-pathological in size (short axis < 10 mm).

Non-CR/Non-PD: Presence of one or more non-target lesion (s) and/or maintenance of tumor marker level above the normal limits.

Progressive disease: Unequivocal progression of existing non-target lesions. Note: The appearance of one or more new lesions is also considered progression.

- 1. **Special notes on assessment of progression of non-target disease**

The definition of progression of non-target disease requires additional explanation as follows: When the patient also has measurable disease, to achieve 'unequivocal progression' on the basis of the non-target disease, there must be an overall level of substantial worsening in non-target disease such that, even if in presence of SD or PR in target disease, the overall tumor burden for non-target lesion has increased sufficiently to discontinue the therapy. A modest 'increase' in the size of one or more non-target lesions is usually not sufficient to qualify for progression; therefore, it may be rare for changes in non-target lesions alone to define overall tumor progression in the face of SD or PR of target lesions.

When patients have non-measurable non-target disease: This circumstance arises in some Phase 3 trials when it is not a criterion of study entry to have measurable disease. The overall assessment still refers to the above criteria, but because there is no measurable data of the lesion in this case. The deterioration of non-target lesions is not easy to evaluate (by definition: all non-target lesions must be truly unmeasurable). Therefore, when changes in non-target lesions lead to an increase in the overall disease burden that is equivalent to disease progression in the target lesions, a clear definition of progression based on non-target lesions requires the establishment of an effective detection method for evaluation. As described, an increase in tumor burden is equivalent to an additional 73% increase in volume (equivalent to a 20% increase in the diameter of a measurable lesion). Examples include an increase in a pleural effusion from 'trace' to 'large', an increase in lymphangitic disease from 'topical' to 'widespread', or may be described in protocols as 'sufficient to require a change in therapy'. Examples include pleural effusions ranging from trace to large, lymphatic involvement spreading from the primary site to distant, or may be described in the protocol as 'necessitating a change in therapy'. If clear progression is found, the patient should be regarded as disease progression overall at that point in time. While it is preferable to have objective criteria to be applied in the assessment of non-measurable disease, increased criteria must be reliable.

- 1. **New lesions**

The appearance of new malignant lesions denotes disease progression; therefore, some comments on new lesions are important. There are currently no specific standards for imaging detection of lesions, but the discovery of a new lesion should be clear. I.e. not attributable to differences in scanning technique, change in imaging modality or findings thought to represent something other than tumor (for example, some 'new' bone lesions may be simply healing or flare of pre-existing lesions). This is important when the subject's baseline lesions show partial or complete response. For example, necrosis of a liver lesion may be reported on a CT scan report as a new cystic lesion, which it is not.

The lesions that have been detected during follow-up but not found in the baseline examination will be regarded as new lesions and indicate disease progression. An example of this is the patient who has visceral disease at baseline and while on study has a CT or MRI brain scan which reveals metastases. The patient's brain metastases are considered to be evidence of PD even if he/she did not have brain imaging at baseline.

If a new lesion is equivocal, for example because of its small size, continued therapy and follow-up evaluation will clarify if it represents truly new disease. If repeat scans confirm there is definitely a new lesion, then time for progression should be the date of the initial scan.

While FDG-PET response assessments need additional testing for confirmation, it is sometimes reasonable to incorporate the use of FDG-PET scanning and complement CT scanning in assessment of progression (particularly possible new disease). New lesions via FDG-PET can be identified according to the following procedure:

A negative FDG-PET at baseline and a positive FDG-PET at follow-up is a sign of PD.

No FDG-PET at baseline and a positive FDG-PET result at follow-up:

If the positive FDG-PET at follow-up corresponds to a new site of disease confirmed by CT, this is PD.

If the positive FDG-PET at follow-up is not confirmed as a new site of disease on CT, additional CT scans are needed for confirmation (if so, the date of PD will be the date of the initial abnormal FDG-PET scan).

If the positive result of the follow-up FDG-PET examination is consistent with the existing lesion by CT examination, and the lesion does not progress on imaging tests, then the disease has not progressed.

- 1. **Missing assessments and non-evaluable designation**

If no imaging/measurement can be done at a particular time point, the patient is not evaluable (NE) at that time point. If only partial lesion measurements are made at an assessment, usually the case is also considered NE at that time point, unless a convincing argument can be made that the contribution of the individual missing lesion (s) would not change the assigned time point response.

- 1. **Special notes on response assessment**

When nodal lesions are included in the sum of target lesions, and the nodes decrease to 'normal' size (< 10 mm), they may still have a lesion measurement scan report. To avoid overestimation the situation on the increase in the size of the nodule, the measurement result should be recorded even if the nodule is normal. As mentioned earlier, this means that subjects with CR will not have a total sum of 'zero' in the eCRF.

If efficacy confirmation is required during the trial, repeated 'unmeasurable' time points will complicate the best efficacy evaluation. The analysis plan of the trial must state that these missing data/assessments can be explained clearly when determining efficacy. For example, in most trials, the response of a subject's PR-NE-PR can be regarded as a confirmation of efficacy.

That subjects with a systematic deterioration of health status require discontinuance of treatment without objective evidence of disease progression should be reported as symptomatic progression. Every effort should be made to assess objective progression even after treatment discontinuance. Assessment description on symptomatic deterioration is not an objective response, and it is the reason for stopping treatment. The objective response status of such subjects will be determined by evaluation of target and non-target lesions as shown in Attached Tables 1-3.

Cases that are defined as early progression, early death and non-evaluable conditions are special cases of the study and should be clearly described in each protocol (depending on the treatment interval and treatment period).

In some cases, it is difficult to distinguish local lesions from normal tissues. When the evaluation of complete response is conducted under this situation, we recommend that a biopsy be performed before the evaluation of the efficacy of complete response in local lesions. When the abnormal imaging test results of some subjects' focal lesions are considered to represent fibrosis or scar formation, FDG-PET is used as an evaluation standard similar to biopsy to confirm the efficacy of complete response. In this case, the application of FDG-PET should be described prospectively in the plan, and the report of the specialist medical literature for this situation should be used as support. However, it must be acknowledged that both approaches may lead to false positive CR due to limitations of FDG-PET and biopsy resolution/sensitivity.

Attached Table 1 Time Point Response - Subjects with Target (+/- Non-target) Lesions

| **Target lesions** | **Non-target lesions** | **New lesions** | **Overall response** |
| --- | --- | --- | --- |
| CR | CR | None. | CR |
| CR | Non-CR/non-PD | None. | PR |
| CR | Not evaluable | None. | PR |
| PR | Non-progressive or not fully evaluated | None. | PR |
| SD | Non-progressive or not fully evaluated | None. | SD |
| Not fully evaluated | Non-progressive | None. | NE |
| PD  Any condition  Any condition | Any condition  PD  Any condition | Yes or No  Yes or No  Yes | PD  PD  PD |

Note: CR = complete response, PR = partial response, SD = stable disease, PD = progressive disease, NE = not evaluable.

Attached Table 2 Time Point Response - Subjects with Non-target Disease only

| **Non-target lesions** | **New lesions** | **Overall response** |
| --- | --- | --- |
| CR | None. | CR |
| Non-CR or Non-PD | None. | Non-CR or Non-PD |
| Not fully evaluated | None. | Not evaluable |
| Unequivocal PD | Yes or No | PD |
| Any condition | Yes | PD |

Note: 'Non-CR/non-PD' is superior to 'stable disease' for non-target lesions. Since SD is increasingly used as endpoint for assessment of efficacy in some trials, this description is to address the situation where there is no provision for non-measurable lesions.

For equivocal findings of progression (e.g., very small and uncertain new lesions; cystic changes or necrosis in existing lesions), treatment may continue until the next scheduled assessment. If at the next scheduled assessment, progression is confirmed, the date of progression should be the earlier date when progression was suspected.

Attached Table 3 Best Overall Response when Confirmation of CR and PR is Required

| **Overall response at first time point** | **Overall response at subsequent time point** | **Best overall response** |
| --- | --- | --- |
| CR | CR | CR |
| CR | PR | SD, PD or PRa |
| CR | SD | SD provided that the minimum criteria for SD duration is met, otherwise PD |
| CR | PD | SD provided that the minimum criteria for SD duration is met, otherwise PD |
| CR | NE | SD provided that the minimum criteria for SD duration is met, otherwise NE |
| PR | CR | PR |
| PR | PR | PR |
| PR | SD | SD |
| PR | PD | SD provided that the minimum criteria for SD duration is met, otherwise PD |
| PR | NE | SD provided that the minimum criteria for SD duration is met, otherwise NE |
| NE | NE | NE |

Note: CR means complete response, PR means partial response, SD means stable disease, PD means progressive disease, and NE means not evaluable. Superscript "a": If a CR is truly met at first time point, then any disease seen at a subsequent time point, even disease meeting PR criteria relative to baseline, will have a efficacy evaluation as PD at that point (since the disease have reappeared after CR). The best response depends on whether SD occurs at the shortest treatment interval. However, sometimes 'CR' may be claimed first while subsequent scans suggest small lesions are likely still present. Hence, in fact the subject have PR, not CR at the first time point. In this case, the original CR should be changed to PR and the best response is PR.

- 1. **Confirmation of response assessment/duration of response**

**Confirmation of efficacy**

For non-randomized clinical studies with tumor response efficacy as the primary study endpoint, the efficacy of PR and CR must be confirmed to ensure that the efficacy is not the result of evaluation errors. In studies where stable disease or disease progression are the primary endpoints, confirmation of response is not required since it will not add value to the interpretation of trial results. In the case of SD, within the shortest time interval after the start of the trial (generally no less than 6 to 8 weeks), at least one measurement meets the SD standard specified in the protocol.

**Duration of overall response**

The duration of overall response is measured from the time when measurement criteria for CR/PR are first met (whichever is first recorded) to the first date when relapse or progressive disease is objectively documented (taking as reference the smallest measurements for progressive disease recorded on study). The duration of overall complete response is measured from the time when measurement criteria for CR are first met to the first date when relapse or progressive disease is objectively documented.

**Stable disease**

Stable disease is measured from the start of the treatment (in randomized trials, from date of randomization) to the time when the criteria for progression are met, taking as reference the smallest sum on study (if the baseline sum is the smallest, this is the reference for calculation of PD). The clinical relevance of stable disease varies with different studies and different diseases. If the proportion of subjects achieving stable disease for a minimum period of time is an endpoint of importance in a particular trial, the protocol should specify the minimum time interval required between two measurements for determination of SD.

Note: The duration of response and stable disease as well as PFS are influenced by the frequency of follow-up after baseline evaluation. It is not within the scope of this guideline to define a standard follow-up frequency. The frequency of follow-up should take into account many factors, such as disease type and stage, treatment cycle and standard practice. However, these limitations in the accuracy of the measured endpoints should be taken into account if comparisons between trials are needed.

- 1. **PFS/TTP**

Many studies on advanced cancer have used PFS or TTP as the primary endpoint. If the protocol requires that all patients have measurable disease, evaluation of progression is relatively straightforward. An increasing number of studies allow patients with and without measurable disease to enter the study. In such cases, the clinical findings of disease progression in patients without measurable disease must be clearly described in detail. Because there is often an established bias in the date of progression, the timing of observations should be the same for each test group.

Attachment 2 Performance Status Scoring Criteria (ECOG)

(Eastern Cooperative Oncology Group)

| **ECOG score;** | **Criteria** |
| --- | --- |
| 0 | Fully active, able to carry on all pre-disease performance without restriction |
| 1 | Restricted in physically strenuous activity but ambulatory and able to carry out work of a light or sedentary nature |
| 2 | Ambulatory and capable of all self-care but unable to carry out any work activities. Up and about more than 50% of waking hours |
| 3 | Capable of only limited self-care, confined to bed or chair more than 50% of waking hours |
| 4 | Completely disabled. Cannot carry on any self-care. Totally confined to bed or chair |
| 5 | Death |

Attachment 3 Percentage of Human Bone Marrow Content


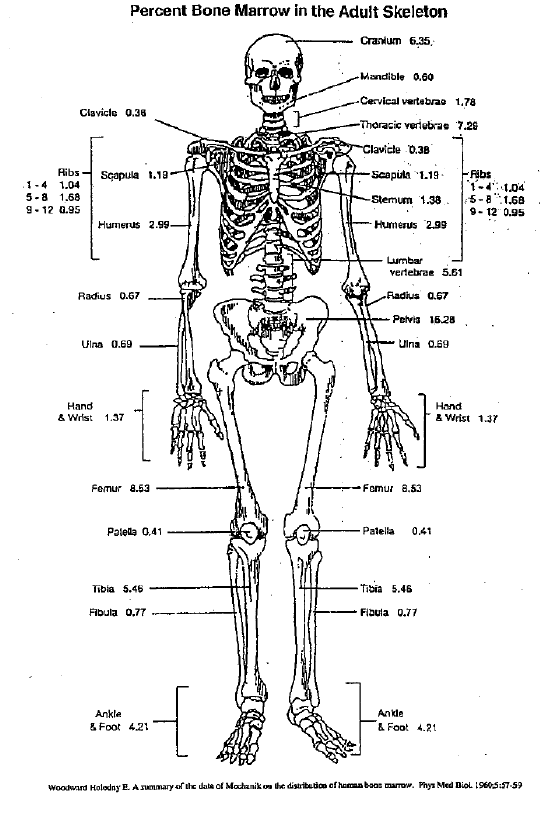


Attachment 4 Prohibited Traditional Chinese Medicine During Study

The prohibited traditional Chinese medicines during study include:

- Huatan Huisheng Tablets
- Brucea javanica oil soft capsule
- Zhe Mu Syrup
- Cantharidin
- Cinobufagin
- Toad venom
- Kangai Injection
- Kanglaite
- Zhongjiefeng Injection
- Aidi Injection
- Awei Huapi Plaster
- Kangaiping Wan
- Fukang Capsules
- XiaoAiPing
- Pingxiao Capsules
- Pingxiao Tablet
- Shendansanjie Capsule
- Ankangxin Capsules
- Bosheng Aining
- Zedoary Turmeric Oil and Glucose Injection
- Kanglixin Capsules
- Cidan Capsules

Attachment 5 TNM Staging of Colorectal Cancer (Version 8)

UICC/AJCC TNM staging system (version 8 of 2017) will be used in this study.

**Primary tumor (T)**

Tx: Primary tumor non-evaluable

T0: No evidence of primary tumor

Tis: Cancer in situ: confined to epithelium or invading lamina propria of mucosa

T1: Tumor invading submucous layer

T2: Tumor invading muscularis propria

T3: Tumor penetrating muscularis propria to subserous layer, or invading paracolorectal tissue without peritoneal coverage

T4a: Tumor penetrating visceral peritoneum

T4b: Tumor directly invading or adhering to other organs or structures

**Regional lymph nodes (N)**

Nx: Regional lymph nodes non-evaluable

N0: No metastasis of regional lymph nodes

N1: Metastasis of 1-3 regional lymph nodes

N1a: Metastasis of 1 regional lymph node

N1b: Metastasis of 2-3 regional lymph nodes

N1c: Tumor deposit (TD) in subserous tissue, mesentery and tissues around colon/rectum without peritoneal coverage, and no metastasis of regional lymph nodes.

N2: Metastasis of above 4 regional lymph nodes

N2a: Metastasis of 4-6 regional lymph nodes

N2b: Metastasis of 7 and more regional lymph node

**Distant metastasis (M)**

Mx: Distant metastasis non-evaluable

M0: Without distant metastasis

M1: With distant metastasis

M1a: Distant metastasis is limited to a single organ (such as liver, lung, ovary, non-regional lymph node), but there is no peritoneal metastasis

M1b: Distant metastasis is distributed in more than one organ

M1c: Peritoneal metastasis with or without metastasis to other organs

**Anatomical stage/prognosis group**

| **Stage** | **T** | **N** | **M** |
| --- | --- | --- | --- |
| Stage 0 | Tis | N0 | M0 |
| Stage I | T1, T2 | N0 | M0 |
| Stage IIA | T3 | N0 | M0 |
| State IIB | T4a | N0 | M0 |
| Stage IIC | T4b | N0 | M0 |
| Stage IIIA | T1-2 | N1/N1c | M0 |
| Stage IIIA | T1 | N2a | M0 |
| State IIIB | T3-T4a | N1/N1c | M0 |
| State IIIB | T2-3 | N2a | M0 |
| State IIIB | T1-2 | N2b | M0 |
| Stage IIIC | T4a | N2a | M0 |
| Stage IIIC | T3-T4a | N2b | M0 |
| Stage IIIC | T4b | N1-N2 | M0 |
| Stage IVA | AnyT | AnyN | M1a |
| State IVB | AnyT | AnyN | M1b |
| Stage IVC | AnyT | AnyN | M1c |

Notes:

1. Tis: Tumor cells are confined to the basement membrane of gland (in epithelium) or lamina propria of mucosa (in mucosa), and do not pass through the muscularis mucosa to the submucosa.
2. T4b: The direct invasion of T4b includes penetrating serosa and invading other intestinal segments, confirmed by microscopic diagnosis (such as cecal cancer invading sigmoid colon), or the tumor located in retroperitoneal or subperitoneal intestine directly invading other organs or structures after penetrating the inherent basal layer of intestinal wall, such as the tumor in the posterior wall of descending colon invading the left kidney or lateral abdominal wall, or the middle and lower rectal cancer invading the prostate, seminal vesicle, cervix or vagina. Visible adhesion of tumors to other organs or structures is Stage cT4b. However, if there is no tumor at the adhesion site microscopically, it is Stage pT3.
3. TD: In case of lymph node metastasis, the number of nodules of tumor deposit is not included in the counting of lymph nodes, but listed separately.
4. Sub-stages V and L: Used to indicate whether there is vascular and lymphatic infiltration, while PNI is used to indicate nerve infiltration (which can be site-specific).
5. CTNM is a clinical stage, while pTNM is a pathological stage; the prefix y is used for tumor staging (such as ypTNM) after receiving neoadjuvant (preoperative) treatment, and the stage of patients with complete response pathologically is ypT0N0 cM0, which may be similar to Stage 0 or Stage 1. The prefix r is used for patients with recurrence after a tumor-free interval after treatment (rTNM)
